# Impact of Pharmacist-Involved Antimicrobial Stewardship Interventions on Clinical and Process Outcomes in Multidrug-Resistant Organism Infections: A Systematic Review and Meta-Analysis

**DOI:** 10.3390/antibiotics15100955

**Published:** 2026-09-25

**Authors:** Ziad G. Nasr, Alaa Sharafeldin, Mouda Hamdan, Zainab Al-Muhsin, Dima Baker, Dina Abushanab

**Affiliations:** 1 College of Pharmacy, QU Health, Qatar University, Doha P.O. Box 2713, Qatar; as2104162@student.qu.edu.qa (A.S.); db2104000@student.qu.edu.qa (D.B.); 2 QU Health, Qatar University, Doha P.O. Box 2713, Qatar; dina.abushanab@qu.edu.qa

**Keywords:** antimicrobial stewardship, pharmacist, multidrug-resistant organisms, antimicrobial resistance

## Abstract

Background/Objectives: Antimicrobial resistance (AMR) is a major global health threat, and multidrug-resistant organisms (MDROs) are associated with worse outcomes than susceptible infections. Pharmacists are central to antimicrobial stewardship (AMS), yet their impact in confirmed MDRO infections is unclear. We evaluated pharmacist-involved AMS on clinical and process outcomes in MDRO infections and quantified pooled effects on clinical outcomes. Methods: Following PRISMA 2020, seven databases were searched (inception–December 2025) for pharmacist-involved AMS studies in patients with confirmed MDRO infections (Tier A) or in mixed populations from which MDRO-specific outcomes were extractable (Tier B), classified into Tier A, B, or C by outcome extractability. Random-effects meta-analyses were performed a priori for outcomes with ≥2 studies, with fixed-effect sensitivity and GRADE ratings. Results: Forty-nine studies were included (7 Tier A, 6 Tier B, 36 Tier C); quantitative synthesis was restricted to the 13 Tier A/B studies with MDRO-specific outcome data, and certainty of evidence was very low for all five pooled outcomes. Pharmacist involvement consistently improved process outcomes (time to therapy, de-escalation, antimicrobial consumption) and reduced mortality and readmissions in several subgroups. Pooled random effects RRs (95% CI) were: 30-day mortality, 0.64 (0.41–1.00; I^2^ = 28%, non-significant); in-hospital mortality, 0.72 (0.57–0.92; I^2^ = 6%, significant); 30-day readmission, 0.50 (0.31–0.79; I^2^ = 0%, significant); recurrent infection, 0.51 (0.25–1.04); and acute kidney injury, 1.01 (0.54–1.87; both non-significant, I^2^ = 0%). Conclusions: Multicomponent AMS interventions involving pharmacists were consistently associated with improved stewardship processes and significant reductions in in-hospital mortality and 30-day readmission, though evidence certainty remained very low. Larger, prospective studies with harmonized outcome definitions are needed to confirm the clinical benefit’s magnitude.

## 1. Introduction

Antimicrobial resistance (AMR) is recognized as a global health emergency because it reduces the effectiveness of antimicrobial therapy and threatens the safety of routine medical care [1]. The World Health Organization has repeatedly identified AMR as a major public health priority that requires coordinated action across human health, animal health, agriculture, and the environment [1]. A recent global analysis estimated that bacterial AMR was associated with millions of deaths worldwide and projected that the global burden will remain substantial through 2050 if effective mitigation strategies are not sustained [2]. The economic consequences of AMR are also considerable because resistant infections increase healthcare utilization, prolong hospitalization, and require more expensive or toxic antimicrobial therapy [3]. Multidrug-Resistant Organism (MDRO) infections represent one of the most clinically important manifestations of AMR because these infections are more difficult to treat and are associated with worse outcomes than infections caused by susceptible organisms [4]. MDROs include Extended-Spectrum β-lactamase-producing Enterobacterales (ESBL-E), Carbapenem-Resistant Enterobacterales (CRE), multidrug-resistant (MDR) and Carbapenem-Resistant *Acinetobacter baumannii* (CRAB), *Pseudomonas aeruginosa* with difficult-to-treat resistance (DTR), Methicillin-Resistant *Staphylococcus aureus* (MRSA), Vancomycin-Resistant *Enterococcus* (VRE), amongst others [5,6]. These pathogens disproportionately affect severely ill, device-exposed, and antimicrobial-exposed hospitalized patients, and often lead to delayed or complex treatment decisions, prolonged bacteremia, treatment failure, higher mortality, and readmission with resistant infection, while also demanding careful selection, dosing, and monitoring of last-line agents such as carbapenems, polymyxins, aminoglycosides, or newer beta-lactam/beta-lactamase inhibitor combinations [7,8]. These challenges make MDRO infections an ideal context in which pharmacist involvement may provide measurable clinical and process benefits [9].

Antimicrobial stewardship programs (ASPs), the organizational structures through which AMS activities are delivered, were developed to optimize antimicrobial use and improve clinical outcomes while limiting resistance selection, adverse drug events, and unnecessary costs [10]. Hospital-based ASPs have been associated with improved process measures, such as antimicrobial appropriateness, de-escalation rates, time to optimal therapy, and reduced antimicrobial consumption, as well as clinical measures, including fewer adverse events, shorter length of stay, and improved infection-related outcomes [11,12,13,14]. International guidelines call for AMS programs to be built on multidisciplinary expertise and institutional leadership support, with pharmacists recognized as central leaders given their pharmacotherapeutic expertise and direct involvement in medication-use systems [15]. Core stewardship elements enhancing AMS include prospective audit and feedback, formulary restriction, preauthorization, guideline development, and education [16,17]. Within this structure, pharmacists commonly contribute through activities such as dose optimization, antimicrobial de-escalation, and therapeutic drug monitoring [18,19]. Evidence from broader AMS literature indicates that pharmacist-led and pharmacist-supported programs can reduce antimicrobial exposure and improve clinical or economic outcomes [20,21,22]. Although AMS evidence is extensive, the specific contribution of pharmacists to MDRO-confirmed infections remains less clearly defined. This distinction is important because resistant infections often require rapid organism-directed therapy, careful monitoring, de-escalation when possible, and coordination across microbiology, infectious diseases, pharmacy, and primary treating teams [15,23].

Although several studies have evaluated pharmacist involvement in AMS among patients with MDRO infections [9,24,25], no previous systematic review has quantitatively synthesized the effect of these interventions on mortality, recurrence, readmission, and adverse outcomes specifically within MDRO populations. Therefore, in addition to narrative synthesis, this review performed a meta-analysis of available comparative studies to estimate pooled clinical effects of pharmacist-involved AMS interventions. The primary objective of this systematic review and meta-analysis was to evaluate the impact of pharmacist-involved AMS interventions on clinical and process outcomes among patients with confirmed or extractable MDRO infections. Secondary objectives included quantifying pooled effects on mortality, recurrence, readmission, and adverse events and exploring the influence of different pharmacist stewardship models.

## 2. Results

### 2.1. Study Selection and Evidence Base

A total of 16,776 records were identified through electronic database searching. After removal of 8581 duplicates, 8195 records remained for title and abstract screening, of which 7895 were excluded for reasons including wrong study design or wrong outcomes. The remaining 300 full-text articles were retrieved and assessed in detail, of which 251 were excluded: lack of confirmed MDRO infection (n = 49), no pharmacist involvement (n = 39), review articles or grey literature (n = 148), non-English language (n = 1), full text not available (n = 11), non-eligible comparator/control group (n = 2), and non-AMS or irrelevant outcome scope (n = 1). Forty-nine studies met all pre-specified eligibility criteria and were included in the review (Figure 1). Studies included 7 Tier A studies [26,27,28,29,30,31,32], 6 Tier B studies [33,34,35,36,37,38], and 36 Tier C studies [Supplementary File S1]. Tier A studies enrolled exclusively patients with microbiologically confirmed MDRO infections or reported outcomes entirely within an MDRO-defined cohort [26,27,28,29,30,31,32]. Tier B studies included mixed populations but provided extractable MDRO-specific or resistant-organism outcomes [33,34,35,36,37,38]. Tier C studies evaluated pharmacist-involved AMS interventions in mixed infection populations without extractable MDRO-specific outcomes [Supplementary File S1]. Tier A and B studies formed the main evidence base for organism-specific synthesis because their outcomes could be directly linked to MDRO infection status [26,27,28,29,30,31,32,33,34,35,36,37,38]. Characteristics of individual studies can be found in Table 1. Tier C studies were kept for contextual interpretation because they supported broader conclusions about pharmacist-involved AMS interventions, but they were not used to infer organism-specific estimates. Detailed data related to Tier C studies can be found in Supplementary File S1.

### 2.2. Characteristics of Included Tier A and Tier B Studies

Tier A and Tier B studies were conducted in diverse settings, including community hospitals, tertiary hospitals, emergency departments, intensive care units, outpatient parenteral antimicrobial therapy services, and multidisciplinary stewardship programs [26,27,28,29,30,31,32,33,34,35,36,37,38]. The most frequently represented infections were MRSA bacteremia, ESBL-producing Enterobacterales infections, resistant Gram-negative bloodstream infections, complicated urinary tract infections, Carbapenem-Resistant-Enterobacterales infections, multidrug-resistant *Pseudomonas aeruginosa* infection, and mixed MDRO cohorts [26,27,28,29,30,31,32,33,34,35,36,37,38]. The pharmacist roles varied across studies and included prospective audit and feedback, post-prescription review, ward pharmacist collaboration, therapeutic drug monitoring, rapid diagnostic test response, culture follow-up, de-escalation recommendations, dosing assessment, and bundle-based care implementation [26,27,28,29,30,31,32,33,34,35,36,37,38].

### 2.3. Risk of Bias Assessment

Overall methodological quality was variable across the included studies. Most were retrospective cohort or pre-post designs, with few randomized or prospective controlled studies. Common methodological limitations included lack of adjustment for confounding, absence of blinding, unclear definitions of exposure or outcomes, small sample sizes, and incomplete reporting of MDRO-specific subgroup analyses in Tier B studies. Tier A studies generally demonstrated higher methodological clarity due to their MDRO-specific populations, whereas Tier B studies carried lower certainty because MDRO outcomes were extracted from broader, mixed populations. Nevertheless, outcome measurement reliability (mortality, length of stay, recurrence, and time to therapy) was consistently high across studies. Overall quality appraisal ratings for each included study are presented in Table 2; domain-level Joanna Briggs Institute (JBI) appraisal ratings underlying each overall rating are provided in Supplementary File S2.

### 2.4. Axis 1: Findings by MDRO or Infection Type

#### 2.4.1. MRSA and Resistant Gram-Positive Infections

Ohashi et al. evaluated patients with MRSA bacteremia following implementation of an ASP with pharmacist intervention, combining early therapy optimization, blood culture follow-up, therapeutic drug monitoring, and bundle-based management [26]. The intervention was associated with a reduction in 30-day mortality from 41.9% to 21.6% and a reduction in hospital mortality from 58.1% to 27.5%, along with improved appropriateness of treatment duration [26]. Smith et al. evaluated a pharmacist-driven care package for *Staphylococcus aureus* bacteremia in a large community healthcare network, including a substantial proportion of MRSA cases [35]. The intervention improved time to appropriate therapy and care bundle adherence, while 30-day readmission was significantly lower in the pharmacist-involved group (RR 0.33, 95% CI 0.12–0.89); mortality did not differ significantly between groups [35].

#### 2.4.2. CRE, MDR *Pseudomonas Aeruginosa*, and Resistant Gram-Negative Infections

McCrink et al. evaluated a real-time diagnostic and stewardship workflow in infections caused by multidrug-resistant Gram-negative organisms, including CRE and MDR *Pseudomonas aeruginosa* [31]. The pharmacist-involved workflow significantly reduced time to appropriate therapy [31]. No statistically significant differences were observed in mortality, bacteremia clearance, hospital length of stay, or AKI [31]. Yamada et al. examined an AMS team intervention incorporating audit and feedback and antibiotic time-out activities for resistant Gram-negative bacteria, reporting a reduction in 30-day mortality, reduced carbapenem use, high acceptance of de-escalation recommendations, and improved clinical and microbiological outcomes [27]. Rinaldi et al. evaluated a multidisciplinary management team incorporating pharmacokinetic and pharmacodynamic optimization for ICU patients with Gram-negative bloodstream infections [38]. Mortality and hospital length of stay were similar between the pre- and post-intervention periods [38].

#### 2.4.3. ESBL- and AmpC-Producing Enterobacterales

Wong et al. evaluated an outpatient parenteral antimicrobial therapy stewardship initiative for uncomplicated ESBL Enterobacterales urinary tract infections, in which the pharmacist-driven intervention consisted of recommending a switch from ertapenem (comparator arm, n = 167) to an aminoglycoside-based regimen (intervention arm, n = 17) to preserve carbapenem use; the study reported a 19% reduction in ertapenem DOT/1000 adjusted patient-days, with no increase in relapse or adverse events [29]. Given the marked imbalance in arm sizes, the relapse and adverse-event comparisons drawn from this study should be interpreted cautiously. Swingler et al. evaluated a pharmacy-driven rapid bacteremia response program for ESBL-producing Enterobacterales bacteremia and did not show significant improvements in in-hospital or 30-day mortality [32]. In addition, Sadyrbaeva-Dolgova et al. evaluated pharmacist recommendations for carbapenem de-escalation in complicated urinary tract infection within an AMS program, reporting lower all-cause mortality and shorter hospital length of stay in the de-escalation group compared with the non-de-escalation group [36]. Lastly, Knudsen and Andersen evaluated a multidisciplinary intervention for ESBL- and AmpC-producing Gram-negative bacteria and reported improved clinical cure with a numerical reduction in mortality [33].

#### 2.4.4. Mixed MDRO Cohorts

Li et al. conducted a multicenter prospective cohort study evaluating pharmacist-driven AMS across intensive care units in eastern China, encompassing a broad case-mix of infections including several MDROs [34]. The pharmacist-driven program was associated with lower hospital mortality (19.3% [68/353] vs. 29.0% [65/224]; adjusted OR 0.57, 95% CI 0.36–0.91) and a lower rate of multidrug-resistant organism acquisition (23.8% vs. 31.7%) [34]. This mortality comparison contributed to the in-hospital mortality meta-analysis in Section 2.9.2 using the crude risk ratio calculated from these counts (RR 0.66, 95% CI 0.49–0.89), consistent in direction and magnitude with the study’s own adjusted estimate [34]. Mahrous et al. evaluated the addition of pharmacist-directed stewardship guidance to multiplex blood-culture rapid diagnostic testing in patients with bacteremia, spanning a mixed Gram-positive and Gram-negative MDRO case-mix [37]. Compared with rapid diagnostic testing alone, the addition of pharmacist-directed guidance was associated with lower in-hospital mortality (11.0% vs. 18.0%; RR 0.61, 95% CI 0.34–1.11) and lower 30-day readmission (13.4% vs. 24.0%; RR 0.56, 95% CI 0.33–0.94) [37].

Nakamura et al. evaluated an ASP in a 126-bed community hospital including patients with several MDROs, including MRSA, ESBL-producing organisms, CRE, VRE, multidrug-resistant *Acinetobacter baumannii*, and multidrug-resistant *Pseudomonas aeruginosa* [28]. Recurrence and relapse decreased, de-escalation and therapeutic drug monitoring increased, and mortality increased in the post-intervention period [28]. Andrade et al. evaluated expansion of a pharmacist-led culture follow-up program to real-time notification of MDRO microbiology results in the emergency department, reporting a reduction in 30-day emergency department revisits or readmissions from 28.3% to 12.9% [30].

### 2.5. Axis 2: Findings by Pharmacist Intervention Model

#### 2.5.1. Bundle-Based Stewardship

Bundle-based stewardship interventions combining multiple evidence-based practices were represented in MRSA bacteremia, ESBL/AmpC infection, and Gram-negative bloodstream infection [26,33,35,38]. Ohashi et al. combined early therapy optimization, blood culture follow-up, therapeutic drug monitoring, and bundle-based management for MRSA bacteremia, reporting reduced 30-day and hospital mortality and improved treatment-duration appropriateness [26]. Smith et al. implemented a pharmacist-driven care package for *Staphylococcus aureus* bacteremia, improving time to appropriate therapy and care bundle adherence, while 30-day readmission was lower in the pharmacist-involved group [35]. Knudsen and Andersen implemented a multidisciplinary bundle combining enhanced microbiological screening, contact precautions, antibiotic restriction, and guideline-directed prescribing for ESBL- and AmpC-producing Gram-negative bacteria, reporting improved clinical cure and a numerical reduction in mortality [33]. Rinaldi et al. incorporated pharmacokinetic and pharmacodynamic optimization into a multidisciplinary management team for ICU patients with Gram-negative bloodstream infections, reporting similar mortality and length of stay between the pre- and post-intervention periods [38].

#### 2.5.2. Rapid Diagnostic and Pharmacist Response Workflows

Rapid diagnostic and pharmacist response workflows were evaluated in CRE/MDR *Pseudomonas*, ESBL, and mixed bacteremia [31,32,37]. McCrink et al. demonstrated a significant reduction in time to appropriate therapy in CRE and MDR *Pseudomonas* infections, with no significant difference in mortality, bacteremia clearance, hospital length of stay, or AKI [31]. Swingler et al. evaluated a pharmacy-driven rapid bacteremia response program for ESBL-producing Enterobacterales bacteremia, reporting no significant improvement in in-hospital or 30-day mortality [32]. Mahrous et al. reported lower in-hospital mortality and lower 30-day readmission following pharmacist-directed guidance added to blood culture rapid diagnostic testing [37].

#### 2.5.3. De-Escalation, Audit and Feedback, and Antibiotic Time-Out

De-escalation, audit and feedback, and antibiotic time-out interventions were evaluated in resistant Gram-negative infection, mixed MDRO ICU cohorts, and complicated urinary tract infection [27,28,34,36]. Yamada et al. reported high de-escalation acceptance, reduced carbapenem use, a reduction in 30-day mortality, and improved clinical and microbiological outcomes following audit and feedback and antibiotic time-out activities [27]. Nakamura et al. reported increased de-escalation and therapeutic drug monitoring, decreased recurrence and relapse, and increased mortality in the post-intervention period following a communication-intensive post-prescription audit model [28]. Li et al. reported lower hospital mortality and lower multidrug-resistant organism acquisition following a prospective audit-and-feedback model across multiple ICUs [34]. Sadyrbaeva-Dolgova et al. reported lower all-cause mortality and shorter hospital length of stay in patients who underwent pharmacist-recommended carbapenem de-escalation for complicated urinary tract infection [36].

#### 2.5.4. Culture Follow-Up and Transitions of Care

Culture follow-up and transitions-of-care interventions were evaluated in outpatient ESBL urinary tract infection and emergency department settings [29,30]. Wong et al. evaluated an outpatient parenteral antimicrobial therapy stewardship initiative for uncomplicated ESBL urinary tract infection, reporting a 19% reduction in ertapenem DOT/1000 adjusted patient-days with no increase in relapse or adverse events [29]. Andrade et al. evaluated expansion of a pharmacist-led culture follow-up program to real-time notification of MDRO microbiology results in the emergency department, reporting a reduction in 30-day emergency department revisits or readmissions from 28.3% to 12.9% [30].

#### 2.5.5. Therapeutic Drug Monitoring and PK/PD Optimization

Rinaldi et al. incorporated therapeutic drug monitoring and pharmacokinetic/pharmacodynamic (PK/PD) optimization into a multidisciplinary management team for ICU patients with Gram-negative bloodstream infections, with pharmacists guiding dose and interval adjustments based on individual patient PK/PD targets [38]. Mortality and length of stay were not significantly different between the pre- and post-intervention periods [38].

### 2.6. Summary of Clinical Outcomes

Across the 13 included studies, mortality findings varied by organism, intervention model, and population. Ohashi et al. and Yamada et al. reported reductions in mortality following bundle-based and audit-and-feedback/antibiotic time-out interventions, respectively [26,27]. McCrink et al., Rinaldi et al., Swingler et al., and Smith et al. found no significant mortality difference [31,32,35,38]. Li et al. reported lower hospital mortality in the pharmacist-involved group [34], while Nakamura et al. reported increased mortality in the post-intervention period [28]. Clinical cure and microbiological or infection-resolution outcomes were reported by several studies, with Yamada et al. reporting improved clinical and microbiological outcomes [27], McCrink et al. finding no significant difference in bacteremia clearance [31], and Sadyrbaeva-Dolgova et al. and Knudsen and Andersen reporting improved clinical cure [33,36]. Recurrence and relapse decreased following Nakamura et al.’s intervention [28], and Wong et al. reported no increase in relapse or adverse events [29]. Length of stay did not differ significantly in McCrink et al. or Rinaldi et al. [31,38], was shorter in the de-escalation group in Sadyrbaeva-Dolgova et al. [36], and was reduced in the ICU setting in Mahrous et al. [37]. Readmission and ED revisit outcomes were mixed, with Andrade et al. and Mahrous et al. reporting reductions [30,37], and Smith et al. reporting a lower 30-day readmission rate in the pharmacist-involved subgroup [35]. AKI did not differ between groups in McCrink et al. or Wong et al. [29,31], and Li et al. reported lower multidrug-resistant organism acquisition at the whole-cohort level [34]. Because outcome definitions, timing, and populations varied across studies, only five clinical outcomes reported by two or more studies with comparable definitions could be quantitatively pooled, with results presented in the meta-analysis section below.

### 2.7. Summary of Process Outcomes

Time to appropriate or targeted therapy improved following pharmacist-involved rapid diagnostic workflows in McCrink et al. and after the addition of pharmacist-directed guidance to rapid diagnostic testing in Mahrous et al. [31,37]. Antimicrobial utilization improved across several studies. Yamada et al. reported reduced carbapenem use alongside high acceptance of de-escalation recommendations [27]. Wong et al. reported a 19% reduction in ertapenem days of therapy per 1000 adjusted patient-days for outpatient ESBL urinary tract infection [29]. Sadyrbaeva-Dolgova et al. and Nakamura et al. reported increased carbapenem or broad-spectrum de-escalation, with Nakamura et al. additionally reporting increased therapeutic drug monitoring [28,36]. Bundle adherence and treatment-duration appropriateness improved in the MRSA-focused studies, with Ohashi et al. reporting improved appropriateness of treatment duration [26] and Smith et al. reporting improved care-bundle adherence [35]. Rinaldi et al. incorporated pharmacokinetic/pharmacodynamic-guided dose optimization into routine multidisciplinary ICU care [38]. A standardized summary of process outcomes across all 13 Tier A/B studies, including time to appropriate therapy, de-escalation and escalation rates, antimicrobial utilization, and culture follow-up/TDM data, is provided in Supplementary File S3.

### 2.8. Cross-Cutting Findings

Across the organism-specific findings in Axis 1 and the intervention-model findings in Axis 2, most studies reported improvement in at least one process measure (time to appropriate therapy, antimicrobial utilization, de-escalation, bundle adherence, or TDM), while mortality, recurrence, and readmission findings varied in magnitude and direction across studies. Mortality reductions were reported for bundle-based MRSA bacteremia management (Ohashi et al.) and audit-and-feedback/antibiotic time-out for resistant Gram-negative infection (Yamada et al.) [26,27], whereas rapid diagnostic and PK/PD-based interventions improved process measures without a significant mortality effect [31,38]. Li et al. found a mortality benefit for pharmacist-led ICU stewardship, consistent with the lower in-hospital mortality observed in the pooled analysis [34]. Likewise, Smith et al. reported lower 30-day readmission rates among patients in the pharmacist-involved group [35]. After standardizing the intervention direction across studies, the pooled results showed consistent effects with minimal heterogeneity for both in-hospital mortality (I^2^ = 6%) and 30-day readmission (I^2^ = 0%). McCrink et al. and Mahrous et al. both reported improved time to appropriate therapy following pharmacist-directed guidance after rapid diagnostic testing, with Mahrous et al. also reporting reduced mortality and readmission [31,37]. Nearly all included studies used quasi-experimental, pre-post, or observational cohort designs rather than randomization [26,27,28,29,30,31,32,33,34,35,36,37,38].

### 2.9. Key Outcomes Reported by Included Studies—Quantitative Synthesis (Meta-Analysis)

Of the 49 included studies, only the 13 Tier A/B studies contributed MDRO-specific outcome data eligible for quantitative pooling; each individual meta-analysis was based on only two to four of these 13 studies (30-day mortality: 3; in-hospital mortality: 4; recurrent infection: 2; 30-day readmission: 2; acute kidney injury: 2). Detailed outcome data for all 13 studies, incorporating raw counts, denominators, and effect sizes clarified during data extraction, are summarized in Table 3.

**Table 3:** Key Outcomes Reported by Included Studies.

No.	Study	Outcome	Type	Result (Comparator vs. Intervention)	Effect Size (95% CI)
1	Ohashi et al. [26]	Early anti-MRSA therapy	Binary	62.3% → 82.4%, *p* = 0.038	NR
		Appropriate treatment duration	Binary	44.8% → 72.1%, *p* = 0.027	NR
		Negative follow-up cultures	Binary	40% → 80%, *p* < 0.001	NR
		30-day mortality	Binary	18/43 (41.9%) vs. 11/51 (21.6%)	OR ‡ 0.33 (0.12–0.86)
		Hospital mortality	Binary	25/43 (58.1%) vs. 14/51 (27.5%)	OR ‡ 0.20 (0.07–0.53)
2	McCrink et al. [31]	30-day mortality	Binary	12/53 (22.6%) vs. 11/46 (23.9%), *p* = 0.881	OR 0.93 (0.36–2.40)
		Time to bacteremia clearance	Continuous	Median [IQR]: 74 h [59–98] vs. 67 h [44–114], *p* = 0.841	NR
		Hospital LOS	Continuous	As reported (values internally inconsistent in source table), *p* = 0.180	NR
		Time to appropriate therapy	Continuous	Median [IQR]: 103 h [76–156] vs. 75 h [56–100], *p* < 0.001	NR
		AKI	Binary	15/53 (28.3%) vs. 13/46 (28.3%), *p* = 0.996	OR 1.00 (0.42–2.43)
3	Swingler et al. [32]	Time to active therapy	Continuous	Median [IQR]: 22.8 h [3.7–63.8] → 16.4 h [2.9–19.3], *p* = 0.001	NR
		In-hospital mortality	Binary	19% → 17%, *p* = 0.854 (NS); raw counts NR	NR
		30-day mortality	Binary	20% → 21%, *p* > 0.999 (NS); raw counts NR	NR
4	Wong et al. [29]	Relapse (ESBL-EB UTI)	Binary	Ertapenem 46/167 (28%) vs. aminoglycoside 3/17 (18%), *p* = 0.57	NR
		Infection resolution	Binary	All patients achieved clinical improvement; no treatment failure (not further quantified)	NR
		Adverse events	Binary	Ertapenem 5/167 (3%) vs. aminoglycoside 0/17, *p* = 0.99 (no AKI in either group)	NR
		Ertapenem DOT/1000 APD	Continuous	0.0145 (November 2014) → 0.0078 (February 2017), *p* < 0.01; mean monthly DOT 32 → 26 (−19%), *p* < 0.01	NR
5	Yamada et al. [27]	30-day mortality (resistant GNB)	Binary	19/93 (20.4%) vs. 13/124 (10.5%), *p* = 0.04	RR † 0.51 (0.27–0.98)
		Hospital mortality (resistant GNB)	Binary	26/93 (28.0%) vs. 20/124 (16.1%)	RR † 0.58 (0.34–0.97)
		Appropriate therapy	Binary	Improved (not further quantified)	NR
		TDM	Continuous	Anti-MRSA TDM monitored; no outcome-impact data reported	NR
		DOT per 1000 patient-days	Continuous	Carbapenems: 33.2 ± 5.7 → 25.5 ± 4.6; TAZ/PIPC: 18 ± 4.8 → 23.3 ± 2.7 (significant increase)	NR
		De-escalation acceptance rate	Binary	133/145 (91.7%)	NR
6	Nakamura et al. [28]	30-day mortality	Binary	11.2% vs. 14.2% (NS); raw counts NR	NR
		30-day recurrence rate	Binary	14.7% (n = 288) → 7.5% (n = 247), *p* < 0.05	NR
		Antimicrobial utilization (AUD/DOT)	Continuous	Carbapenem: 0.37 ± 0.07 → 0.60 ± 0.12, *p* < 0.01; TAZ/PIPC: 0.73 ± 0.08 → 0.67 ± 0.07, *p* = 0.15	NR
		De-escalation rate	Binary	0/288 (pre) vs. 7/247 (post)	NR
		TDM	Continuous	3/288 (pre) vs. 5/247 (post)	NR
7	Andrade et al. [30]	30-day ED revisit/treatment failure	Binary	17/60 (28.3%) vs. 9/70 (12.9%), *p* = 0.046	NR
		Time from positive culture to review	Continuous	Significant reduction, *p* < 0.001 (not further quantified)	NR
8	Smith et al. [35]	All-cause (in-hospital) mortality	Binary	5/86 (5.8%) vs. 5/172 (2.9%), *p* = 0.254	RR † 0.50 (0.15–1.65); OR † 0.48 (0.13–1.77)
		Time to appropriate therapy (≤24 h)	Binary	65/86 (76%) vs. 169/172 (98%), *p* < 0.001; time-to-therapy 14.19 h (8.37–20.01) → 1.48 h (0.79–2.17)	NR
		30-day readmission	Binary	9/83 (10.8%) vs. 6/169 (3.6%), *p* = 0.024	RR † 0.33 (0.12–0.89)
9	Li et al. [34]	All-cause (hospital) mortality	Binary	65/224 (non-pharmacist) vs. 68/353 (pharmacist-driven)	RR † 0.66 (0.49–0.89); OR ‡ 0.57 (0.36–0.91)
		Hospital LOS	Continuous	Median [IQR]: 11 [11–31] vs. 17 [12–29], *p* = 0.544 (group assignment as reported; NS)	NR
		ICU LOS	Continuous	Median [IQR]: 5 [3–10] vs. 4 [3–8], *p* = 0.227	NR
		Antimicrobial consumption (DOT)	Continuous	Median [IQR]: 5 [3–9.5] vs. 4 [2–7], *p* = 0.030	NR
10	Mahrous et al. [37]	30-day readmission	Binary	40/167 (24.0%) vs. 17/127 (13.4%)	RR † 0.56 (0.33–0.94)
		All-cause (in-hospital) mortality	Binary	30/167 (18.0%) vs. 14/127 (11.0%)	RR † 0.61 (0.34–1.11)
		De-escalation rate	Binary	41/164 (25%) vs. 31/148 (21%)	RR † 0.84 (0.56–1.25); OR † 0.80 (0.47–1.36)
		Escalation rate	Binary	23/164 (14%) vs. 13/148 (9%)	RR † 0.63 (0.33–1.18); OR † 0.59 (0.29–1.21)
		Hospital LOS	Continuous	Median (IQR): 8 (5–15) → 6.5 (4–12) days; MRSA subgroup: 13.5 (7.25–19) → 9.5 (8.25–12.75)	NR
		ICU LOS	Continuous	Median (IQR): 6 (4–12) → 4 (2–8) days	NR
		Time to appropriate therapy	Continuous	Median (IQR): 41 (27.7–64) → 18.1 (8.7–27) h; MRSA subgroup: 39 (28.75–52.5) → 9 (0.4–18) h	NR
11	Sadyrbaeva-Dolgova et al. [36]	All-cause mortality	Binary	De-escalation 6/81 (7.4%) vs. no de-escalation 24/82 (29.3%)	OR 0.19 (0.07–0.50); OR ‡ 0.13 (0.04–0.41)
		30-day readmission	Binary	De-escalation 16/81 (19.8%) vs. no de-escalation 12/82 (14.6%)	NR
		De-escalation appropriateness	Binary	Unadjusted, crude, as reported OR 0.40 (0.19–0.84); adjusted, as reported OR ‡ 0.33 (0.15–0.74)	OR 0.40 (0.19–0.84); OR ‡ 0.33 (0.15–0.74)
		Overall DOT (days)	Continuous	Median: de-escalation 13 (10–17) vs. no de-escalation 9 (6–13); carbapenem-specific: 4 (2–6) vs. 6 (4–10)	NR
		Hospital LOS	Continuous	Median (IQR): de-escalation 9 (7–23) vs. no de-escalation 14 (8–30) days	NR
12	Knudsen & Andersen [33]	ESBL-KP incidence	Continuous	Significant reduction, *p* < 0.001 (rate not further quantified)	NR
		30-day mortality (ESBL-KP)	Binary	11/34 (35%) → 5/29 (17%), NS	NR
13	Rinaldi et al. [38]	Clinical cure at 7 days	Binary	31/67 (47.7%) → 51/68 (75%), *p* < 0.001	NR
		All-cause mortality	Binary	24/67 (35.8%) vs. 24/68 (35.3%)	NR
		Hospital LOS	Continuous	42 days (pre) vs. 43 days (post)	NR
		ICU LOS	Continuous	21 days (pre) vs. 18 days (post)	NR
		Meropenem/piperacillin-tazobactam DDD per patient	Continuous	Meropenem: 585 → 232 total DDD, *p* < 0.001; piperacillin-tazobactam: 105 → 168 DDD, *p* < 0.002	NR
		Appropriate days of therapy	Continuous	Median [IQR]: 12 [20] (pre) → 9 [15] (post); 16 → 12 days overall, *p* < 0.001	NR
		Targeted de-escalation	Binary	8/67 (11.9%) → 14/68 (20.6%), NS	NR
		Escalation	Binary	3/67 (4.5%) → 2/68 (2.9%)	NR
		TDM-guided dose reduction	Binary	43/68 (63.2%) post-phase patients	NR
		Relapse (breakthrough/persistent/recurrent BSI)	Binary	Breakthrough 10/67 (14.9%)→4/68 (5.4%); persistent 11/67 (16.4%)→3/68 (4.4%); recurrent BSI 12/67 (17.9%)→5/68 (7.4%)	NR

† Calculated by the review team from raw counts reported in the original study (crude, unadjusted estimate). ‡ Adjusted estimate as reported by the original study; unmarked effect sizes were reported directly by the original study; NR, not reported by the original study and not calculable from the data provided. Abbreviations: AKI, acute kidney injury; BSI, bloodstream infection; DDD, defined daily dose; DOT, days of therapy; ESBL, extended-spectrum beta-lactamase; ICU, intensive care unit; IQR, interquartile range; LOS, length of stay; NS, not significant; OR, odds ratio; RR, risk ratio; TDM, therapeutic drug monitoring; UTI, urinary tract infection. † calculated by the review team, not reported directly by the original study; NR, not reported/not calculable from available data.

#### 2.9.1. 30-Day Mortality

Three studies involving 410 patients were included in the meta-analysis of 30-day mortality (Yamada et al. [27], Ohashi et al. [26], McCrink et al. [31]). Pharmacist-involved AMS interventions were associated with a borderline, non-significant reduction in mortality compared with control using the pre-specified random-effects model (RR 0.64, 95% CI 0.41–1.00; I^2^ = 28%; *p* = 0.05) (Figure 2A). A fixed-effect model applied to the same three studies produced a statistically significant result (RR 0.63, 95% CI 0.43–0.92; *p* = 0.02) (Figure 2B); however, with only three studies contributing, estimates of between-study variance (τ^2^) and I^2^ are inherently imprecise, and the significance of the pooled estimate is sensitive to this choice of model rather than being confirmed by it. Because random effects were specified a priori as the primary model, and the three contributing studies differ in MDRO species and clinical setting (Section 2.4.1 and Section 2.4.2), the fixed-effect result should not be read as strengthening confidence in a mortality benefit; the non-significant random-effects estimate remains the basis for interpretation.

#### 2.9.2. In-Hospital Mortality

Four studies including 1264 patients were included in the exploratory meta-analysis of hospital mortality (Li et al. [34], Mahrous et al. [37], Rinaldi et al. [38], Smith et al. [35]). Pharmacist-involved AMS interventions were associated with a statistically significant reduction in hospital mortality compared with control (RR = 0.72, 95% CI 0.57–0.92; *p* = 0.007; I^2^ = 6%) using a random-effects model (Figure 3A). Sensitivity analysis using a fixed-effect model showed similar results (RR = 0.71, 95% CI 0.56–0.89; *p* = 0.003) (Figure 3B). Given that this estimate is supported exclusively by observational/quasi-experimental evidence and was rated very-low certainty by GRADE (Section 2.9.5 below), the available evidence suggests a possible association between pharmacist-involved AMS and lower in-hospital mortality, but the very low certainty of evidence precludes confident estimation of the magnitude or causality of this association.

#### 2.9.3. Recurrent Infection

Two studies including 319 patients were included in the exploratory meta-analysis of recurrent infection or relapse (Rinaldi et al. [38], Wong et al. [29]). These studies assessed clinically distinct endpoints: relapse of uncomplicated ESBL urinary tract infection (Wong et al.) versus recurrent or persistent Gram-negative bloodstream infection (Rinaldi et al.), which differ in biological mechanism, follow-up requirements, and baseline risk. The pooled estimate is presented for completeness but should not be interpreted as a single unified clinical endpoint. Pharmacist-involved AMS interventions were associated with a lower point estimate for recurrence compared with control, although the result did not reach statistical significance (RR 0.51, 95% CI 0.25–1.04; I^2^ = 0%) using a random-effects model (Figure 4A). Sensitivity analysis using a fixed-effect model produced identical results (Figure 4B).

#### 2.9.4. Thirty-Day Readmission

Two studies including 546 patients were included in the meta-analysis of 30-day readmission (Mahrous et al. [37], Smith et al. [35]). Pharmacist-involved AMS interventions were associated with a statistically significant difference in readmission compared with control (RR 0.50, 95% CI 0.31–0.79; I^2^ = 0%; *p* = 0.003) using a random-effects model (Figure 5A). Sensitivity analysis using a fixed-effect model showed similar significant findings (Figure 5B).

#### 2.9.5. Acute Kidney Injury

Two studies including 283 patients were included in the exploratory meta-analysis of AKI (McCrink et al. [31], Wong et al. [29]). These studies differed in population and settings (CRE/MDR *Pseudomonas aeruginosa* infections managed as inpatients vs. ESBL urinary tract infections managed through OPAT) and antimicrobial exposure. However, similar outcome definitions justified pooling despite the absence of statistical heterogeneity (Wong et al. define AKI as ≥50% increase in serum creatinine (SCr) levels within <7 days, while McCrink et al. similarly define it according to the Acute Kidney Injury Network Classification: a rise in SCr by 0.3 mg/dL within 2 days, or a 50% rise in SCr within 7 days of index culture). Pharmacist-involved AMS interventions were not associated with a significant difference in adverse events compared with control (RR 1.01, 95% CI 0.54–1.87; I^2^ = 0%) using a random-effects model (Figure 6A). Sensitivity analysis using a fixed-effect model produced identical results (RR 1.01, 95% CI 0.54–1.86) (Figure 6B).

#### 2.9.6. Certainty of Evidence for Pooled Outcomes

The five pooled analyses below should be interpreted as exploratory rather than confirmatory. Each combines a small number of studies (two to four) that differ in MDRO species, infection syndrome, clinical setting, and intervention model (detailed in Section 2.9.1, Section 2.9.2, Section 2.9.3, Section 2.9.4 and Section 2.9.5 above), and low statistical heterogeneity (I^2^) in analyses of this size has limited power to detect genuine clinical heterogeneity and should not be read as evidence of clinical homogeneity. Applying GRADE criteria to the five quantitatively pooled outcomes, using the pre-specified random-effects model as the primary analysis for each outcome, yielded a certainty rating of very low for all five outcomes (Table 4), reflecting the exclusively quasi-experimental or observational nature of the contributing studies together with serious imprecision for every pooled estimate. For 30-day mortality, the primary random-effects result (RR 0.64, 95% CI 0.41–1.00) was similar across studies (I^2^ = 28%), but the confidence interval still allowed for no effect. For recurrent infection and acute kidney injury, the evidence remained very low mainly due to imprecision from the small number of studies and events; although there was no statistical heterogeneity, the confidence intervals crossed the null. In contrast, in-hospital mortality (RR 0.72, 95% CI 0.57–0.92; I^2^ = 6%) and 30-day readmission (RR 0.50, 95% CI 0.31–0.79; I^2^ = 0%) showed consistent effects with little to no heterogeneity, yet the overall certainty stayed very low because all contributing studies were observational or quasi-experimental. Publication bias could not be formally assessed for any outcome because fewer than ten studies contributed to each analysis.

### 2.10. Tier C

Thirty-six studies were categorized as Tier C. These studies enrolled mixed populations of MDRO and non-MDRO infections, and outcomes were not reported separately for MDRO-specific subgroups. They were therefore not eligible for the organism-specific synthesis described above. Interventions evaluated among Tier C studies included pharmacist-led intravenous-to-oral conversion, de-escalation, audit and feedback, rapid diagnostics, guideline adherence, and bundle-based care. Not all Tier C studies reported a mortality-related outcome, and among those that did, results varied in both direction and statistical significance. Process and utilization outcomes were reported more consistently, though not uniformly favorable across all studies. Study-by-study outcomes, including the specific direction of effect for each reported measure, are provided in Supplementary File S1.

## 3. Discussion

To our knowledge, this is the first systematic review and meta-analysis to quantitatively synthesize the effects of pharmacist-involved AMS interventions specifically within MDRO-confirmed populations, rather than general or mixed-infection stewardship cohorts. This review and exploratory meta-analysis demonstrate that multicomponent AMS interventions involving pharmacists are consistently associated with improved antimicrobial management among patients with MDRO infections; because most interventions bundled pharmacist activity with other components (e.g., rapid diagnostics, infectious diseases consultation, guideline implementation), the observed associations cannot be attributed to the pharmacist component independently. Across diverse clinical settings, pharmacist participation facilitated earlier initiation of appropriate therapy, optimized antimicrobial selection, increased de-escalation, and reduced broad-spectrum antimicrobial exposure. Studies incorporating rapid diagnostic testing (RDT) alongside pharmacist interpretation demonstrated the largest reductions in time to active therapy, indicating that diagnostic speed alone does not translate into clinical benefit without a clinician positioned to act on it in real time [31,32,37,39]. This benefit appears cumulative rather than substitutive: the diagnostic result narrows the therapeutic options, but it is the pharmacist’s interpretation that converts that narrowing into a bedside decision. This is consistent with Giordano et al.’s observation that clinical decision-support systems synergize with, rather than replace, pharmacist expertise in ASPs [14]. These findings align with broader AMS literature: Dighriri et al. found that clinical pharmacists improve prescribing appropriateness through optimization, dose adjustment, and guideline adherence [18,40], and Okada described the pharmacist’s evolving role from medication reviewer to active participant in antimicrobial decision-making [21]. Al-Omari et al. and Guchhait et al. each reported sustained improvements in stewardship-related process measures over multi-year follow-up [11,12], consistent with reduced antimicrobial days of therapy following education-driven stewardship policies [41] and sustained reductions in antimicrobial consumption and cost following ASP maturation reported elsewhere [42]. As Khadem et al. argue, process measures are not a lesser substitute for clinical outcomes but a direct indicator of prescribing quality in their own right [13]. This is particularly consequential in MDRO management, where delays in effective therapy carry severe downstream consequences [13]. Despite these robust process improvements, the effect on hard clinical endpoints, such as mortality, was more variable: the pooled estimate for 30-day mortality suggested only a borderline decrease that did not reach statistical significance, whereas the pooled analysis for in-hospital mortality showed a statistically significant relative reduction (RR 0.72; absolute risk difference approximately 7 fewer deaths per 100 patients treated, based on the pooled control-arm event rate) associated with pharmacist-involved AMS interventions, though this estimate should be interpreted alongside its very-low GRADE certainty.

Mortality reductions were observed following comprehensive bundle-based stewardship [26] and longitudinal audit-and-feedback programs [27], while McCrink et al., Nakamura et al., and Smith et al. showed no significant mortality difference [28,31,35]. The low heterogeneity seen in the pooled analysis indicates that study results were largely consistent in direction, even though patient characteristics and intervention approaches varied. However, mortality in MDRO infections is driven by multiple factors independent of antimicrobial selection, including baseline illness severity, effectiveness of source control, pathogen virulence, and overall comorbidity burden [4,8]. Even optimal, rapid antimicrobial therapy may be insufficient to overcome an already severe disease trajectory. Because all contributing studies used observational or quasi-experimental designs without randomization, they cannot disentangle the independent contribution of pharmacist involvement from these confounding factors, and the pooled mortality estimates should be interpreted as describing an association within multicomponent AMS interventions rather than an independent pharmacist effect. This pattern is consistent with prior AMS literature, as Naseralallah et al., in a meta-analysis of pharmacist-led perioperative AMS, similarly reported clear process-level improvements alongside substantial between-study heterogeneity that limited firm conclusions regarding clinical outcomes [25].

Findings for healthcare utilization outcomes were more encouraging. The pooled analysis found a statistically significant 50% relative reduction in 30-day readmissions (RR 0.50; absolute risk difference approximately 13 fewer readmissions per 100 patients treated, based on the pooled control-arm event rate), and this benefit was consistent across the included studies, with no evidence of statistical heterogeneity. Andrade et al. similarly reported a reduction in emergency department revisits and treatment failure following expansion of a pharmacist-led culture-notification program (28.3% to 12.9%) [30], and Nakamura et al. reported reduced recurrence and relapse under a bundle-based AMS model [28], though variability in outcome definitions and follow-up windows across studies limits direct comparison. Importantly, these gains did not come at the cost of renal safety, specifically. McCrink et al. found no difference in AKI between intervention and comparator groups (28.3% vs. 28.3%) [31], and the pooled AKI estimate across studies likewise showed no signal of renal harm. This is a meaningful finding, since concern about nephrotoxicity from more aggressive antimicrobial optimization is a plausible barrier to adopting pharmacist-driven de-escalation, and this review’s data do not support that specific concern. However, AKI was the only adverse-event endpoint quantitatively pooled in this review; other potential harms (e.g., *Clostridioides difficile* infection, other antimicrobial toxicities, drug–drug interactions) were not systematically assessed across studies, and this finding should not be generalized to overall patient safety.

The interpretation of all the above must be tempered by the certainty of the underlying evidence. Every pooled outcome in this review (mortality, recurrence, readmission, and AKI) was rated “very low” certainty by GRADE, reflecting the near-total reliance on observational and quasi-experimental designs, which are inherently vulnerable to confounding by indication and to secular trends occurring alongside the intervention (e.g., concurrent infection-control initiatives, changing local resistance patterns). This certainty rating should be read as a statement about the evidence base’s design, not as a judgment on the intervention’s plausibility. The direction and consistency of the process-outcome findings, replicated across markedly different settings and MDRO populations, is itself a form of evidence, even where the underlying study designs cap how much confidence GRADE allows us to place in any single clinical-outcome estimate. Confirming these signals will require the kind of prospective, randomized designs that GRADE rewards with higher certainty ranking; a gap that the current evidence base cannot close on its own.

This review has several limitations. The search was restricted to English-language publications, which may have excluded relevant non-English evidence. Publication bias could not be formally assessed for any pooled outcome because fewer than ten studies contributed to each analysis. The small number of studies per outcome (two to four) limits the precision and generalizability of the pooled estimates, and outcome definitions, MDRO species, infection syndromes, settings, intervention models, and study design (pre-post/historical-control vs. concurrent-comparator) varied across the studies contributing to each pooled analysis despite efforts to extract comparable data. Four of the five pooled outcomes (in-hospital mortality, recurrent infection, 30-day readmission, and AKI) combine pre-post studies, which are vulnerable to secular trends and concurrent infection-control initiatives, with concurrent-comparator studies, and a design-stratified sensitivity analysis to test whether pre-post studies were driving the pooled effects was not performed. The low I^2^ observed for several outcomes reflects limited statistical power to detect heterogeneity in such small samples and should not be interpreted as evidence of clinical homogeneity across the pooled studies. Finally, Tier C studies, though numerous, could not contribute to quantitative synthesis because MDRO-specific outcomes were not separable from mixed populations, limiting the evidence base available for pooling to the 13 Tier A and B studies. Because certainty for every pooled outcome in this review was downgraded largely on the basis of reliance on observational and quasi-experimental designs, future studies in this area could meaningfully improve the certainty of subsequent evidence syntheses. Adopting more standardized data-collection protocols, systematically measuring and adjusting for known confounders of MDRO outcomes (e.g., baseline illness severity, source control, comorbidity burden), collecting data prospectively rather than relying on retrospective or historical-control designs where feasible, and using harmonized, pre-specified outcome definitions (e.g., a consistent AKI definition, a consistent recurrence/relapse window) would make future pooled estimates less vulnerable to the indirectness and design-heterogeneity concerns raised elsewhere in this review. Taken together, this review is consistent with a role for pharmacists within multidisciplinary AMS teams managing MDRO infections, without evidence of compensatory harm. Future work should move beyond further single-center pre-post studies that dominate this literature toward prospective, ideally cluster-randomized, multicenter trials using harmonized MDRO-specific outcome definitions, so that the clinical and economic value of pharmacist-led AMS can be quantified with the certainty this body of evidence otherwise deserves. Three evidence gaps merit particular priority: isolating the pharmacist-specific contribution to mortality from concurrent, bundled interventions; building organism-specific cohorts large enough to pool recurrent infection and AKI without the population heterogeneity noted above; and formally testing whether process measures such as time to appropriate therapy mediate the clinical benefits observed, rather than reporting the two as parallel findings. Sustaining this role will also depend on continued investment in stewardship-focused pharmacy education to prepare the workforce delivering these interventions [43].

## 4. Materials and Methods

### 4.1. Study Design, Reporting Framework, and Protocol Registration

This systematic review was designed to evaluate the impact of pharmacist-involved AMS interventions on clinical and process outcomes in patients with MDRO infections. The review followed PRISMA 2020 principles for identification, screening, eligibility assessment, and reporting [44]. The review question was developed using the PICO framework, with hospitalized or healthcare-managed patients with confirmed MDRO infection as the population, pharmacist-led or pharmacist-involved AMS as the intervention, standard care or non-pharmacist AMS as the comparator, and clinical or process outcomes as the outcomes of interest. The study protocol (ID: CRD420251153016) was registered on the PROSPERO database: https://www.crd.york.ac.uk/PROSPERO/view/CRD420251153016 (accessed on 27 September 2025).

### 4.2. Eligibility Criteria

Studies were eligible if they evaluated patients with microbiologically confirmed MDRO infections, operationally defined as non-susceptibility to at least one agent in three or more antimicrobial categories (consistent with consensus criteria from the European Centre for Disease Prevention and Control (ECDC) and the Centers for Disease Control and Prevention (CDC)), or mixed populations in which resistant-organism outcomes could be extracted [45]. Organism-specific resistance definitions (e.g., ESBL, CRE, MRSA) were accepted as reported by each primary study. Eligible organisms included MRSA, VRE, ESBL-producing Enterobacterales, CRE, carbapenem-resistant or multidrug-resistant *Acinetobacter baumannii*, multidrug-resistant or difficult-to-treat *Pseudomonas aeruginosa*, and other resistant Gram-negative organisms. Eligible interventions included pharmacist-led, pharmacist-driven, or pharmacist-involved AMS activities such as audit and feedback, de-escalation, dose optimization, therapeutic drug monitoring, rapid diagnostic response, culture follow-up, guideline implementation, multidisciplinary rounds, OPAT stewardship, and bundle-based care. Eligible comparators included usual care, historical control periods, pre-intervention periods, or AMS models without the same pharmacist intervention. Eligible study designs included randomized trials, quasi-experimental studies, interrupted time-series studies, cohort studies, case–control studies, and observational comparative studies. Reviews, editorials, case reports, case series without comparative data, conference abstracts, non-English publications, and studies without pharmacist involvement were excluded.

### 4.3. Information Sources and Search Strategy

A comprehensive search was conducted in PubMed, Embase, Scopus, Web of Science, CINAHL, ERIC, and ProQuest from database inception to December 2025. The search strategy combined controlled vocabulary and free-text terms for antimicrobial stewardship, pharmacists, and MDROs. Search terms included concepts related to antimicrobial stewardship, antibiotic stewardship, pharmacist, clinical pharmacist, pharmacy-driven, audit and feedback, de-escalation, rapid diagnostics, therapeutic drug monitoring, multidrug-resistant organism, MRSA, ESBL, CRE, VRE, *Acinetobacter*, *Pseudomonas*, and resistant Gram-negative infection. Search syntax was adapted to each database according to its indexing structure; the complete, line-by-line search strategy for each of the seven databases is provided in Supplementary File S4. Reference lists of included studies and relevant reviews were also screened to identify additional eligible studies.

### 4.4. Study Selection Process

All retrieved records were imported into Rayyan for screening and duplicate removal. Title and abstract screening was performed independently by reviewer pairs using prespecified eligibility criteria. Studies that clearly did not involve pharmacist-led or pharmacist-involved AMS, did not include resistant infections, or did not report relevant outcomes were excluded at this stage. Full texts were then reviewed independently by reviewers to confirm eligibility. Disagreements were resolved through discussion and, when needed, consultation with an additional reviewer. Studies were retained if they met the intervention, population, and outcome criteria and provided sufficient information for extraction.

### 4.5. Data Extraction

Data were extracted independently by four study team members using a standardized data extraction form, with disagreements resolved by discussion. Extracted study characteristics included author, publication year, country, setting, study design, sample size, and study period. Extracted patient and infection characteristics included age group, infection syndrome, organism type, MDRO definition, and whether the population was fully MDRO-specific or mixed. Extracted intervention characteristics included the pharmacist role, stewardship model, timing of intervention, team composition, diagnostic tools, and comparator. Extracted outcomes included all-cause mortality, infection-related mortality where reported, clinical cure or infection resolution, recurrence, relapse, readmission, hospital length of stay, ICU length of stay, time to appropriate therapy, time to targeted therapy, antimicrobial DOT or DDD, de-escalation, escalation, therapeutic drug monitoring, adverse drug events, and cost outcomes. All extracted data were reviewed by the lead principal investigator (LPI) for consistency and accuracy before analysis.

### 4.6. Tier Classification

Included studies were classified into three tiers according to MDRO outcome extractability. Tier A studies included populations in which all patients had confirmed MDRO infections or studies in which reported outcomes were fully MDRO-specific. Tier B studies enrolled mixed populations of MDRO and non-MDRO infections but reported at least some outcomes specifically for the MDRO-infected subgroup, which could be extracted separately from the non-MDRO subgroup. Tier C studies included mixed populations in which MDRO-specific outcomes could not be separated from non-MDRO outcomes. Tier A and Tier B studies were used for the main organism-specific synthesis, while Tier C studies were used to contextualize broader pharmacist-led AMS effects.

### 4.7. Axis-Based Synthesis Framework

A two-axis synthesis framework was used to organize the narrative findings. Axis 1 grouped studies by MDRO type or infection category, including MRSA and resistant Gram-positive infections, ESBL/AmpC-producing Enterobacterales, CRE and MDR *Pseudomonas*, resistant Gram-negative bloodstream infections, and mixed MDRO cohorts. Axis 2 grouped studies by pharmacist intervention model, including bundle-based stewardship, rapid diagnostic and pharmacist response workflows, audit and feedback or antibiotic time-out, de-escalation programs, therapeutic drug monitoring and PK/PD optimization, OPAT stewardship, and culture follow-up or transitions-of-care interventions. This dual-axis structure allowed the review to identify whether outcomes differed by organism type, infection syndrome, and stewardship mechanism.

### 4.8. Outcomes

The primary clinical outcomes were all-cause mortality, infection resolution or clinical cure, recurrence or relapse, and hospital or ICU length of stay. Secondary process outcomes included time to appropriate therapy, time to targeted therapy, antimicrobial utilization measured by DOT or DDD, de-escalation, escalation, therapeutic drug monitoring, guideline adherence, and acceptance of pharmacist recommendations. Additional outcomes included readmission, emergency department revisit, adverse drug events, acute kidney injury, and antimicrobial cost. Outcomes were extracted as reported by each study and were not pooled statistically because of heterogeneity in populations, interventions, and outcome definitions.

### 4.9. Quality Assessment

Methodological quality was assessed independently by four study team members for the 13 Tier A/B studies contributing to organism-specific synthesis and quantitative pooling (Table 2), using JBI critical appraisal tools appropriate to each study design, with disagreements resolved by discussion. Appraisal considered selection bias, comparability of groups, exposure and outcome measurement, confounding, follow-up completeness, statistical analysis, and appropriateness of conclusions [46]. All quality assessments were reviewed by the LPI for consistency and accuracy. Quality appraisal did not determine study eligibility; it informed interpretation of findings and certainty-of-evidence (GRADE) assessments only (Section 4.12).

### 4.10. Data Synthesis

A narrative synthesis was performed because included studies differed substantially in organism focus, intervention structure, comparator type, setting, and outcome measurement. Most interventions were multicomponent, and causal attribution to the pharmacist role specifically was not possible from the available study designs. Findings were summarized first by tier, then by Axis 1 organism/infection group, and then by Axis 2 intervention model. Clinical outcomes and process outcomes were interpreted separately to avoid overstating the clinical effect of process improvements. When studies reported both statistically significant and non-significant outcomes, both were presented to provide a balanced interpretation. Tier C studies were described as contextual evidence and were not used to make direct MDRO-specific effectiveness claims.

### 4.11. Statistical Analysis

Meta-analyses were conducted when at least two studies reported comparable dichotomous outcomes. Risk ratios (RRs) with 95% confidence intervals (CIs) were calculated directly from the raw event counts and denominators reported by each study. The pooled RR estimates are independent of, and should not be compared directly with, the odds ratios reported for some individual studies in Table 3, some of which are crude and some adjusted. Random-effects models were selected a priori given the anticipated clinical and methodological heterogeneity across stewardship interventions, infection types, and study designs. Statistical heterogeneity was assessed using Cochran’s Q test and quantified with the I^2^ statistic, with values of 25%, 50%, and 75% representing low, moderate, and high heterogeneity, respectively. Fixed-effect sensitivity analyses were performed to evaluate the robustness of pooled estimates. Analyses were conducted using Review Manager (RevMan), version 5.4.1. Only Tier A and Tier B studies were eligible for quantitative synthesis, as they provided MDRO-specific populations or extractable MDRO-specific outcome data; Tier C studies were excluded because their outcomes could not be separated from non-MDRO populations, introducing substantial indirectness, and limiting the validity of pooled effect estimates.

### 4.12. Certainty of the Evidence (GRADE Assessment)

The certainty of evidence for each quantitatively pooled outcome was assessed using the Grading of Recommendations Assessment, Development, and Evaluation (GRADE) approach, applied to the random-effects estimates described in Section 4.11, which served as the primary analysis for all pooled outcomes. Because all studies contributing to the meta-analysis were quasi-experimental or observational comparative designs rather than randomized controlled trials, each outcome began at an initial certainty rating of low, consistent with GRADE guidance for bodies of evidence composed of non-randomized studies. Ratings were subsequently adjusted by evaluating five domains: risk of bias, inconsistency, indirectness, imprecision, and publication bias. Risk of bias was informed by the JBI critical appraisal ratings described in Section 4.9, with particular attention to confounding, lack of randomization, and comparability of groups. Inconsistency was judged from the magnitude of the I^2^ statistic and the degree of overlap of confidence intervals across contributing studies, with I^2^ values of 50% or greater considered a serious concern and values approaching or exceeding 75% considered a very serious concern. Indirectness was judged by the concordance of study populations, interventions, comparators, and outcome definitions with the review question. Imprecision was judged from the width of the pooled 95% CI generated by the primary random-effects model, whether this CI crossed or reached the null value of 1.0, and the total number of events and participants contributing to the estimate. Publication bias could not be reliably assessed for any pooled outcome because fewer than ten studies contributed to any single analysis, which precluded funnel-plot inspection or Egger’s test; this should not be interpreted as evidence that publication bias was absent. Two reviewers independently assigned domain-level ratings for each outcome, with disagreements resolved by discussion; overall certainty was categorized as high, moderate, low, or very low for each outcome and summarized in the corresponding summary-of-findings table (Table 4).

### 4.13. Ethical Considerations

This review synthesized data from published studies and did not involve direct contact with human participants. Ethical approval was therefore not required. The review was conducted with attention to transparency, reproducibility, and accurate attribution of included studies.

## 5. Conclusions

This systematic review and meta-analysis found that multicomponent AMS interventions that involved pharmacists were consistently associated with improved process-of-care measures among patients with MDRO infections, including faster time to appropriate therapy, higher rates of antimicrobial de-escalation, reduced antimicrobial consumption, improved therapeutic drug monitoring, and better guideline adherence, across a wide range of organisms and intervention models. The exploratory meta-analysis showed statistically significant reductions in in-hospital mortality and 30-day readmission. In contrast, the reduction in 30-day mortality was not statistically significant, and no meaningful differences were found for recurrent infection or acute kidney injury. While these results indicate that pharmacist involvement may deliver clinically relevant benefits beyond improving stewardship processes, the certainty of evidence across all five pooled outcomes remained very low under GRADE, largely because the available studies were predominantly observational or quasi-experimental. These findings are consistent with, though insufficient on their own to firmly establish, an ongoing and potentially expanding role for pharmacists in multicomponent AMS for MDRO infections. Larger, prospective, and preferably randomized or cluster-randomized studies using harmonized MDRO-specific outcome definitions are needed before this role’s clinical and economic value can be confirmed with the certainty this evidence base otherwise deserves.

## Figures and Tables

**Figure 1:**
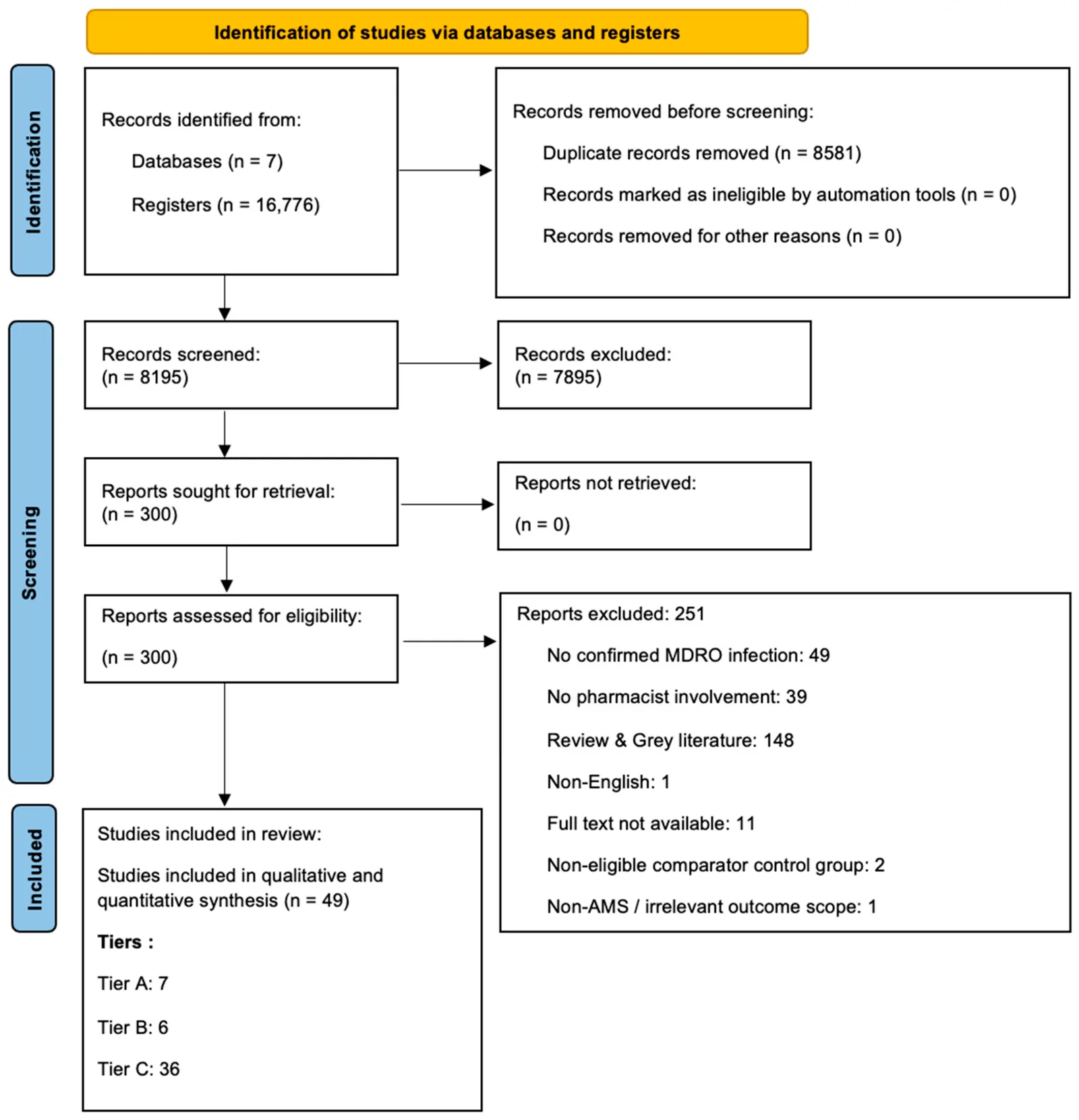
PRISMA Flowchart.

**Figure 2:**
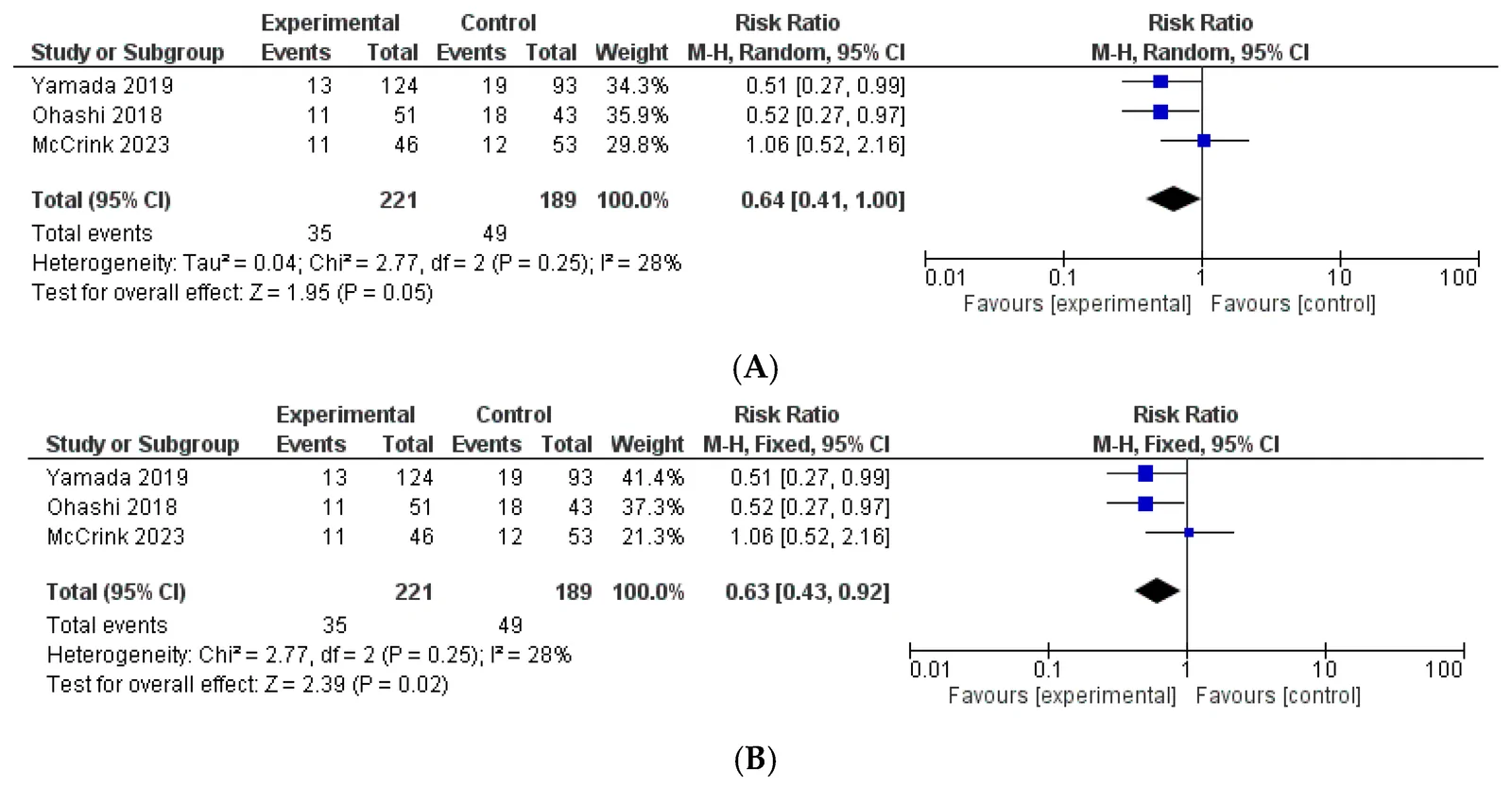
(**A**) 30-Day Mortality (Random-Effects Model). (**B**) 30-Day Mortality (Fixed-Effects Model—Sensitivity Analysis) [26,27,31].

**Figure 3:**
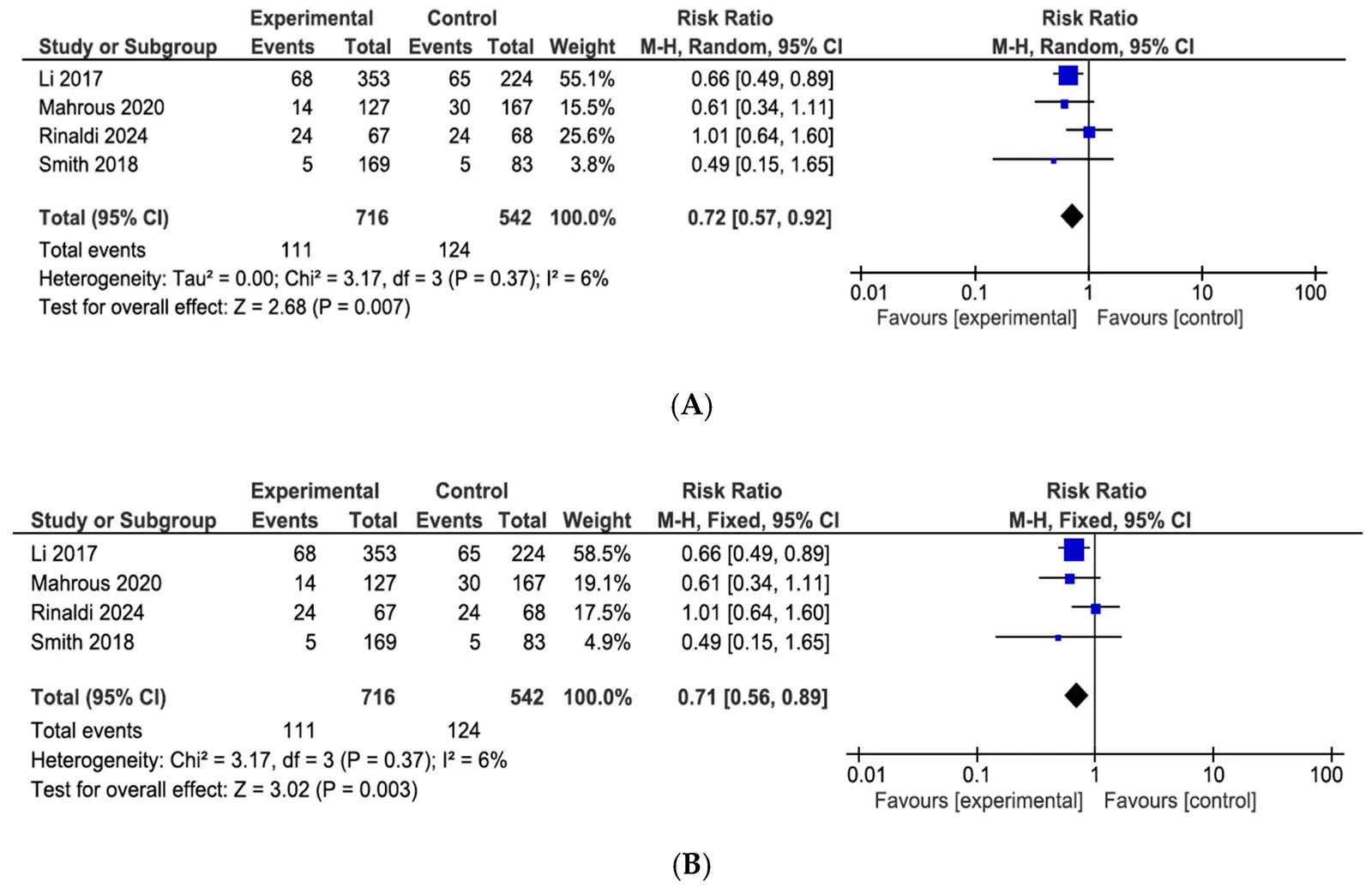
(**A**) In-Hospital Mortality (Random-Effects Model). (**B**) In-Hospital Mortality (Fixed-Effects Model—Sensitivity Analysis) [34,35,37,38].

**Figure 4:**
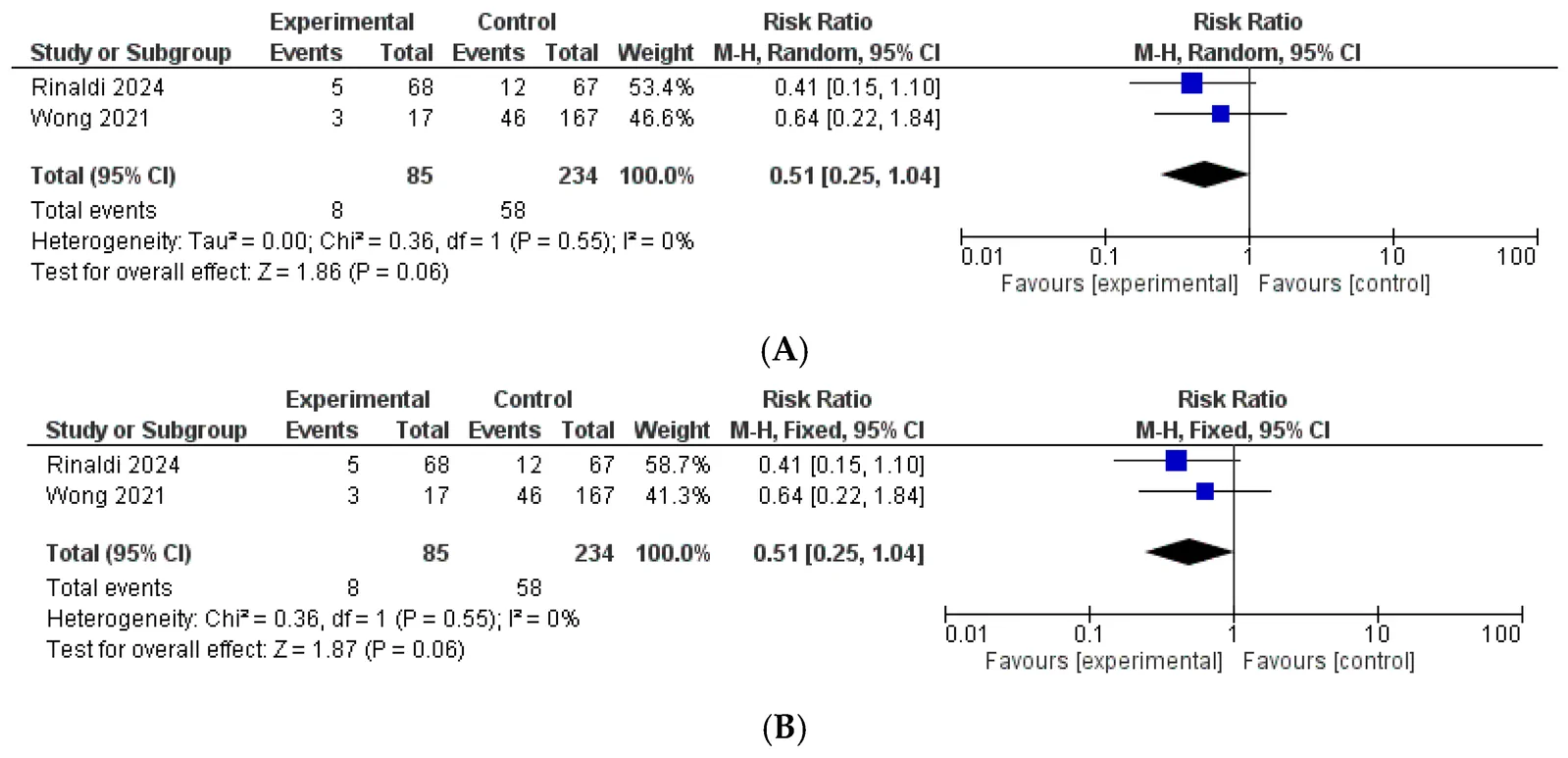
(**A**) Recurrent Infection (Random-Effects Model). (**B**) Recurrent Infection (Fixed-Effects Model—Sensitivity Analysis) [29,38].

**Figure 5:**
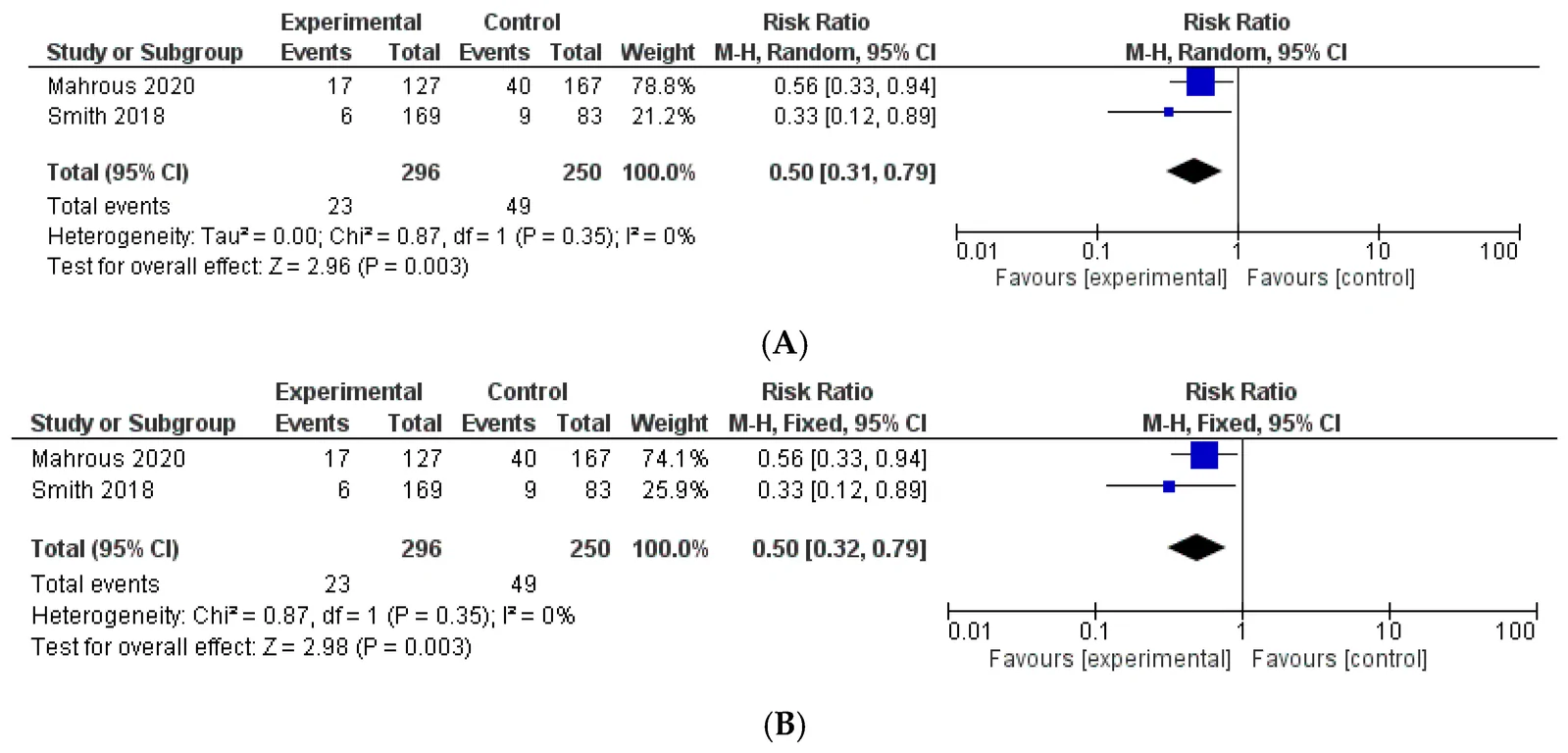
(**A**) Thirty-Day Readmission (Random-Effects Model). (**B**) Thirty-Day Readmission (Fixed-Effects Model—Sensitivity Analysis) [35,37].

**Figure 6:**
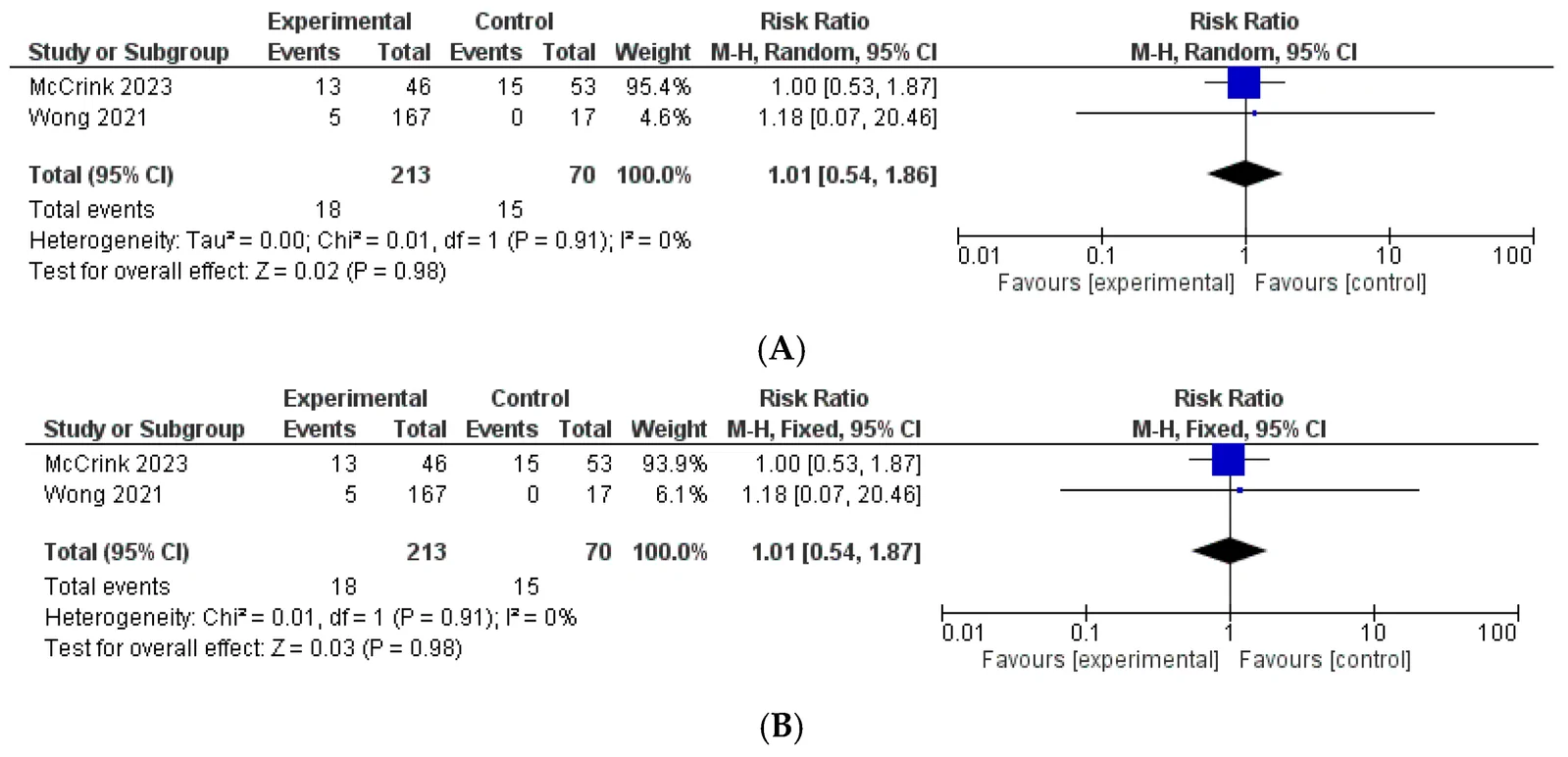
(**A**) Acute Kidney Injury (Random-Effects Model). (**B**) Acute Kidney Injury (Fixed-Effects Model—Sensitivity Analysis) [29,31].

**Table 1:** Characteristics of Included Studies.

No.	Study (Country)	Tier	Design	Setting	Sample Size (Groups) *	MDRO/Organism	Pharmacist Intervention	Main Clinical and Process Findings
1	Ohashi et al., 2018 (Japan) [26]	A	Quasi-experimental (pre–post)	Inpatient	N = 94 (pre = 43, post = 51)	MRSA bacteremia (100%)	ASP with pharmacist: early therapy optimization, blood culture follow-up, TDM, bundle-based management	30-day mortality decreased from 41.9% to 21.6%; hospital mortality decreased from 58.1% to 27.5%; appropriate therapy duration improved.
2	McCrink et al., 2023 (USA) [31]	A	Quasi-experimental (pre–post)	Hospital	N = 99 (control = 53, intervention = 46)	CRE & MDR *Pseudomonas aeruginosa* (100%)	Real-time diagnostic workflow with pharmacist dose-appropriateness assessment	Time to appropriate therapy improved significantly; mortality, bacteremia clearance, length of stay, and AKI were not significantly different.
3	Swingler et al., 2024 (USA) [32]	A	Quasi-experimental (pre–post)	Inpatient + ICU	N = 200	ESBL-producing Enterobacterales (100%)	Pharmacy-driven rapid bacteremia response program (rapid diagnostic workflow)	Mortality did not significantly change, but the workflow supported an earlier targeted response in ESBL bacteremia.
4	Wong et al., 2021 (USA) [29]	A	Retrospective quasi-experimental	Outpatient (OPAT)	Ertapenem n = 167 vs. aminoglycoside n = 17	ESBL-producing Enterobacterales (100%)	OPAT stewardship: switch ertapenem → aminoglycoside, optimize duration	Ertapenem DOT/1000 adjusted patient-days decreased by 19%; relapse and adverse events did not increase.
5	Yamada et al., 2019 (Japan) [27]	A	Retrospective quasi-experimental (pre–post)	Inpatient	Resistant-GNB subgroup: pre = 93, post = 124	Resistant Gram-negative (SPACE organisms)	AMS team: audit and feedback/antibiotic time-out	30-day mortality in resistant Gram-negative infections decreased; de-escalation acceptance was high; carbapenem DOT decreased.
6	Nakamura et al., 2021 (Japan) [28]	A	Quasi-experimental (pre–post)	Inpatient	N = 535 (pre = 288, post = 247)	MRSA, ESBL, CRE, VRE, MDR-*Acinetobacter*, MDR-*Pseudomonas* (100%; mixed MDRO cohort)	Bundle: post-prescription audit and feedback, de-escalation, dose optimization, guideline adherence, training, TDM	Recurrence and relapse decreased; de-escalation and TDM increased; mortality increased, likely reflecting case mix or severity.
7	Andrade et al., 2023 (USA) [30]	A	Quasi-experimental (pre–post)	Emergency department	N = 130 (pre = 60, post = 70)	Mixed MDROs (ESBL, MRSA, VRE)	Pharmacist-led culture follow-up with real-time MDRO notification	30-day emergency department revisits/readmissions decreased from 28.3% to 12.9%.
8	Smith et al., 2018 (USA) [35]	B	Quasi-experimental (propensity score–matched)	Inpatient	N = 258 (pre = 86, post = 172)	Mixed pathogens (42–43% MRSA)	Audit and feedback, guideline adherence, dose/duration optimization, bundle care	Time to appropriate therapy within 24 h improved; 30-day readmission decreased in the pharmacist-involved group; mortality difference not significant.
9	Li et al., 2017 (China) [34]	B	Prospective cohort (multicenter)	ICU	N = 577 (non-pharmacist = 224, pharmacist-driven = 353)	MRSA, *Acinetobacter baumannii*, *Klebsiella* spp., *Pseudomonas aeruginosa* (mixed MDR organisms)	Daily audits, culture/therapy review, de-escalation, multidisciplinary collaboration	Antimicrobial DOT decreased; mortality and length-of-stay findings varied by comparison and population.
10	Mahrous et al., 2020 (USA) [37]	B	Quasi-experimental (pre–post)	Inpatient + ICU	Mortality/readmission: intervention = 127, comparator = 167; other outcomes as reported: intervention = 148, comparator = 164	Mixed pathogens (majority MRSA)	Rapid diagnostic testing (Verigene) + pharmacist-directed ASP guidance	30-day readmission and in-hospital mortality both decreased; ICU length of stay decreased; antibiotic cost savings were reported.
11	Sadyrbaeva-Dolgova et al., 2019 (Spain) [36]	B	Quasi-experimental	Inpatient	N = 163 (de-escalation = 81, no de-escalation = 82)	Complicated UTI; ESBL/AmpC (majority)	Pharmacist-recommended carbapenem de-escalation	Mortality was lower in de-escalated patients; hospital length of stay was shorter; de-escalation increased significantly.
12	Knudsen & Andersen, 2014 (Denmark) [33]	B	Quasi-experimental (pre–post)	Inpatient + ICU	585-bed hospital; ESBL-KP mortality subgroup pre = 34, post = 29	ESBL/AmpC-producing *K. pneumoniae*	Bundled AMS and infection control (screening, contact precautions, restriction, guideline-directed prescribing)	Clinical cure improved; 30-day mortality decreased numerically but was not statistically significant.
13	Rinaldi et al., 2024 (Italy) [38]	B	Quasi-experimental (pre–post)	ICU	N = 135 (pre = 67, post = 68)	Gram-negative bloodstream infection (Enterobacterales, *Pseudomonas*)	ICU multidisciplinary management team with PK/PD-guided dose optimization	Meropenem exposure and dosing strategy improved; mortality and length of stay were similar.

* Sample sizes reflect the total study population for characteristics reported in this table; denominators for individual outcomes may differ (e.g., pre- vs. post-intervention cohort sizes, outcome-specific subgroups) and are reported separately, per outcome, in Table 3 in the study. Abbreviations: AKI, acute kidney injury; AMS, antimicrobial stewardship; ASP, antimicrobial stewardship program; DOT, days of therapy; ESBL, extended-spectrum beta-lactamase; GNB, Gram-negative bacteria; ICU, intensive care unit; MDRO, multidrug-resistant organism; MRSA, methicillin-resistant *Staphylococcus aureus*; OPAT, outpatient parenteral antimicrobial therapy; PK/PD, pharmacokinetic/pharmacodynamic; TDM, therapeutic drug monitoring.

**Table 2:** Quality Appraisal of Included Studies.

No.	Study (Country)	Tier *	Study Design	Overall Risk of Bias *
1	Ohashi et al., 2018 (Japan) [26]	A	Quasi-experimental (pre–post)	Low
2	McCrink et al., 2023 (USA) [31]	A	Quasi-experimental (pre–post)	Low
3	Swingler et al., 2024 (USA) [32]	A	Quasi-experimental (pre–post)	Moderate
4	Wong et al., 2021 (USA) [29]	A	Retrospective quasi-experimental	Low
5	Yamada et al., 2019 (Japan) [27]	A	Retrospective quasi-experimental (pre–post)	Moderate
6	Nakamura et al., 2021 (Japan) [28]	A	Quasi-experimental (pre–post)	Low
7	Andrade et al., 2023 (USA) [30]	A	Quasi-experimental (pre–post)	Low
8	Smith et al., 2018 (USA) [35]	B	Quasi-experimental (propensity score–matched)	Low
9	Li et al., 2017 (China) [34]	B	Prospective cohort (multicenter)	Moderate
10	Mahrous et al., 2020 (USA) [37]	B	Quasi-experimental (pre–post)	Low
11	Sadyrbaeva-Dolgova et al., 2019 (Spain) [36]	B	Quasi-experimental	Moderate
12	Knudsen & Andersen, 2014 (Denmark) [33]	B	Quasi-experimental (pre–post)	Moderate
13	Rinaldi et al., 2024 (Italy) [38]	B	Quasi-experimental (pre–post)	Low

* Tier A (MDRO-specific population); B, Tier B (mixed population with extractable MDRO-specific outcomes). Overall risk of bias was assessed using JBI critical appraisal tools appropriate to each study design (Section 4.9).

**Table 4:** Certainty of Evidence for Pooled Outcomes (GRADE).

Outcome	Studies (N)	Risk of Bias	Inconsistency	Indirectness	Imprecision	Publication Bias	Certainty (GRADE)	Effect, RR (95% CI)
**30-day mortality**	3 (410)	Serious ^1^	Not serious (I^2^ = 28%)	Serious ^4^	Serious ^3^	Could not be assessed ^2^	⊕◯◯◯ VERY LOW	0.64 (0.41–1.00)
**Recurrent infection**	2 (319)	Serious ^1^	Not serious (I^2^ = 0%)	Serious ^4^	Serious ^3^	Could not be assessed ^2^	⊕◯◯◯ VERY LOW	0.51 (0.25–1.04)
**Acute kidney injury**	2 (283)	Serious ^1^	Not serious (I^2^ = 0%)	Serious ^4^	Serious ^3^	Could not be assessed ^2^	⊕◯◯◯ VERY LOW	1.01 (0.54–1.87)
**In-hospital mortality**	4 (1264)	Serious ^1^	Not serious (I^2^ = 6%)	Serious ^4^	Not serious	Could not be assessed ^2^	⊕◯◯◯ VERY LOW	0.72 (0.57–0.92)
**30-day readmission**	2 (546)	Serious ^1^	Not serious (I^2^ = 0%)	Not Serious	Not serious	Could not be assessed ^2^	⊕◯◯◯ VERY LOW	0.50 (0.31–0.79)

GRADE certainty ratings: ⊕⊕⊕⊕ High; ⊕⊕⊕◯ Moderate; ⊕⊕◯◯ Low; ⊕◯◯◯ Very low. ^1^ Downgraded for risk of bias inherent to quasi-experimental/observational study designs lacking randomization and allocation concealment. ^2^ Fewer than 10 studies contributed to each outcome, precluding funnel-plot or Egger-test assessment; publication bias could not be assessed and should not be assumed absent. ^3^ 95% CI reaches or crosses the null value (1.0) with a limited total number of events. ^4^ Downgraded for indirectness: pooled studies differ in MDRO species, infection syndrome, setting, or intervention model.

## Data Availability

Data are available from the included published studies and extracted evidence tables.

## Supplementary Materials

The following supporting information can be downloaded at: https://www.mdpi.com/article/10.3390/antibiotics15100955/s1, File S1. Tier C Studies, cited references [24,47,48,49,50,51,52,53,54,55,56,57,58,59,60,61,62,63,64,65,66,67,68,69,70,71,72,73,74,75,76,77,78,79,80,81]; File S2. Risk of Bias Assessment of Individual Studies; File S3. Standardized Summary of Process Outcomes Across Tier A/B Studies; File S4. Search Strategies of Individual Databases.
