# Supplementary material for: Impact of Pharmacist-Involved Antimicrobial Stewardship Interventions on Clinical and Process Outcomes in Multidrug-Resistant Organism Infections: A Systematic Review and Meta-Analysis
Source: Antibiotics (Basel). 2026 Sep 25;15(10):955. doi: 10.3390/antibiotics15100955 (PMC13700879; doi:10.3390/antibiotics15100955)
Supplement: Supplementary file 1 [file antibiotics-15-00955-s001.zip › Supplementaty File S1.pdf]

Supplementaty File S1. Tier C Studies (Descriptive Reference)

Tier C studies enrolled mixed populations of MDRO and non-MDRO infections in which outcomes were not reported separately for MDRO-specific subgroups. These 36 studies were not eligible for the organism-specific or quantitative synthesis described in the main text and are summarized here for descriptive reference only.

No.	Study (Country)	Design	Setting	Sample Size	MDRO %	AMS Intervention(s)	Key Findings
1	Uda et al., 2022 (Japan) [1]	Quasi-experimental (3-period: baseline/intervention/post-intervention)	Inpatient	N = 1,161	NR	Structured antibiotic prescription review; prescriber education; monthly reporting of blood-culture sampling rates	De-escalation increased across antipseudomonal (54%→77%→73%, p=0.038) and anti-MRSA agents (58%→100%→100%, p=0.019); hospital LOS and antimicrobial consumption did not change significantly for any drug class; total antibiotic cost decreased from ¥649,165 (baseline) to ¥591,955 (intervention) and ¥599,986 (post-intervention).
2	Vogel et al., 2023 (USA, Miami) [2]	Single-center quasi-experimental	Inpatient	N = 94	58.8%	IV-to-oral switch (oral linezolid vs. IV vancomycin continuation)	No significant difference in hospital LOS (9 vs. 7 days, p=0.358) or antibiotic treatment duration (6 vs. 6 days, p=0.352); acute kidney injury (5% vs. 3%, p=0.635) and platelet count drop >50% (5% vs. 0%, p=0.392) did not differ significantly.
3	Mithawala & McGee, 2019 (USA, Lawrenceville, GA) [3]	Retrospective single-center pilot study	Inpatient	N = 41	17%	De-escalation	Meropenem de-escalation/discontinuation was recommended in 16 patients (80%) and accepted in 11 (68% acceptance); mean duration of meropenem therapy decreased by 2.5 days (intervention 5.6±1.8 vs. control 8.1±2.9 days, p=0.0175).
4	Gatechan et al., 2025 (Thailand) [4]	Retrospective study	ICU	N = 645	88.4% (MDR+XDR+PDR)	Audit and feedback; education and training	ICU mortality decreased (29.4%→18.6%, p=0.001); 30-day hospital mortality unchanged (39.0%→37.8%, p=0.772); ICU LOS decreased (median 7→5 days, p=0.005); DDD and DOT per 1,000 patient-days trended lower but were not statistically significant.
5	Arensman et al., 2020 (USA, Chicago) [5]	Retrospective study (3 sequential periods)	Mixed	N = 579	38%	Bundle care	Mortality did not differ significantly across periods (4.3%→2.3%→5.0%, p=0.6); source control improved (34%→45%→45%, p=0.04); hospital LOS varied without a consistent direction (10.5→8.85→12.0 days, p=0.01); ICU LOS and time to definitive therapy did not differ significantly; 30-day SAB-related readmission decreased (2.7%→0%→0%, p=0.08).

6	Ho and Islahudin, 2016 (Malaysia) [6]	Cross-sectional observational study	Mixed	N = 143	7.7%	Audit and feedback	Infection-related mortality was not significantly different (27.8%→20.3%, p=0.906); clinical cure improved significantly (43.0%→71.9%, p=0.001); hospital stay was not significantly shorter (15→13 days, p=0.150).
7	Trzebucki et al., 2024 (USA, Western Pennsylvania) [7]	Retrospective study	Inpatient	N = 628	NR	Rapid diagnostics	Duration of vancomycin among negative screens decreased with rapid PCR testing (3.6→3.1→2.0 days, p<0.001); same-day and next-day antibiotic discontinuation improved with PCR (p<0.02–<0.0001); acute kidney injury rate decreased (25.3%→14.8%→12.0%, p<0.001).
8	Ramadas et al., 2023 (Malaysia) [8]	Retrospective cohort study	Inpatient	N = 140	20%	De-escalation	Overall 30-day mortality was 32.9%; de-escalation was recommended in 47.9% of patients (80.6% acceptance) and escalation in 2.1% (66.7% acceptance); hospital LOS did not differ significantly by recommendation acceptance status (p=0.526).
9	Nakano et al., 2025 (Japan) [9]	Single-center retrospective quasi-experimental study	Inpatient	N = 567 (pre = 223, post = 344)	36%	Audit and feedback; TDM; dose/route/duration optimization; pre-authorization and formulary restriction; bundle care	30-day mortality did not differ significantly overall (crude HR 1.06, 95% CI 0.67–1.68; adjusted HR 0.92, 95% CI 0.56–1.51), but was significantly reduced in patients with Pitt bacteremia score ≥4 (crude HR 0.51, 95% CI 0.27–0.95; adjusted HR 0.52, 95% CI 0.28–0.99); carbapenem DOT decreased significantly (IRR 0.53, 95% CI 0.33–0.81, p=0.003).
10	Xin et al., 2025 (China) [10]	Retrospective cohort study	Mixed	N = 313	16.4%	Pharmacist consultations	14-day mortality was lower with pharmacist-recommendation adherence than non-adherence (5.75% vs. 17.31%, p<0.05); clinical response rate was higher with adherence (81.99% vs. 38.46%, p<0.05); adverse drug reactions were less frequent with adherence (4.21% vs. 13.46%, p<0.05); mean antimicrobial cost and total hospitalization cost were both lower with adherence (p<0.05).
11	Okada et al., 2022 (Japan) [11]	Quasi-experimental	Inpatient	N = 603	8.6%	Audit and feedback	30-day mortality did not differ significantly (6.5%→7.1%, p=0.79); DOT per 100 patient-days decreased significantly for carbapenems (26.9→13.5, p<0.01) and piperacillin-tazobactam (22.3→11.7, p<0.01); de-escalation increased significantly (32.4%→68.4%, p<0.01).

12	Jantarathaneewat et al., 2022 (Thailand) [12]	Quasi-experimental	Inpatient	N = 400 (intervention = 195, standard care = 205)	27.5%	Guideline and antibiogram adherence	All-cause mortality was numerically lower with ID pharmacist-led intervention than standard ASP (15.9% vs. 19.5%); infection resolution/cure was numerically higher (63.6% vs. 56.1%); hospital LOS was similar (median 20 vs. 18 days); carbapenem (p=0.04) and fosfomycin (p=0.01) consumption were significantly lower with the intervention.
13	Yamaguchi et al., 2022 (Japan) [13]	Quasi-experimental	Mixed	N = 1,400 (pre-PAF = 702, post-PAF = 698)	9.4% pre, 11.5% post	Audit and feedback (post-prescription audit and feedback)	All-cause mortality was similar (16.0%→15.0%); hospital LOS decreased (median 62→56 days); antimicrobial consumption decreased (median 8→7 days); de-escalation increased (58.0%→62.6%); adverse drug reactions decreased (12.5%→7.4%).
14	El Masri et al., 2022 (Lebanon, Beirut) [14]	Retrospective cohort	Mixed	N = 307 (Jan 2019 = 157, Jan 2020 = 150)	NR	NR	Carbapenem consumption (DDD/100 bed-days) was numerically higher in January 2020 than January 2019 (8.6); de-escalation was accepted in 100% of recommended cases in both periods (n=9 pre-ASP; n=20 post-ASP).
15	Kufel et al., 2021 (USA, New York) [15]	Quasi-experimental	Mixed	N = 201 (pre = 90, post = 111)	38.8%	Bundle care	90-day all-cause mortality decreased (17.8%→10.8%); hospital LOS decreased (median 14→13 days); time to appropriate therapy decreased (median 48→16 hours); 90-day readmission decreased (5.6%→2.7%).
16	Pham et al., 2021 (USA, Michigan) [16]	Retrospective quasi-experimental	Inpatient	N = 210 (pre-PCR = 138, PCR = 72)	2.8%	Rapid diagnostics	All-cause mortality was numerically lower with PCR testing but not statistically significant (13.0% vs. 6.9%, p=0.179); hospital LOS was similar (p=0.992); anti-MRSA therapy duration decreased significantly with PCR (2.5 vs. 1.4 days, p<0.001); IV-to-oral conversion increased with PCR (60.1% vs. 70.8%, p<0.001); 30-day readmission was lower with PCR (22.5% vs. 13.9%); AKI (12.5% vs. 8.9%, p=0.332) did not differ significantly.
17	Rainess et al., 2021 (USA) [17]	Quasi-experimental	Emergency department	N = 311 (pre = 220, post = 91)	5.47%	Dose/route/duration optimization	30-day emergency department return visits decreased (5.45%→2.20%).
18	Brotherton et al., 2020 (USA) [18]	Quasi-experimental	Hospital	N = 227 (pre = 111, post = 116)	47.6%	Clinical decision support systems	30-day all-cause mortality decreased (12.6%→6.0%); hospital LOS (median 17→18 days) and ICU LOS (median 6→7 days) did not meaningfully change; 30-day readmission decreased (32.0%→19.3%); 90-day readmission due to SAB complications decreased (14.3%→8.3%).

19	Alba Fernández et al., 2022 (Spain) [19]	Retrospective cohort pilot study	Hospital	N = 96 (audit accepted = 66, audit rejected = 30)	ESBL/AmpC 11.1% of 73.5% Gram-negative isolates	Audit and feedback	Mortality was higher among audits that were accepted than those rejected (16.4% vs. 3.3%); infection resolution/cure was numerically higher when audits were rejected (96.7%) than accepted (83.3%); hospital LOS was similar (15.0 vs. 17.0 days); meropenem consumption was lower when audits were accepted; 30-day infection-related readmission was numerically higher among accepted audits (66.7% vs. 50.0%).
20	Wang H et al., 2019 (China) [20]	Retrospective observational, interrupted time-series (hospital-wide, pre/during/post policy)	Mixed (hospital-wide, inpatient + outpatient)	Hospital-wide dataset, 2011–2016 (~376,600 inpatient antibiotic-utilization encounters; 350,699 inpatient isolates)	NR	Audit and feedback; dose/route/duration optimization; pre-authorization and formulary restriction; education and training; clinical decision support systems; guideline and antibiogram adherence	Inpatient antibiotic-use intensity decreased substantially (102.46→37.38 DDD/100 bed-days) with a significant downward trend during the intervention period; clinical outcomes (mortality, LOS, cure) were not reported.
21	Singh S et al., 2019 (India) [21]	Quasi-experimental (pre–post, single-center)	Mixed (inpatient + ICU, 13 units)	48,555 admissions (post-period); 1,020 patients received 1,326 restricted-antibiotic prescriptions	NR (high reported prevalence of Gram-negative resistance)	Post-prescription audit and feedback; guideline and antibiogram adherence; dose/route/duration optimization; education and training	All-cause mortality decreased (31.6→28.9 per 1,000 inpatients); hospital LOS decreased slightly (6.6→6.4 days); consumption decreased for polymyxins, colistin, and linezolid but increased for carbapenems (15.9→73.5); de-escalation compliance was 41%; mean monthly antibiotic cost decreased by 14.4% ($p=0.03$).
22	Kimura T et al., 2018 (Japan) [22]	Before-after quasi-experimental	Mixed (934-bed hospital, 2009–2016)	Not specified (hospital-wide)	NR	Audit and feedback; TDM; dose optimization; pre-authorization; guideline and antibiogram adherence; education and training; bundle care	30-day bacteremia mortality was unchanged ($p=0.57$), while overall hospital mortality decreased significantly ($p<0.001$); antipseudomonal DOT decreased (85.5→<60 per 1,000 patient-days, $p<0.01$) and carbapenem use decreased by 49%; TDM was implemented for vancomycin, teicoplanin, aminoglycosides, and voriconazole.
23	Beganovic M et al., 2017 (USA) [23]	Single-center pre–post quasi-experimental	Adult and pediatric inpatients (645-bed hospital)	N = 252 cultures (pre = 126, post = 126; 239 patients)	25% pre, 24% post	Audit and feedback; de-escalation; dose/route optimization; guideline adherence; education and training	All-cause mortality did not differ significantly (10.3%→12.2%); hospital LOS decreased significantly (15→9 days, $p=0.021$); ICU LOS was unchanged overall but decreased in the Gram-negative subgroup (4.3→1.2 days, $p=0.035$); time to optimal therapy decreased (75.2→43.1 hours, $p<0.001$); recurrence decreased non-significantly (3.5%→1.2%); overall hospital cost decreased ($p=0.010$; not antibiotic-specific).

24	Heyerly A et al., 2016 (USA) [24]	Single-center retrospective cohort (intervention vs. control)	Inpatient (396-bed hospital)	N = 297 (intervention = 107, control = 190)	NR	Audit and feedback; de-escalation; escalation; discontinuation; guideline adherence; rapid diagnostic result communication; automatic vancomycin dosing for MRSA signals	Hospital LOS was similar (11 days in both groups); time to optimal therapy improved with intervention (8.4 vs. 15.4 hours, p=0.0095); proportion achieving optimal therapy within 2 hours was higher with intervention (28% vs. 10.5%, p=0.0002).
25	Wong JR et al., 2012 (USA) [25]	Quasi-experimental pre–post (retrospective for pre-group)	Inpatient and ICU (1,200-bed academic center)	N = 53 (coagulase-negative staphylococcus contaminant cases; pre = 31, post = 22)	NR	Audit and feedback; rapid diagnostics; de-escalation; guideline adherence; antimicrobial duration optimization	Hospital LOS decreased from 14 to 9.5 days (not statistically significant); total antibiotic exposure time decreased (97.6→54.1 hours, p=0.011); time to discontinuation of unnecessary antibiotics improved (57.7→25.7 hours, p=0.005); recurrence after antibiotic discontinuation was 0% in both groups; antibiotic-related cost decreased (not statistically significant).
26	Thayer et al., 2024 (USA) [26]	Retrospective pre–post cohort study	Inpatient, large community teaching hospital	N = 725 (pre = 100, post = 625)	NR	Pharmacist-driven MRSA nasal PCR ordering protocol to guide vancomycin de-escalation	All-cause mortality did not differ significantly (27%→22%, p=0.33); hospital LOS decreased significantly (13.2→11 days, p<0.05); vancomycin DOT decreased (3.4→2.0 days, p<0.05); 30-day readmission decreased significantly (23%→14%, p=0.02).
27	Berger et al., 2021 (USA) [27]	Retrospective pre–post cohort study	Inpatient, 185-bed community hospital	N = 193 (pre = 83, post = 110)	MRSA 42% (36/83 pre, 45/110 post)	Bundle care (appropriate IV therapy, duration, repeat cultures, echocardiography, ID consult, source control) with pharmacist notification and real-time recommendations	All-cause mortality decreased significantly (15.7%→3.6%, p=0.0033); time to defervescence was similar (p=0.49); hospital LOS was unchanged (10.2→10.6 days, p=0.72); time to appropriate therapy decreased significantly (24.5→11.7 hours, p=0.0011).
28	Huffman V et al., 2020 (USA) [28]	Quasi-experimental pre–post	Mixed general wards (502-bed hospital)	N = 62 (pre = 20, post = 42)	NR	Bundle (MRSA PCR nasal swab-based de-escalation protocol with prescriber education and workflow algorithms)	In-hospital mortality did not differ significantly (10% vs. 9.5%, p=0.953); hospital LOS did not differ significantly (p=0.407); antimicrobial duration decreased significantly (90.7±54.4→59.7±50.4 hours, p=0.039); de-escalation increased significantly with negative PCR results; 30-day readmission did not differ significantly (p=0.432); median antibiotic cost decreased significantly (p<0.01).
29	Phillips CJ et al., 2015 (Australia, South Australia) [29]	Quasi-experimental observational pre–post (no control group)	Mixed, metropolitan teaching hospital	N = 99 (pre = 53, post = 46)	NR	Education (structured teaching sessions, guideline cards, embedded dosing calculator, TDM-timing focus)	Infection resolution/cure improved (84.5%→95.8%); hospital LOS decreased non-significantly (20→16 days, p=0.13); vancomycin therapy duration decreased (10→6 days); in-target trough levels improved by ~10% and toxic levels decreased by 10%

							(p=0.05); no significant difference in nephrotoxicity.
30	Pasquale TR et al., 2014 (USA) [30]	Retrospective observational (pre–post with historical control)	Mixed, 577-bed community teaching hospital	N = 1,211 (ASP intervention = 62; historical control = 1,149)	~30% (50% of 61.5% S. aureus isolates were MRSA)	Comprehensive pharmacist-led ASP (prospective audit and feedback, dose optimization, de-escalation, regimen change, ID consultation, prescriber education; 95% recommendation acceptance)	Hospital LOS decreased significantly (6.2→4.4 days, p<0.001); de-escalation to narrower-spectrum therapy occurred in 37% of patients; 30-day all-cause readmission decreased significantly vs. historical data (16.7%→6.5%, p=0.05); 30-day infection-specific readmission decreased non-significantly (6.2%→3.3%, p=0.48).
31	Okumura LM et al., 2015 (Brazil) [31]	Retrospective historical cohort (bundled ASP vs. conventional ASP)	Mixed, adult wards and ICUs (550-bed hospital)	N = 491 (bundled ASP = 191, conventional ASP = 300)	Not formally reported; resistant isolates more frequent in the bundled-ASP arm	Bundled ASP: daily pharmacist screening of prescriptions and laboratory data, in-person discussion with infectious diseases physicians and microbiologists, prescriber education, continuous follow-up to clinical resolution/discharge	30-day all-cause mortality was significantly lower with bundled ASP (27.2% vs. 38.0%, p<0.01; RR 0.72, 95% CI 0.54–0.94; NNT ≈9.3); daily antimicrobial consumption was significantly lower with bundled ASP (p<0.01); among accepted interventions, de-escalation accounted for 8%, treatment suspension for 28%, and escalation for 6%.
32	Bispo da Silva SCS et al., 2024 (Brazil) [32]	Retrospective pediatric cohort with pre–post comparison and multivariate analysis	Mixed pediatric ICU and wards	N = 120 (pre 2014–2016 = 44, intervention 2017–2021 = 76)	17% MRSA (of S. aureus bacteremia cases)	Pharmacist-led S. aureus bacteremia care bundle (follow-up blood cultures, early source control, echocardiography when complicated, de-escalation to oxacillin for MSSA, vancomycin TDM for MRSA, appropriate treatment duration)	The intervention period was independently associated with lower odds of therapeutic failure (composite of infection-related mortality and 90-day reinfection) (OR 0.060, 95% CI 0.005–0.806, p=0.034); hospital and ICU LOS trended shorter (not statistically significant); de-escalation to oxacillin for MSSA improved (61%→78%) and was independently protective against therapeutic failure (OR 0.041, 95% CI 0.002–0.941, p=0.046); vancomycin TDM adherence for MRSA reached 100% where applicable.
33	Yu et al., 2023 (China) [33]	Quasi-experimental before–after cohort study	Neurosurgical ICU	N = 1,039 (pre = 541, post = 498)	18.5%	Prospective audit and feedback; daily ward rounds; guideline education; dose/route optimization; de-escalation	All-cause ICU mortality decreased non-significantly (26.6%→22.4%, p=0.6); de-escalation rate decreased significantly (39.66%→20%, p<0.001); readmission for the same cause increased non-significantly (0%→3.4%, p=0.15); polymyxin and tigecycline DOT decreased non-significantly; adverse drug reactions were numerically lower post-intervention (12.07% vs. 20%, p=0.264).
34	Abdelaal et al., 2025 (Egypt) [34]	Prospective quasi-experimental pre–post	Neonatal ICU	N = 1,200 (pre = 603, post = 597)	High MDR/XDR prevalence reported (most	Protocol modification; prospective audit and feedback; dose optimization; empiric and culture-guided therapy optimization;	14-day and 28-day mortality among late-onset sepsis cases decreased significantly (14-day: 33.3%→20.9%; 28-day: 46.4%→32.1%; both p≤0.03); mortality was unchanged for early-onset and hospital-acquired sepsis;

					infections MDRO)	antifungal prophylaxis; education; microbiology workflow alignment	hospital LOS did not change significantly across sepsis categories; length of therapy decreased significantly for ampicillin/sulbactam, vancomycin, and cefotaxime but increased for amikacin and piperacillin/tazobactam; readmission rates were low and not significantly different between periods.
35	Okada et al., 2016 (Japan) [35]	Retrospective observational pre–post	Hematology ward	N = 145 (pre = 71, post = 74)	100%	Pharmacist-led TDM within a pre-authorization protocol; infection control team collaboration; dose prediction and optimization; regimen recommendations; follow-up TDM	Defervescence by day 3 in lymphoma patients improved (14%→53%); hospital LOS decreased overall (70→47.5 days) and in lymphoma patients (61→35 days) but increased in leukemia patients (70→135 days); antimicrobial utilization density increased for vancomycin and teicoplanin but decreased for arbekacin; TDM target attainment improved (55%→74%); acute kidney injury decreased non-significantly (7%→4%).
36	Dillon et al., 2024 (USA) [36]	Retrospective pre–post intervention study	Inpatient, large teaching hospital	N = 110 (pre = 61, post = 49)	22.2%	De-escalation; audit and feedback; rapid diagnostics (culture-based nasal MRSA screening); guideline adherence; education; targeted pharmacist-led review	MRSA nasal swab ordering increased significantly (36.1%→83.7%, $p<0.0001$); vancomycin de-escalation after a negative swab increased significantly (19.7%→61.2%, $p<0.0001$); identified inappropriate antibiotic continuation increased (1.6%→12.2%, reflecting improved detection rather than worse practice); overall days of therapy did not change significantly (3.85→3.53 days, $p=0.32$); estimated annual drug cost savings of ~$2,716 if therapy were reduced by one day.

Abbreviations: AKI, acute kidney injury; ASP, antimicrobial stewardship program; DDD, defined daily dose; DOT, days of therapy; ID, infectious diseases; IV, intravenous; LOS, length of stay; MDRO, multidrug-resistant organism; MRSA, methicillin-resistant Staphylococcus aureus; MSSA, methicillin-susceptible Staphylococcus aureus; NNT, number needed to treat; NR, not reported; PBPM, protocol-based pharmacotherapy management; PCR, polymerase chain reaction; SAB, Staphylococcus aureus bacteremia; TDM, therapeutic drug monitoring.

References

1. Uda, A.; Ebisawa, K.; Sakon, H.; Kusuki, M.; Izuta, R.; Yahata, M.; Yano, I.; Miyara, T. Sustained Improvements in Antimicrobial Therapy and Clinical Outcomes following a Pharmacist-Led Antimicrobial Stewardship Intervention: Uncontrolled Before–After Study. *J. Clin. Med.* **2022**, *11*, 566. https://doi.org/10.3390/jcm11030566.
2. Vogel, M.S.; Cua, J.; Dittmar, E.; Gauthier, T.; Williams, K.N. Assessment of a Pharmacist-Led Antibiotic Time-Out for Transition of IV Vancomycin to Oral Linezolid. *Nurs. Health Sci. Res. J.* **2023**, *6*, 25–33. https://doi.org/10.55481/2578-3750.1172.
3. Mithawala, P.; McGee, E.-A. Evaluation of the Impact of Antimicrobial Stewardship Program (ASP) on Unrestricted Use of Meropenem at an Academic Medical Center. *J. Contemp. Pharm. Pract.* **2019**, *66*, 29–33. https://doi.org/10.37901/jcphp17-00030.

4. Gatechan, T.; Nakaranurack, C.; Plongla, R.; Chuenjit, T.; Gross, A.E. The Impact of Pharmacist-Led Education and Prospective Audit and Feedback on Antibiotic Dose Optimization within Medical Intensive Care Units in Thailand: A Retrospective Study. *J. Pharm. Policy Pract.* **2025**, *18*, 2467456. https://doi.org/10.1080/20523211.2025.2467456.
5. Arensman, K.; Dela-Pena, J.; Miller, J.L.; LaChance, E.; Beganovic, M.; Anderson, M.; Rivelli, A.; Wieczorkiewicz, S.M. Impact of Mandatory Infectious Diseases Consultation and Real-Time Antimicrobial Stewardship Pharmacist Intervention on *Staphylococcus aureus* Bacteremia Bundle Adherence. *Open Forum Infect. Dis.* **2020**, *7*, ofaa184. https://doi.org/10.1093/ofid/ofaa184.
6. Ho, L.K.; Islahudin, F. Carbepenem Stewardship and the Impact on Appropriate Use and Clinical Outcomes. *Res. J. Pharm. Technol.* **2016**, *9*, 699–704. https://doi.org/10.5958/0974-360X.2016.00131.1.
7. Trzebucki, A.M.; Davis, M.R.; McCreary, E.K.; Cuccaro, S.; Bariola, J.R.; Khadem, T. Impact of a Pharmacy-Driven MRSA Nares Screening Protocol on Vancomycin Discontinuation in a Tele-Antimicrobial Stewardship Model. *Antimicrob. Steward. Healthc. Epidemiol.* **2024**, *4*, e56. https://doi.org/10.1017/ash.2024.43.
8. Ramadas, A.; Ambaras Khan, R.; Khalid, K.E.; Leong, C.L.; Makmor-Bakry, M. Clinical Impact of Multidisciplinary Carbapenem Stewardship Interventions: A Retrospective Cohort Study. *J. Pharm. Policy Pract.* **2023**, *16*, 94. https://doi.org/10.1186/s40545-023-00599-0.
9. Nakano, Y.; Hirai, T.; Murata, M.; Yasukochi, H.; Ura, K.; Sueyasu, Y.; Shimono, N.; Hasuwa, H. Impact of Pharmacist-Driven Antimicrobial Stewardship Interventions in a Secondary Care Facility in Japan: A Pragmatic Quasi-Experimental Study. *J. Infect. Chemother.* **2025**, *31*, 102503. https://doi.org/10.1016/j.jiac.2024.08.018.
10. Xin, C.; Zheng, B.; Zhao, Q.; Yang, W.; Feng, P.; Tu, Y. Impact of Pharmacist-Led Anti-Infective Consultations on Therapeutic Outcomes: A Retrospective Cohort Study in a Tertiary Hospital. *Infect. Drug Resist.* **2025**, *18*, 3735–3744. https://doi.org/10.2147/IDR.S534678.
11. Okada, N.; Azuma, M.; Tsujinaka, K.; Abe, A.; Takahashi, M.; Yano, Y.; Sato, M.; Shibata, T.; Goda, M.; Ishizawa, K. Clinical Impact of a Pharmacist-Driven Prospective Audit with Intervention and Feedback on the Treatment of Patients with Bloodstream Infection. *Antibiotics* **2022**, *11*, 1144. https://doi.org/10.3390/antibiotics11091144.
12. Jantarathaneewat, K.; Montakantikul, P.; Weber, D.J.; Nanthapisal, S.; Rutjanawech, S.; Apisarnthanarak, A. Impact of an Infectious Diseases Pharmacist-Led Intervention on Antimicrobial Stewardship Program Guideline Adherence at a Thai Medical Center. *Am. J. Health-Syst. Pharm.* **2022**, *79*, 1266–1272. https://doi.org/10.1093/ajhp/zxac107.
13. Yamaguchi, R.; Yamamoto, T.; Okamoto, K.; Tatsuno, K.; Ikeda, M.; Tanaka, T.; Wakabayashi, Y.; Sato, T.; Okugawa, S.; Moriya, K.; et al. Prospective Audit and Feedback Implementation by a Multidisciplinary Antimicrobial Stewardship Team Shortens the Time to De-Escalation of Anti-MRSA Agents. *PLoS ONE* **2022**, *17*, e0271812. https://doi.org/10.1371/journal.pone.0271812.
14. El Masri, M.; Haddad, N.; Saad, T.; Rizk, N.A.; Zakhour, R.; Kanj, S.S.; Zeenny, R.M. Evaluation of Carbapenem Use Before and After Implementation of an Antimicrobial Stewardship-Led Carbapenem-Sparing Strategy in a Lebanese Tertiary Hospital: A Retrospective Study. *Front. Cell. Infect. Microbiol.* **2022**, *12*, 729491. https://doi.org/10.3389/fcimb.2022.729491.
15. Kufel, W.D.; Mastro, K.A.; Steele, J.M.; Wang, D.; Riddell, S.W.; Paolino, K.M.; Thomas, S.J. Impact of a Pharmacist-Facilitated, Evidence-Based Bundle Initiative on *Staphylococcus aureus* Bacteremia Management. *Diagn. Microbiol. Infect. Dis.* **2021**, *101*, 115535. https://doi.org/10.1016/j.diagmicrobio.2021.115535.
16. Pham, S.N.; Sturm, A.C.; Jacoby, J.S.; Egwuatu, N.E.; Dumkow, L.E. Impact of a Pharmacist-Driven MRSA Nasal PCR Protocol on Pneumonia Therapy. *Hosp. Pharm.* **2021**, *56*, 221–227. https://doi.org/10.1177/0018578719888906.
17. Rainess, R.A.; Patel, V.V.; Cavanaugh, J.B.; Hill, J. Evaluating the Addition of a Clinical Pharmacist Service to a Midlevel Provider-Driven Culture Follow-Up Program in a Community Emergency Department. *J. Pharm. Technol.* **2021**, *37*, 140–146. https://doi.org/10.1177/87551225211000363.
18. Brotherton, A.L.; Rab, S.; Kandiah, S.; Kriengkauykiat, J.; Wong, J.R. The Impact of an Automated Antibiotic Stewardship Intervention for the Management of *Staphylococcus aureus* Bacteraemia Utilizing the Electronic Health Record. *J. Antimicrob. Chemother.* **2020**, *75*, 1054–1060. https://doi.org/10.1093/jac/dkz518.
19. Alba Fernández, J.; Del Pozo, J.L.; Leiva, J.; Fernández-Alonso, M.; Aquerreta, I.; Aldaz, A.; Blanco, A.; Yuste, J.R. Impact of the Acceptance of the Recommendations Made by a Meropenem Stewardship Program in a University Hospital: A Pilot Study. *Antibiotics* **2022**, *11*, 330. https://doi.org/10.3390/antibiotics11030330.
20. Wang, H.; Wang, H.; Yu, X.; Zhou, H.; Li, B.; Chen, G.; Ye, Z.; Wang, Y.; Cui, X.; Zheng, Y.; et al. Impact of Antimicrobial Stewardship Managed by Clinical Pharmacists on Antibiotic Use and Drug Resistance in a Chinese Hospital, 2010–2016: A Retrospective Observational Study. *BMJ Open* **2019**, *9*, e026072. https://doi.org/10.1136/bmjopen-2018-026072.
21. Singh, S.; Menon, V.P.; Mohamed, Z.U.; Kumar, V.A.; Nampoothiri, V.; Sudhir, S.; Moni, M.; Dipu, T.S.; Dutt, A.; Edathadathil, F.; et al. Implementation and Impact of an Antimicrobial Stewardship Program at a Tertiary Care Center in South India. *Open Forum Infect. Dis.* **2019**, *6*, ofy290. https://doi.org/10.1093/ofid/ofy290.

22. Kimura, T.; Uda, A.; Sakaue, T.; Yamashita, K.; Nishioka, T.; Nishimura, S.; Ebisawa, K.; Nagata, M.; Ohji, G.; Nakamura, T.; et al. Long-Term Efficacy of Comprehensive Multidisciplinary Antibiotic Stewardship Programs Centered on Weekly Prospective Audit and Feedback. *Infection* **2018**, *46*, 215–224. https://doi.org/10.1007/s15010-017-1099-8.
23. Beganovic, M.; Costello, M.; Wieczorkiewicz, S.M. Effect of Matrix-Assisted Laser Desorption Ionization–Time of Flight Mass Spectrometry (MALDI-TOF MS) Alone versus MALDI-TOF MS Combined with Real-Time Antimicrobial Stewardship Interventions on Time to Optimal Antimicrobial Therapy in Patients with Positive Blood Cultures. *J. Clin. Microbiol.* **2017**, *55*, 1437–1445. https://doi.org/10.1128/JCM.02245-16.
24. Heyerly, A.; Jones, R.; Bokhart, G.; Shoaff, M.; Fisher, D. Implementation of a Pharmacist-Directed Antimicrobial Stewardship Protocol Utilizing Rapid Diagnostic Testing. *Hosp. Pharm.* **2016**, *51*, 815–822. https://doi.org/10.1310/hpj5110-815.
25. Wong, J.R.; Bauer, K.A.; Mangino, J.E.; Goff, D.A. Antimicrobial Stewardship Pharmacist Interventions for Coagulase-Negative Staphylococci Positive Blood Cultures Using Rapid Polymerase Chain Reaction. *Ann. Pharmacother.* **2012**, *46*, 1484–1490. https://doi.org/10.1345/aph.1R439.
26. Thayer, B.; Edwards, J.D.; Belk, M.G.; Durham, S.H. Evaluation of a Pharmacist-Led Methicillin-Resistant *Staphylococcus aureus* Nasal PCR Testing Protocol. *Antibiotics* **2024**, *13*, 1195. https://doi.org/10.3390/antibiotics13121195.
27. Berger, N.J.; Wright, M.E.; Pouliot, J.D.; Green, M.W.; Armstrong, D.K. The Impact of a Pharmacist-Driven *Staphylococcus aureus* Bacteremia Initiative in a Community Hospital: A Retrospective Cohort Analysis. *Pharmacy* **2021**, *9*, 191. https://doi.org/10.3390/pharmacy9040191.
28. Huffman, V.; Andrade, D.C.; Ham, J.; Brown, K.; Melnitsky, L.; Lopez Cohen, A.; Parmar, J. Impact of Nasal Swabs on Empiric Treatment of Respiratory Tract Infections (INSERT-RTI). *Pharmacy* **2020**, *8*, 101. https://doi.org/10.3390/pharmacy8020101.
29. Phillips, C.J.; Gordon, D.L. Pharmacist-Led Implementation of a Vancomycin Guideline across Medical and Surgical Units: Impact on Clinical Behavior and Therapeutic Drug Monitoring Outcomes. *Integr. Pharm. Res. Pract.* **2015**, *4*, 145–152. https://doi.org/10.2147/IPRP.S92850.
30. Pasquale, T.R.; Trienski, T.L.; Olexia, D.E.; Myers, J.P.; Tan, M.J.; Leung, A.K.; Poblete, J.E.; File, T.M., Jr. Impact of an Antimicrobial Stewardship Program on Patients with Acute Bacterial Skin and Skin Structure Infections. *Am. J. Health-Syst. Pharm.* **2014**, *71*, 1136–1139. https://doi.org/10.2146/ajhp130677.
31. Okumura, L.M.; da Silva, M.M.G.; Veroneze, I. Effects of a Bundled Antimicrobial Stewardship Program on Mortality: A Cohort Study. *Braz. J. Infect. Dis.* **2015**, *19*, 246–252. https://doi.org/10.1016/j.bjid.2015.02.005.
32. Schenidt Bispo da Silva, S.C.; Fachi, M.M.; Ricieri, M.C.; de Araújo Motta, F. Pharmacist-Led Antimicrobial Stewardship Program in the Treatment of *Staphylococcus aureus* Bacteraemia in Paediatric Patients: A Multivariate Analysis. *Infect. Prev. Pract.* **2024**, *6*, 100419. https://doi.org/10.1016/j.infpip.2024.100419.
33. Yu, J.; Liu, Y.; Qu, R.; Wang, Z.; Zhao, Y.; Zhao, Y.; Zhou, C. Evaluation of a Clinical Pharmacist-Led Antimicrobial Stewardship Program in a Neurosurgical Intensive Care Unit: A Pre- and Post-Intervention Cohort Study. *Front. Pharmacol.* **2023**, *14*, 1263618. https://doi.org/10.3389/fphar.2023.1263618.
34. Abdelaal, N.H.; Abdel Aziz, N.H.R.; Abdelaziz, A.M.; Khalil, S.B.H.; Abdel-Latif, M.M.M. Clinical Impact of an Antibiotic Stewardship Program in a Neonatal Intensive Care Unit at a Tertiary Care Hospital: A Prospective Quasi-Experimental Clinical Study. *J. Pharm. Health Care Sci.* **2025**, *11*, 81. https://doi.org/10.1186/s40780-024-00404-3.
35. Okada, N.; Fushitani, S.; Azuma, M.; Nakamura, S.; Nakamura, T.; Teraoka, K.; Watanabe, H.; Abe, M.; Kawazoe, K.; Ishizawa, K. Clinical Evaluation of Pharmacist Interventions in Patients Treated with Anti-Methicillin-Resistant *Staphylococcus aureus* Agents in a Hematological Ward. *Biol. Pharm. Bull.* **2016**, *39*, 295–300. https://doi.org/10.1248/bpb.b15-00774.
36. Dillon, J.; Vita, D.; Abrantes-Figueiredo, J.; Wiskirchen, D. Impact of Stewardship Pharmacist Driven MRSA Nasal Surveillance and De-Escalation of Anti-MRSA Therapy (STEW PHARM MRSA NASAL SURVEILLANCE). *Antimicrob. Steward. Healthc. Epidemiol.* **2024**, *4*, e216. https://doi.org/10.1017/ash.2024.443.
