# Supplementary material for: Impact of Pharmacist-Involved Antimicrobial Stewardship Interventions on Clinical and Process Outcomes in Multidrug-Resistant Organism Infections: A Systematic Review and Meta-Analysis
Source: Antibiotics (Basel). 2026 Sep 25;15(10):955. doi: 10.3390/antibiotics15100955 (PMC13700879; doi:10.3390/antibiotics15100955)
Supplement: Supplementary file 1 [file antibiotics-15-00955-s001.zip › Supplementary File S2.pdf]

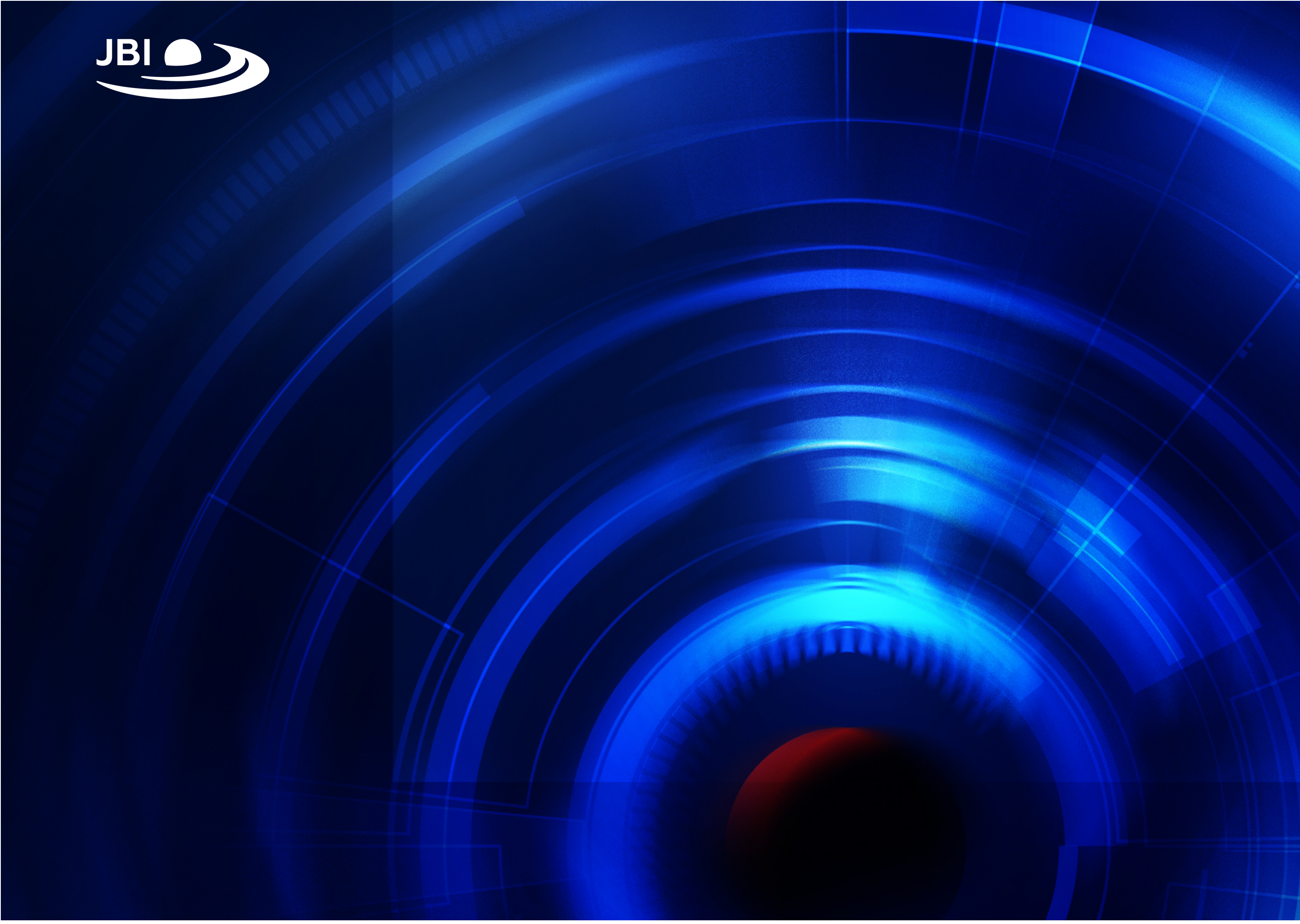

JBI CRITICAL APPRAISAL TOOL

JBI CHECKLIST FOR QUASI-EXPERIMENTAL STUDIES

2023

jbi.global

CRICOS Provider Number 00123M

INTRODUCTION

JBI is a global organization promoting and supporting evidence-based decisions that improve health and health service delivery. JBI offers a unique range of solutions to access, appraise and apply the best available evidence, servicing over 90 countries. Working with 80+ universities, hospitals and NGOs from across the globe through the JBI Collaboration, JBI is a recognized global leader in evidence-based health care.

JBI Systematic Reviews

The core of evidence synthesis is the systematic review of literature of a particular intervention, condition or issue. The systematic review is essentially an analysis of the available evidence and a judgment of the effectiveness or otherwise of a practice, involving a series of complex steps. JBI takes a particular view on what counts as evidence and the methods utilized to synthesize those different types of evidence. In line with this broader view of evidence, JBI has developed theories, methodologies and rigorous processes for the critical appraisal and synthesis of these diverse forms of evidence in order to aid in clinical decision-making in health care. Guidance now exists for conducting reviews of effectiveness research, qualitative research, prevalence/incidence, etiology/risk, economic evaluations, text/opinion, diagnostic test accuracy, mixed-methods, umbrella reviews and scoping reviews. Further information regarding JBI systematic reviews can be found in the JBI Manual for Evidence Synthesis.

JBI Critical Appraisal Tools

All systematic reviews incorporate a process of critique or appraisal of the research evidence. The purpose of this appraisal for quantitative evidence is to determine the extent to which a study has addressed the possibility of bias in its design, conduct and analysis. All papers selected for inclusion in the systematic review (that is – those that meet the inclusion criteria described in the protocol) need to be subjected to rigorous appraisal by two critical appraisers. The results of this appraisal can then be used to inform synthesis and interpretation of the results of the study. Although designed for use in systematic reviews, JBI critical appraisal tools can also be used when creating Critically Appraised Topics, in journal clubs and as an educational tool.

How were these tools developed?

JBI critical appraisal tools have been developed by JBI and collaborators. The particular iteration of this tool was developed by the JBI Effectiveness Methodology Group following oversight by the JBI Scientific Committee.

Like the previous versions of these tools, this version presents signaling questions to prompt reviewers to identify whether certain safeguards of bias have been met, in the primary literature under review. However, unlike previous iterations of this tool, this version has separated questions into whether they provide an answer relating to internal, external or statistical conclusion validity. For questions related to internal validity, these have been further separated to identify what domain of bias they are referring. Finally, this tool has also been structured to facilitate judgments related to bias at different levels (e.g. bias at the outcome level or bias at the result level) where appropriate.

These tools have been approved following extensive peer review by the JBI Scientific Committee.

How to cite

Please use the following when citing this tool: Barker TH, Habibi N, Aromataris E, Stone JC, Leonardi-Bee J, Sears K, et al. The revised JBI critical appraisal tool for the assessment of risk of bias quasi-experimental studies. JBI Evid Synth. 2024;22(3):378-88.

Internal Validity		Choice - Comments/Justification	Yes	No	Unclear	N/A
**Bias related to temporal precedence**						
1	**Is it clear in the study what is the "cause" and what is the "effect" (i.e. there is no confusion about which variable comes first)?**	The study explicitly defines the cause (implementation of a pharmacist-led antimicrobial stewardship (AS) intervention with MRSA-bacteremia treatment bundle) and the effect (changes in bundle adherence, mortality, and clinical outcomes)	☒	☐	☐	☐
**Bias related to selection and allocation**						
2	**Was there a control group?**	The pre-intervention group (before the AMS program implementation) served as a historical control, allowing assessment of the intervention's effect.	☒	☐	☐	☐
**Bias related to confounding factors**						
3	**Were participants included in any comparisons similar?**	The pre- and post-intervention groups (n=43 vs. 51) were similar in age, sex, and disease severity, though the Charlson comorbidity index was significantly higher in the intervention group (p=0.007), indicating a slight imbalance that could bias mortality results	☐	☐	☐	☒

		conservatively against the intervention. (mostly yes from this but not mentioned in the study)				

Bias related to administration of intervention/exposure

4	**Were the participants included in any comparisons receiving similar treatment/care, other than the exposure or intervention of interest?**	Both groups were treated in the same hospital with similar anti-MRSA agents (vancomycin, teicoplanin, daptomycin, etc.), indicating comparable treatment conditions. The main difference was the addition of pharmacist-led interventions and bundle monitoring.	☒	☐	☐	☐

Bias related to assessment, detection and measurement of the outcome

			Yes	No	Unclear	N/A
5	**Were there multiple measurements of the outcome, both pre and post the intervention/exposure?**					
	**Outcome 1**	30-d mortality	☒	☐	☐	☐
	**Outcome 2**	Hospital mortality	☒	☐	☐	☐
	**Outcome 3**	Length of stay, da (IQR)	☒	☐	☐	☐
	**Outcome 4**	Length of treatment, da (IQR)	☒	☐	☐	☐

			Yes	No	Unclear	N/A
	**Outcome 7**	The study assessed outcomes pre- and post-intervention but did not include multiple time points within each phase. It used two defined periods (pre-AMS: Jan 2012–Apr 2013; post-AMS: May 2013–Dec 2015)	☐	☐	☐	☐

6	**Were the outcomes of participants included in any comparisons measured in the same way?**		**Yes**	**No**	**Unclear**	**N/A**
	**Outcome 1**	All outcomes and list above	☒	☐	☐	☐
		Outcomes such as 30-day mortality, hospital mortality, and bundle adherence were consistently defined and assessed using the same criteria for both groups.				

7	**Were outcomes measured in a reliable way?**	MRSA bacteremia diagnosis was confirmed by positive blood cultures, mortality data from hospital records, and bundle components (e.g., source control, follow-up cultures) from electronic medical records. Definitions were based on established guidelines (IDSA and national AMS guidelines).	**Yes**	**No**	**Unclear**	**N/A**
	**Outcomes**	All outcomes	☒	☐	☐	☐

Bias related to participant retention

			Yes	No	Unclear	N/A
8	**Was follow-up complete and if not, were differences between groups in terms of their follow-up adequately described and analyzed?**					
	**Outcome 1**		**Yes**	**No**	**Unclear**	**N/A**
	Result 1		☐	☐	☐	☒

Statistical Conclusion Validity

9	**Was appropriate statistical analysis used?**					
	**Outcomes**	Mann-Whitney U-test and Fisher's exact test were used appropriately for nonparametric and categorical data. Logistic regression was used for multivariate analysis to identify independent predictors of mortality, adjusting for potential confounders.	**Yes**	**No**	**Unclear**	**N/A**
	Results		☒	☐	☐	☐

Overall appraisal: **Include:** ☒ **Exclude:** ☐ **Seek Further Info:** ☐

Comments:

Category	Details
**Full Citation**	**Ohashi K, Matsuoka T, Shinoda Y, Fukami Y, Shindoh J, Yagi T, Yoshimura T, Sugiyama T.** ***Evaluation of treatment outcomes of patients with MRSA bacteremia following antimicrobial stewardship programs with pharmacist intervention. Int. J. Clin. Pract.*** **2018, 72, e13065.**

**DOI**	https://doi.org/10.1111/ijcp.13065
**Study Design**	**Quasi-experimental, pre–post (before-and-after) historical control study without randomization**
**Setting**	**Ogaki Municipal Hospital, a tertiary care hospital in Gifu, Japan**
**Study Period**	**Pre-intervention: January 2012 – April 2013; Post-intervention: May 2013 – December 2015**
**Population**	**Adult patients (≥18 years) diagnosed with *methicillin-resistant Staphylococcus aureus* bacteremia (MRSA-B)**
**Sample Size**	**Pre-intervention group: 43 patients; Intervention group: 51 patients**
**Inclusion Criteria**	**Adults with at least one positive blood culture for MRSA**
**Exclusion Criteria**	**Patients not treated with antimicrobials, hospital stay <4 days, death within 48 hours after MRSA detection, or palliative care cases**
**Intervention**	**Implementation of an antimicrobial stewardship (AMS) program with pharmacist intervention, including: – Daily pharmacist follow-up of MRSA-positive cases – Immediate pharmacist feedback to attending physicians – Use of an MRSA bacteremia treatment "bundle" – AST case conferences twice weekly with infectious disease specialists, pharmacists, nurses, and microbiologists – Pharmacist recommendations on early initiation, dose adjustment, duration, and follow-up cultures**
**Comparator**	**Historical control (pre-intervention period before AMS and pharmacist involvement)**
**AMS Intervention Components**	**– Bundle care (appropriate duration ≥14/28 days, early anti-MRSA therapy within 24 h, follow-up blood cultures, early source control, echocardiography, dose adjustment of vancomycin/teicoplanin) – Therapeutic Drug Monitoring (TDM) – Pharmacist-led audit, feedback, and education to physicians**
**Pharmacist Role**	**Pharmacists served as active members of the Antimicrobial Stewardship Team (AST), receiving real-time positive culture reports, proposing e**

Additional points for reference in appraisal

**Were confounding factors identified and accounted for?**	**Potential confounders (e.g., age, severity, comorbidities) should be discussed or adjusted for in analysis.**	**Yes. Variables such as sepsis, SOFA score, and Charlson index were considered in univariate and multivariate models. The intervention remained a significant independent factor reducing mortality (adjusted OR for 30-day mortality = 0.33; hospital mortality = 0.20).**

11. Were participants included in comparisons similar at baseline?	Baseline comparability should be assessed across groups to ensure fairness.	Largely yes, though minor imbalance in comorbidity index (higher in intervention group) was present, likely making results conservative. Other parameters (age, sex, infection site, severity) were comparable.
12. Were there any subgroups or analyses to identify differences in effect across populations?	Subgroup analysis enhances understanding of the intervention's generalizability.	Not specifically performed. The study did not stratify by infection complexity, age, or ward type, though complicated vs. uncomplicated bacteremia was described.
13. Was the study free from other sources of bias?	Biases such as historical changes, reporting bias, or selection bias should be minimized.	Partially. As a single-center, retrospective, non-randomized study, potential historical or selection bias exists. However, same setting, consistent diagnostic criteria, and objective outcomes mitigate this risk.
14. Overall appraisal: Should the study be included in a systematic review?	Determine whether methodological quality is acceptable for inclusion.	Yes. The study demonstrates clear pre/post design, transparent reporting, objective outcome measures, and appropriate statistical adjustment. Moderate risk of bias due to lack of randomization and potential time-related confounding. Suitable for inclusion in systematic review and meta-analysis.

Summary of Appraisal

- Strengths:
  - Clear causal relationship and defined intervention.
  - Well-described pharmacist-led AMS program with measurable adherence outcomes.
  - Statistically significant reduction in mortality.
  - Strong internal validity through consistent measurement and defined inclusion/exclusion criteria.
- Limitations:
  - Non-randomized design (historical control) may introduce temporal or selection bias.
  - Single-center, moderate sample size limits generalizability.
  - No long-term follow-up or cost analysis.

? Overall Risk of Bias: Low to moderate.

? Inclusion Decision: Include in systematic review and meta-analysis due to methodological rigor and direct relevance to pharmacist-led AMS in MDRO infections.

RoB Assessor: Mouda Hamdan	Date of Appraisal: 14/11/2025	Record Number: 1
Study Author: McCrink K., et al	Study Title: Impact of a real-time diagnostic and antimicrobial stewardship workflow on time to appropriate therapy for infections caused by multidrug-resistant Gram-negative organisms	Study Year: 2023

	Internal Validity	Choice - Comments/Justification	Yes	No	Unclear	N/A
**Bias related to temporal precedence**						
1	Is it clear in the study what is the "cause" and what is the "effect" (i.e. there is no confusion about which variable comes first)?		☒	☐	☐	☐
**Bias related to selection and allocation**						
2	Was there a control group?		☒	☐	☐	☐
**Bias related to confounding factors**						
3	Were participants included in any comparisons similar?		☒	☐	☐	☐
**Bias related to administration of intervention/exposure**						
4	Were the participants included in any comparisons receiving similar treatment/care, other than the exposure or intervention of interest?		☒	☐	☐	☐

Bias related to assessment, detection and measurement of the outcome

5	Were there multiple measurements of the outcome, both pre and post the intervention/exposure?		Yes	No	Unclear	N/A
	**Outcome 1**	Time to appropriate therapy (hours)	☒	☐	☐	☐
	**Outcome 2**	Time from index culture to final susceptibilities (hours)	☒	☐	☐	☐
	**Outcome 3**	30-day mortality	☒	☐	☐	☐
	**Outcome 4**	Hospital length of stay	☒	☐	☐	☐
	**Outcome 5**	AKI	☒	☐	☐	☐
	**Outcome 6**	Time to bacteremia clearence	☒	☐	☐	☐
	**Outcome 7**	Dose appropriate for renal function	☒	☐	☐	☐

6	Were the outcomes of participants included in any comparisons measured in the same way?		Yes	No	Unclear	N/A
	**Outcome 1**	Time to appropriate therapy (hours)	☒	☐	☐	☐
	**Outcome 2**	Time from index culture to final susceptibilities (hours)	☒	☐	☐	☐
	**Outcome 3**	30-day mortality	☒	☐	☐	☐

			Yes	No	Unclear	N/A
	**Outcome 4**	Hospital length of stay	☒	☐	☐	☐
	**Outcome 5**	AKI	☒	☐	☐	☐
	**Outcome 6**	Time to bacteremia clearence	☒	☐	☐	☐
	**Outcome 7**	Dose appropriate for renal function	☒	☐	☐	☐

7	**Were outcomes measured in a reliable way?**		**Yes**	**No**	**Unclear**	**N/A**
	**Outcome 1**	Time to appropriate therapy (hours)	☒	☐	☐	☐
	**Outcome 2**	Time from index culture to final susceptibilities (hours)	☒	☐	☐	☐
	**Outcome 3**	30-day mortality	☒	☐	☐	☐
	**Outcome 4**	Hospital length of stay	☒	☐	☐	☐
	**Outcome 5**	AKI	☒	☐	☐	☐
	**Outcome 6**	Time to bacteremia clearence	☒	☐	☐	☐
	**Outcome 7**	Dose appropriate for renal function	☒	☐	☐	☐

Bias related to participant retention

8	**Was follow-up complete and if not, were differences between groups in terms of their follow-up adequately described and analyzed?**					
	**Outcome 1**	Time to appropriate therapy (hours)	**Yes**	**No**	**Unclear**	**N/A**
	Result 1	Pre-AMS 103 (76–156) vs post-AMS 75 (56–100)	☒	☐	☐	☐
	Result 2		☐	☐	☐	☐
	Result 3		☐	☐	☐	☐
	**Outcome 2**	Time from index culture to final susceptibilities (hours)	**Yes**	**No**	**Unclear**	**N/A**
	Result 1	Pre-AMS 123 (95–139) vs post-AMS 93 (74–113)	☒	☐	☐	☐
	Result 2		☐	☐	☐	☐
	Result 3		☐	☐	☐	☐
	**Outcome 3**	30-day mortality	**Yes**	**No**	**Unclear**	**N/A**
	Result 1	Pre-AMS 11 (23.9) vs post-AMS 12 (22.6)	☒	☐	☐	☐
	Result 2		☐	☐	☐	☐
	Result 3		☐	☐	☐	☐
	**Outcome 4**	Hospital length of stay	**Yes**	**No**	**Unclear**	**N/A**
	Result 1	Pre-AMS 64 (40–125) vs post-AMS 58 (25–104)	☒	☐	☐	☐
	Result 2		☐	☐	☐	☐

		Yes	No	Unclear	N/A
Result 3		☐	☐	☐	☐
**Outcome 5**	AKI	**Yes**	**No**	**Unclear**	**N/A**
Result 1	Pre-AMS 13 (28.3) vs post-AMS 15 (28.3)	☒	☐	☐	☐
Result 2		☐	☐	☐	☐
Result 3		☐	☐	☐	☐
**Outcome 6**	Time to bacteremia clearance	**Yes**	**No**	**Unclear**	**N/A**
Result 1	Pre-AMS 67 (44–114) vs post-AMS 74 (59–98)	☒	☐	☐	☐
Result 2		☐	☐	☐	☐
Result 3		☐	☐	☐	☐
**Outcome 7**	Dose appropriate for renal function	**Yes**	**No**	**Unclear**	**N/A**
Result 1	Pre-AMS 33 (71.7) vs post-AMS 48 (90.6)	☒	☐	☐	☐
Result 2		☐	☐	☐	☐
Result 3		☐	☐	☐	☐

Statistical Conclusion Validity

9	**Was appropriate statistical analysis used?**					
	**Outcome 1**	Time to appropriate therapy (hours)	**Yes**	**No**	**Unclear**	**N/A**

Result 1	Pre-AMS 103 (76–156) vs post-AMS 75 (56–100)	☒	☐	☐	☐
Result 2		☐	☐	☐	☐
Result 3		☐	☐	☐	☐
**Outcome 2**	Time from index culture to final susceptibilities (hours)	**Yes**	**No**	**Unclear**	**N/A**
Result 1	Pre-AMS 123 (95–139) vs post-AMS 93 (74–113)	☒	☐	☐	☐
Result 2		☐	☐	☐	☐
Result 3		☐	☐	☐	☐
**Outcome 3**	30-day mortality	**Yes**	**No**	**Unclear**	**N/A**
Result 1	Pre-AMS 11 (23.9) vs post-AMS 12 (22.6)	☒	☐	☐	☐
Result 2		☐	☐	☐	☐
Result 3		☐	☐	☐	☐
**Outcome 4**	**Hospital length of stay**	**Yes**	**No**	**Unclear**	**N/A**
Result 1	Pre-AMS 64 (40–125) vs post-AMS 58 (25–104)	☒	☐	☐	☐
Result 2		☐	☐	☐	☐
Result 3		☐	☐	☐	☐
**Outcome 5**	AKI	**Yes**	**No**	**Unclear**	**N/A**
Result 1	Pre-AMS 13 (28.3) vs post-AMS 15 (28.3)	☒	☐	☐	☐

		Yes	No	Unclear	N/A
Result 2		☐	☐	☐	☐
Result 3		☐	☐	☐	☐
**Outcome 6**	Time to bacteremia clearance	**Yes**	**No**	**Unclear**	**N/A**
Result 1	Pre-AMS 67 (44–114) vs post-AMS 74 (59–98)	☒	☐	☐	☐
Result 2		☐	☐	☐	☐
Result 3		☐	☐	☐	☐
**Outcome 7**	Dose appropriate for renal function	**Yes**	**No**	**Unclear**	**N/A**
Result 1	Pre-AMS 33 (71.7) vs post-AMS 48 (90.6)	☒	☐	☐	☐
Result 2		☐	☐	☐	☐
Result 3		☐	☐	☐	☐

Overall appraisal: **Include:** ☒ **Exclude:** ☐ **Seek Further Info:** ☐

Comments:

RoB Assessor: Dima Baker	Date of Appraisal: 17/Nov/2025	Record Number:
Study Author: Swingler et al.	Study Title: Effect of a pharmacy-driven rapid bacteremia response program on outcomes in adu patients with extended-spectrum beta-lactamase bacteremia: A retrospective, quasi-experimental study	Study Year: 2024

Internal Validity		Choice - Comments/Justification	Yes	No	Unclear	N/A
**Bias related to temporal precedence**						
1	Is it clear in the study what is the "cause" and what is the "effect" (i.e. there is no confusion about which variable comes first)?		☒	☐	☐	☐
**Bias related to selection and allocation**						
2	Was there a control group?		☒	☐	☐	☐
**Bias related to confounding factors**						
3	Were participants included in any comparisons similar?		☒	☐	☐	☐
**Bias related to administration of intervention/exposure**						
4	Were the participants included in any comparisons receiving similar treatment/care, other than the exposure or intervention of interest?		☐	☐	☒	☐

Bias related to assessment, detection and measurement of the outcome

5	Were there multiple measurements of the outcome, both pre and post the intervention/exposure?		Yes	No	Unclear	N/A
	Outcome 1	In-hospital mortality	☒	☐	☐	☐
	Outcome 2	30-day mortality	☒	☐	☐	☐
	Outcome 3	Length of hospital stay	☒	☐	☐	☐
	Outcome 4	Length of ICU stay	☒	☐	☐	☐
	Outcome 5	Length of therapy	☒	☐	☐	☐
	Outcome 6	Time to active therapy	☒	☐	☐	☐
	Outcome 7		☐	☐	☐	☐

6	Were the outcomes of participants included in any comparisons measured in the same way?		Yes	No	Unclear	N/A
	Outcome 1	In-hospital mortality	☒	☐	☐	☐
	Outcome 2	30-day mortality	☒	☐	☐	☐
	Outcome 3	Length of hospital stay	☒	☐	☐	☐
	Outcome 4	Length of ICU stay	☒	☐	☐	☐
	Outcome 5	Length of therapy	☒	☐	☐	☐

			Yes	No	Unclear	N/A
	**Outcome 6**	Time to active therapy	☒	☐	☐	☐
	**Outcome 7**		☐	☐	☐	☐

7	**Were outcomes measured in a reliable way?**		**Yes**	**No**	**Unclear**	**N/A**
	**Outcome 1**	In-hospital mortality	☒	☐	☐	☐
	**Outcome 2**	30-day mortality	☒	☐	☐	☐
	**Outcome 3**	Length of hospital stay	☒	☐	☐	☐
	**Outcome 4**	Length of ICU stay	☒	☐	☐	☐
	**Outcome 5**	Length of therapy	☒	☐	☐	☐
	**Outcome 6**	Time to active therapy	☒	☐	☐	☐
	**Outcome 7**		☐	☐	☐	☐

Bias related to participant retention

8	**Was follow-up complete and if not, were differences between groups in terms of their follow-up adequately described and analyzed?**					
	**Outcome 1**	In-hospital mortality	**Yes**	**No**	**Unclear**	**N/A**
	Result 1	Pre = 19%, post = 17%, p-value = 0.854.	☒	☐	☐	☐

		Yes	No	Unclear	N/A
Result 2		☐	☐	☐	☐
Result 3		☐	☐	☐	☐
**Outcome 2**	30-day mortality	**Yes**	**No**	**Unclear**	**N/A**
Result 1	pre = 20%, post = 21%, p-value = >0.999	☒	☐	☐	☐
Result 2		☐	☐	☐	☐
Result 3		☐	☐	☐	☐
**Outcome 3**	Length of hospital stay, days, median [IQR]	**Yes**	**No**	**Unclear**	**N/A**
Result 1	pre = 6.4 [4.9, 10.1], post = 6.8 [4.4, 12.8], p-value = 0.757	☒	☐	☐	☐
Result 2		☐	☐	☐	☐
Result 3		☐	☐	☐	☐
**Outcome 4**	Length of ICU stay, days, median [IQR]	**Yes**	**No**	**Unclear**	**N/A**
Result 1	pre = 2.5 [1.7, 7.1], post = 3.9 [1.8, 6.6], p-value = 0.577	☒	☐	☐	☐
Result 2		☐	☐	☐	☐
Result 3		☐	☐	☐	☐
**Outcome 5**	Length of therapy, days, median [IQR]	**Yes**	**No**	**Unclear**	**N/A**

		Yes	No	Unclear	N/A
Result 1	pre =13.2 [7.8, 17.6], post = 14.8 [10.1, 17.0], p-value = 0.559	☒	☐	☐	☐
Result 2		☐	☐	☐	☐
Result 3		☐	☐	☐	☐
**Outcome 6**	Time to active therapy, hours, median [IQR]	**Yes**	**No**	**Unclear**	**N/A**
Result 1	pre = 22.8 [3.7, 63.8], post = 16.4 [2.9, 19.3], p-value = 0.001	☒	☐	☐	☐
Result 2		☐	☐	☐	☐
Result 3		☐	☐	☐	☐
**Outcome 7**		**Yes**	**No**	**Unclear**	**N/A**
Result 1		☐	☐	☐	☐
Result 2		☐	☐	☐	☐
Result 3		☐	☐	☐	☐

Statistical Conclusion Validity

9	**Was appropriate statistical analysis used?**					
	**Outcome 1**	In-hospital mortality	**Yes**	**No**	**Unclear**	**N/A**
	Result 1	Pre = 19%, post = 17%, p-value = 0.854	☒	☐	☐	☐

Result 2		☐	☐	☐	☐
Result 3		☐	☐	☐	☐
**Outcome 2**	30-day mortality	**Yes**	**No**	**Unclear**	**N/A**
Result 1	pre = 20%, post = 21%, p-value = >0.999	☒	☐	☐	☐
Result 2		☐	☐	☐	☐
Result 3		☐	☐	☐	☐
**Outcome 3**	Length of hospital stay	**Yes**	**No**	**Unclear**	**N/A**
Result 1	pre = 6.4 [4.9, 10.1], post = 6.8 [4.4, 12.8], p-value = 0.757	☒	☐	☐	☐
Result 2		☐	☐	☐	☐
Result 3		☐	☐	☐	☐
**Outcome 4**	Length of ICU stay, days, median [IQR]	**Yes**	**No**	**Unclear**	**N/A**
Result 1	pre = 2.5 [1.7, 7.1], post = 3.9 [1.8, 6.6], p-value = 0.577	☒	☐	☐	☐
Result 2		☐	☐	☐	☐
Result 3		☐	☐	☐	☐
**Outcome 5**	Length of therapy, days, median [IQR]	**Yes**	**No**	**Unclear**	**N/A**
Result 1	pre =13.2 [7.8, 17.6], post = 14.8 [10.1, 17.0], p-value = 0.559	☒	☐	☐	☐

		Yes	No	Unclear	N/A
Result 2		☐	☐	☐	☐
Result 3		☐	☐	☐	☐
**Outcome 6**	Time to active therapy, hours, median [IQR]	**Yes**	**No**	**Unclear**	**N/A**
Result 1	pre = 22.8 [3.7, 63.8], post = 16.4 [2.9, 19.3], p-value = 0.001	☒	☐	☐	☐
Result 2		☐	☐	☐	☐
Result 3		☐	☐	☐	☐
**Outcome 7**		**Yes**	**No**	**Unclear**	**N/A**
Result 1		☐	☐	☐	☐
Result 2		☐	☐	☐	☐
Result 3		☐	☐	☐	☐

Overall appraisal: **Include:** ☒ **Exclude:** ☐ **Seek Further Info:** ☐

Comments:

RoB Assessor: Zainab AlMuhsin	Date of Appraisal: 09/11/2025	Record Number: 10.1186/s12879-021-06458-0
**Study Author: Wong et al.**	**Study Title:** Retrospective assessment of antimicrobial stewardship initiative in outpatient use of ertapenem for uncomplicated ESBL-EB UTI.	**Study Year: 2021**

	Internal Validity	Choice - Comments/Justification	Yes	No	Unclear	N/A
**Bias related to temporal precedence**						
1	Is it clear in the study what is the "cause" and what is the "effect" (i.e. there is no confusion about which variable comes first)?	The cause is the antimicrobial stewardship initiative (ASI); the effect is the change in ertapenem use and clinical outcomes.	☒	☐	☐	☐
**Bias related to selection and allocation**						
2	Was there a control group?	Pre-intervention group (before ASI) served as the control.	☒	☐	☐	☐
**Bias related to confounding factors**						
3	Were participants included in any comparisons similar?	Baseline demographics (age, CrCl, organism distribution) were comparable between pre and post groups (Table 2, $p > 0.05$).	☒	☐	☐	☐
**Bias related to administration of intervention/exposure**						

			Yes	No	Unclear	N/A
4	**Were the participants included in any comparisons receiving similar treatment/care, other than the exposure or intervention of interest?**	All were outpatients treated for ESBL-EB UTI; only the stewardship intervention differed.	☒	☐	☐	☐

Bias related to assessment, detection and measurement of the outcome

5	**Were there multiple measurements of the outcome, both pre and post the intervention/exposure?**		**Yes**	**No**	**Unclear**	**N/A**
	**Outcome 1:** Ertapenem utilization	Measured in both pre- and post-ASI periods (ertapenem use reduction assessed)	☒	☐	☐	☐
	**Outcome 2:** Clinical resolution	Evaluated pre- and post-ASI to confirm no loss of cure with reduced ertapenem use	☒	☐	☐	☐
	**Outcome 3:** Recurrence/relapse rate	Assessed for both periods; no relapses reported in either.	☒	☐	☐	☐
	**Outcome 4:** Adverse drug reactions	Monitored for both groups; none reported.	☒	☐	☐	☐
	**Outcome 5:** Antimicrobial cost	Compared before and after ASI (ertapenem cost decreased by $3,329).	☒	☐	☐	☐
	**Outcome 6**		☐	☐	☐	☐
	**Outcome 7**		☐	☐	☐	☐

6	**Were the outcomes of participants included in any comparisons measured in the same way?**		**Yes**	**No**	**Unclear**	**N/A**

			Yes	No	Unclear	N/A
	**Outcome 1:** Ertapenem utilization	Measured the same way pre- and post-ASI using prescription data from the same pharmacy database.	☒	☐	☐	☐
	**Outcome 2:** Clinical resolution	Assessed through chart review and symptom documentation using identical criteria in both periods	☒	☐	☐	☐
	**Outcome 3:** Recurrence/relapse rate	Defined by return of symptoms or positive culture within the same timeframe for both groups	☒	☐	☐	☐
	**Outcome 4:** Adverse drug reactions	Documented via consistent EMR review for both groups.	☒	☐	☐	☐
	**Outcome 5:** Antimicrobial cost	Calculated the same way using institutional pharmacy cost records pre- and post-ASI.	☒	☐	☐	☐
	**Outcome 6**		☐	☐	☐	☐
	**Outcome 7**		☐	☐	☐	☐

7	**Were outcomes measured in a reliable way?**		**Yes**	**No**	**Unclear**	**N/A**
	**Outcome 1:** Ertapenem utilization	Derived from objective pharmacy dispensing data within the KPSC system, ensuring reliability.	☒	☐	☐	☐

			Yes	No	Unclear	N/A
	**Outcome 2:** Clinical resolution	Verified by physician and pharmacist chart review using standardized documentation in the EMR.	☒	☐	☐	☐
	**Outcome 3:** Recurrence/relapse rate	Based on follow-up culture results and consistent clinical documentation across both periods.	☒	☐	☐	☐
	**Outcome 4:** Adverse drug reactions	Extracted from EMR and pharmacy safety notes, ensuring consistent data capture.	☒	☐	☐	☐
	**Outcome 5:** Antimicrobial cost	Sourced from the same financial records of the pharmacy department for both phases.	☒	☐	☐	☐
	**Outcome 6**		☐	☐	☐	☐
	**Outcome 7**		☐	☐	☐	☐

Bias related to participant retention

8	**Was follow-up complete and if not, were differences between groups in terms of their follow-up adequately described and analyzed?**					
	**Outcome 1:** Ertapenem utilization		**Yes**	**No**	**Unclear**	**N/A**
	Result 1: Pre-ASI	All outpatient ertapenem prescriptions during the pre-intervention period were included; no missing data.	☒	☐	☐	☐

		Yes	No	Unclear	N/A
Result 2: Post-ASI	Complete follow-up for all outpatient cases; full prescription data captured from the same pharmacy database.	☒	☐	☐	☐
Result 3		☐	☐	☐	☐
**Outcome 2:** Clinical resolution		**Yes**	**No**	**Unclear**	**N/A**
Result 1: Pre-ASI	All patients had documented symptom improvement or resolution; no missing follow-up data.	☒	☐	☐	☐
Result 2: Post-ASI	All patients achieved clinical cure; outcomes verified via EMR review.	☒	☐	☐	☐
Result 3		☐	☐	☐	☐
**Outcome 3:** Recurrence/relapse rate		**Yes**	**No**	**Unclear**	**N/A**
Result 1: Pre-ASI	No recurrence or relapse reported among pre-intervention patients.	☒	☐	☐	☐
Result 2: Post-ASI	No recurrence or relapse reported; all remained infection-free	☒	☐	☐	☐
Result 3		☐	☐	☐	☐
**Outcome 4:** Adverse drug reactions		**Yes**	**No**	**Unclear**	**N/A**
Result 1: Pre-ASI	No adverse reactions reported with ertapenem therapy.	☒	☐	☐	☐

		Yes	No	Unclear	N/A
Result 2: Post-ASI	No adverse events observed with aminoglycoside or ertapenem use.	☒	☐	☐	☐
Result 3		☐	☐	☐	☐
**Outcome 5:** Antimicrobial cost		**Yes**	**No**	**Unclear**	**N/A**
Result 1: Pre-ASI	Baseline cost of outpatient ertapenem therapy recorded from pharmacy database.	☒	☐	☐	☐
Result 2: Post-ASI	Total ertapenem cost decreased by $3,329 post-ASI.	☒	☐	☐	☐
Result 3		☐	☐	☐	☐
**Outcome 6**		**Yes**	**No**	**Unclear**	**N/A**
Result 1		☐	☐	☐	☐
Result 2		☐	☐	☐	☐
Result 3		☐	☐	☐	☐
**Outcome 7**		**Yes**	**No**	**Unclear**	**N/A**
Result 1		☐	☐	☐	☐
Result 2		☐	☐	☐	☐
Result 3		☐	☐	☐	☐

Statistical Conclusion Validity

9	Was appropriate statistical analysis used?					
	**Outcome 1:** Ertapenem utilization		**Yes**	**No**	**Unclear**	**N/A**
	Result 1: Pre-ASI	Baseline utilization measured and compared statistically.	☒	☐	☐	☐
	Result 2: Post-ASI	19% reduction analyzed with appropriate test ($p < 0.05$).	☒	☐	☐	☐
	Result 3		☐	☐	☐	☐
	**Outcome 2:** Clinical resolution		**Yes**	**No**	**Unclear**	**N/A**
	Result 1: Pre-ASI	Descriptive comparison of cure rates; all patients improved.	☒	☐	☐	☐
	Result 2: Post-ASI	All achieved resolution; analysis appropriate for categorical outcome.	☒	☐	☐	☐
	Result 3		☐	☐	☐	☐
	**Outcome 3:** Recurrence/relapse rate		**Yes**	**No**	**Unclear**	**N/A**
	Result 1: Pre-ASI	No recurrences; descriptive reporting appropriate.	☒	☐	☐	☐
	Result 2: Post-ASI	No recurrences; statistical comparison not required.	☒	☐	☐	☐
	Result 3		☐	☐	☐	☐

Outcome 4: Adverse drug reactions		Yes	No	Unclear	N/A
Result 1: Pre-ASI	No ADRs; descriptive analysis sufficient.	☒	☐	☐	☐
Result 2: Post-ASI	None reported; analysis method appropriate.	☒	☐	☐	☐
Result 3		☐	☐	☐	☐
**Outcome 5:** Antimicrobial cost		**Yes**	**No**	**Unclear**	**N/A**
Result 1: Pre-ASI	Baseline cost data collected.	☒	☐	☐	☐
Result 2: Post-ASI	Cost reduction ($3,329) compared descriptively between periods.	☒	☐	☐	☐
Result 3		☐	☐	☐	☐
**Outcome 6**		**Yes**	**No**	**Unclear**	**N/A**
Result 1		☐	☐	☐	☐
Result 2		☐	☐	☐	☐
Result 3		☐	☐	☐	☐
**Outcome 7**		**Yes**	**No**	**Unclear**	**N/A**
Result 1		☐	☐	☐	☐
Result 2		☐	☐	☐	☐
Result 3		☐	☐	☐	☐

Overall appraisal: **Include:** ☒ **Exclude:** ☐ **Seek Further Info:** ☐

Comments:

This retrospective pre–post study appropriately evaluated the impact of a pharmacist-led outpatient antimicrobial stewardship initiative. All outcomes (ertapenem utilization, cure, recurrence, ADRs, and cost) were measured consistently and analyzed using suitable statistical or descriptive methods. The ASI effectively reduced ertapenem use by 19% without adverse effects or relapse, achieving both clinical and economic benefits.

Limitations: Single-center, retrospective design and small post-ASI sample may limit generalizability, but internal validity remains strong.

© JBI, 2022. All rights reserved. JBI grants use of these tools for research purposes only. All other enquiries should be sent to jbisynthesis@adelaide.edu.au

CHECKLIST FOR COHORT STUDIES

Critical Appraisal tools for use in JBI Systematic Reviews

jbi.global

CRICOS Provider Number 00123M

INTRODUCTION

JBI is an international research organisation based in the Faculty of Health and Medical Sciences at the University of Adelaide, South Australia. JBI develops and delivers unique evidence-based information, software, education and training designed to improve healthcare practice and health outcomes. With over 70 Collaborating Entities, servicing over 90 countries, JBI is a recognised global leader in evidence-based healthcare.

JBI Systematic Reviews

The core of evidence synthesis is the systematic review of literature of a particular intervention, condition or issue. The systematic review is essentially an analysis of the available literature (that is, evidence) and a judgment of the effectiveness or otherwise of a practice, involving a series of complex steps. JBI takes a particular view on what counts as evidence and the methods utilised to synthesise those different types of evidence. In line with this broader view of evidence, JBI has developed theories, methodologies and rigorous processes for the critical appraisal and synthesis of these diverse forms of evidence in order to aid in clinical decision-making in healthcare. There now exists JBI guidance for conducting reviews of effectiveness research, qualitative research, prevalence/incidence, etiology/risk, economic evaluations, text/opinion, diagnostic test accuracy, mixed-methods, umbrella reviews and scoping reviews. Further information regarding JBI systematic reviews can be found in the JBI Evidence Synthesis Manual.

JBI Critical Appraisal Tools

All systematic reviews incorporate a process of critique or appraisal of the research evidence. The purpose of this appraisal is to assess the methodological quality of a study and to determine the extent to which a study has addressed the possibility of bias in its design, conduct and analysis. All papers selected for inclusion in the systematic review (that is – those that meet the inclusion criteria described in the protocol) need to be subjected to rigorous appraisal by two critical appraisers. The results of this appraisal can then be used to inform synthesis and interpretation of the results of the study. JBI Critical appraisal tools have been developed by the JBI and collaborators and approved by the JBI Scientific Committee following extensive peer review. Although designed for use in systematic reviews, JBI critical appraisal tools can also be used when creating Critically Appraised Topics (CAT), in journal clubs and as an educational tool.

© JBI, 2020. All rights reserved. JBI grants use of these tools for research purposes only. All other enquiries should be sent to jbisynthesis@adelaide.edu.au.

JBI CRITICAL APPRAISAL CHECKLIST FOR COHORT STUDIES

Yamada, K.; Imoto, W.; Yamairi, K.; Shibata, W.; Namikawa, H.; Yoshii, N.; Fujimoto, H.; Nakaie, K.; Okada, Y.; Fujita, A.; Kawaguchi, H.; Shinoda, Y.; Nakamura, Y.; Kaneko, Y.; Yoshida, H.; Kakeya, H. The Intervention by an Antimicrobial Stewardship Team Can Improve Clinical and Microbiological Outcomes of Resistant Gram-Negative Bacteria. *Journal of Infection and Chemotherapy* **2019**, *25* (12), 1001–1006. https://doi.org/10.1016/j.jiac.2019.05.026.

	Yes	No	Unclear	Not applicable
1. Were the two groups similar and recruited from the same population?	√	☐	☐	☐
2. Were the exposures measured similarly to assign people to both exposed and unexposed groups?	√	☐	☐	☐
3. Was the exposure measured in a valid and reliable way?	√	☐	☐	☐
4. Were confounding factors identified?	☐	☐	√	☐
5. Were strategies to deal with confounding factors stated?	☐	☐	√	☐
6. Were the groups/participants free of the outcome at the start of the study (or at the moment of exposure)?	√	☐	☐	☐
7. Were the outcomes measured in a valid and reliable way?	√	☐	☐	☐
8. Was the follow up time reported and sufficient to be long enough for outcomes to occur?	√	☐	☐	☐
9. Was follow up complete, and if not, were the reasons to loss to follow up described and explored?	√	☐	☐	☐
10. Were strategies to address incomplete follow up utilized?	√	☐	☐	☐
11. Was appropriate statistical analysis used?	√	☐	☐	☐

Overall appraisal: Include √ Exclude ☐ Seek further info ☐

Comments (Including reason for exclusion):

© JBI, 2020. All rights reserved. JBI grants use of these tools for research purposes only. All other enquiries should be sent to jbisynthesis@adelaide.edu.au.

JBI Item	Yes/No/Unclear	Justification
1. Cause & effect clearly stated	Yes	Intervention (ASP) occurred at a defined time (2014) and compared to pre-period
2. Similar participants in both periods	No	Age and departments differed between groups
3. Intervention independent of other changes	Unclear	Stewardship knowledge over time may change practice
4. Outcome measured similarly before/after	Yes	Same microbiology systems, same mortality definitions
5. Follow-up complete / same length	Yes	Full hospital data from both periods available
6. Outcomes measured reliably	Yes	Mortality, DOT, susceptibility all objective measurements
7. Appropriate statistical analysis	Yes	Chi-square, t-test, significance testing used
8. Confounding factors identified	No	Severity of illness not adjusted
9. Confounding factors managed	No	No regression or adjustment performed
**Overall appraisal**	Moderate	Large sample & strong outcomes but confounding not controlled

© JBI, 2020. All rights reserved. JBI grants use of these tools for research purposes only. All other enquiries should be sent to jbisynthesis@adelaide.edu.au.

EXPLANATION OF COHORT STUDIES CRITICAL APPRAISAL

How to Cite: *Moola S, Munn Z, Tufanaru C, Aromataris E, Sears K, Sfetcu R, Currie M, Qureshi R, Mattis P, Lisy K, Mu P-F. Chapter 7: Systematic reviews of etiology and risk . In: Aromataris E, Munn Z (Editors). JBI Manual for Evidence Synthesis.* JBI, 2020. Available from https://synthesismanual.jbi.global

Cohort Studies Critical Appraisal Tool

Answers: Yes, No, Unclear or Not/Applicable

1. Were the two groups similar and recruited from the same population?

Check the paper carefully for descriptions of participants to determine if patients within and across groups have similar characteristics in relation to exposure (e.g. risk factor under investigation). The two groups selected for comparison should be as similar as possible in all characteristics except for their exposure status, relevant to the study in question. The authors should provide clear inclusion and exclusion criteria that they developed prior to recruitment of the study participants.

2. Were the exposures measured similarly to assign people to both exposed and unexposed groups?

A high quality study at the level of cohort design should mention or describe how the exposures were measured. The exposure measures should be clearly defined and described in detail. This will enable reviewers to assess whether or not the participants received the exposure of interest.

3. Was the exposure measured in a valid and reliable way?

The study should clearly describe the method of measurement of exposure. Assessing validity requires that a 'gold standard' is available to which the measure can be compared. The validity of exposure measurement usually relates to whether a current measure is appropriate or whether a measure of past exposure is needed.

Reliability refers to the processes included in an epidemiological study to check repeatability of measurements of the exposures. These usually include intra-observer reliability and inter-observer reliability.

4. Were confounding factors identified?

Confounding has occurred where the estimated intervention exposure effect is biased by the presence of some difference between the comparison groups (apart from the exposure investigated/of interest). Typical confounders include baseline characteristics, prognostic factors, or concomitant exposures (e.g. smoking). A confounder is a difference between the comparison groups and it influences the direction of the study results. A high quality study at the level of cohort design will identify the potential confounders and measure them (where possible). This is difficult for studies where behavioral, attitudinal or lifestyle factors may impact on the results.

© JBI, 2020. All rights reserved. JBI grants use of these tools for research purposes only. All other enquiries should be sent to jbisynthesis@adelaide.edu.au.

5. **Were strategies to deal with confounding factors stated?**

Strategies to deal with effects of confounding factors may be dealt within the study design or in data analysis. By matching or stratifying sampling of participants, effects of confounding factors can be adjusted for. When dealing with adjustment in data analysis, assess the statistics used in the study. Most will be some form of multivariate regression analysis to account for the confounding factors measured. Look out for a description of statistical methods as regression methods such as logistic regression are usually employed to deal with confounding factors/variables of interest.

6. **Were the groups/participants free of the outcome at the start of the study (or at the moment of exposure)?**

The participants should be free of the outcomes of interest at the start of the study. Refer to the 'methods' section in the paper for this information, which is usually found in descriptions of participant/sample recruitment, definitions of variables, and/or inclusion/exclusion criteria.

7. **Were the outcomes measured in a valid and reliable way?**

Read the methods section of the paper. If for e.g. lung cancer is assessed based on existing definitions or diagnostic criteria, then the answer to this question is likely to be yes. If lung cancer is assessed using observer reported, or self-reported scales, the risk of over- or under-reporting is increased, and objectivity is compromised. Importantly, determine if the measurement tools used were validated instruments as this has a significant impact on outcome assessment validity.

Having established the objectivity of the outcome measurement (e.g. lung cancer) instrument, it's important to establish how the measurement was conducted. Were those involved in collecting data trained or educated in the use of the instrument/s? (e.g. radiographers). If there was more than one data collector, were they similar in terms of level of education, clinical or research experience, or level of responsibility in the piece of research being appraised?

8. **Was the follow up time reported and sufficient to be long enough for outcomes to occur?**

The appropriate length of time for follow up will vary with the nature and characteristics of the population of interest and/or the intervention, disease or exposure. To estimate an appropriate duration of follow up, read across multiple papers and take note of the range for duration of follow up. The opinions of experts in clinical practice or clinical research may also assist in determining an appropriate duration of follow up. For example, a longer timeframe may be needed to examine the association between occupational exposure to asbestos and the risk of lung cancer. It is important, particularly in cohort studies that follow up is long enough to enable the outcomes. However, it should be remembered that the research question and outcomes being examined would probably dictate the follow up time.

© JBI, 2020. All rights reserved. JBI grants use of these tools for research purposes only. All other enquiries should be sent to jbisynthesis@adelaide.edu.au.

9. Was follow up complete, and if not, were the reasons to loss to follow up described and explored?

It is important in a cohort study that a greater percentage of people are followed up. As a general guideline, at least 80% of patients should be followed up. Generally a dropout rate of 5% or less is considered insignificant. A rate of 20% or greater is considered to significantly impact on the validity of the study. However, in observational studies conducted over a lengthy period of time a higher dropout rate is to be expected. A decision on whether to include or exclude a study because of a high dropout rate is a matter of judgement based on the reasons why people dropped out, and whether dropout rates were comparable in the exposed and unexposed groups.

Reporting of efforts to follow up participants that dropped out may be regarded as an indicator of a well conducted study. Look for clear and justifiable description of why people were left out, excluded, dropped out etc. If there is no clear description or a statement in this regards, this will be a 'No'.

10. Were strategies to address incomplete follow up utilized?

Some people may withdraw due to change in employment or some may die; however, it is important that their outcomes are assessed. Selection bias may occur as a result of incomplete follow up. Therefore, participants with unequal follow up periods must be taken into account in the analysis, which should be adjusted to allow for differences in length of follow up periods. This is usually done by calculating rates which use person-years at risk, i.e. considering time in the denominator.

11. Was appropriate statistical analysis used?

As with any consideration of statistical analysis, consideration should be given to whether there was a more appropriate alternate statistical method that could have been used. The methods section of cohort studies should be detailed enough for reviewers to identify which analytical techniques were used (in particular, regression or stratification) and how specific confounders were measured.

For studies utilizing regression analysis, it is useful to identify if the study identified which variables were included and how they related to the outcome. If stratification was the analytical approach used, were the strata of analysis defined by the specified variables? Additionally, it is also important to assess the appropriateness of the analytical strategy in terms of the assumptions associated with the approach as differing methods of analysis are based on differing assumptions about the data and how it will respond.

© JBI, 2020. All rights reserved. JBI grants use of these tools for research purposes only. All other enquiries should be sent to jbisynthesis@adelaide.edu.au.

**RoB Assessor:** Zainab AlMuhsin	**Date of Appraisal:** 8/11/2025	**Record Number:** 10.1186/s40780-021-00206-x
**Study Author:** Nakamura S et al.	**Study Title:** Impact of an antimicrobial stewardship in a 126-bed community hosp with close communication between pharmacists working on post-prescription audit, ward pharmacists, and the antimicrobial stewardship team	**Study Year:** 2021

Internal Validity		Choice - Comments/Justification	Yes	No	Unclear	N/A
**Bias related to temporal precedence**						
1	**Is it clear in the study what is the "cause" and what is the "effect" (i.e. there is no confusion about which variable comes first)?**	The cause is ASP implementation; the effect is reduced antibiotic use and improved outcomes. Temporal order is clear.	☒	☐	☐	☐
**Bias related to selection and allocation**						
2	**Was there a control group?**	There was no independent control group; the study used a pre- and post-intervention comparison within the same hospital.	☐	☒	☐	☐
**Bias related to confounding factors**						
3	**Were participants included in any comparisons similar?**	Participants in both pre- and post-implementation phases were hospitalized patients receiving antimicrobial therapy at the same 126-bed hospital, with similar baseline characteristics and case mix.	☒	☐	☐	☐

Bias related to administration of intervention/exposure

			Yes	No	Unclear	N/A
4	**Were the participants included in any comparisons receiving similar treatment/care, other than the exposure or intervention of interest?**	All patients received standard hospital care and antibiotic therapy following the same institutional guidelines; only the addition of pharmacist-led ASP activities differed between groups.	☒	☐	☐	☐

Bias related to assessment, detection and measurement of the outcome

			Yes	No	Unclear	N/A
5	**Were there multiple measurements of the outcome, both pre and post the intervention/exposure?**					
	**Outcome 1:** Antimicrobial use	Antimicrobial use (DDD/1000 patient-days) was measured once per study phase (pre- and post-implementation) and compared overall, not repeatedly over time.	☐	☒	☐	☐
	**Outcome 2:** Duration of therapy	Duration of therapy was assessed once for each period (average days of antibiotic use pre vs post), without repeated measurements within the same patients.	☐	☒	☐	☐
	**Outcome 3:** Antimicrobial cost	Antimicrobial cost was calculated once per phase (pre- vs post-ASP) as total and per patient-day expenditure; no repeated cost measurements were performed.	☐	☒	☐	☐

	**Outcome 4:** Length of hospital stay (LOS)	It was analyzed once for each phase as an average per patient; it wasn’t measured repeatedly within individuals.	☐	☒	☐	☐
	**Outcome 5:** Clinical outcomes	Clinical outcomes (infection cure rate and 30-day mortality) were measured once for each study phase and compared overall between pre- and post-intervention groups.	☐	☒	☐	☐
	**Outcome 6:** Microbiological outcomes	Microbiological outcomes (resistance trends of MRSA and ESBL-producing organisms) were assessed once per phase using annual culture data; no repeated or continuous measurements were performed.	☐	☒	☐	☐
	**Outcome 7**		☐	☐	☐	☐

6	**Were the outcomes of participants included in any comparisons measured in the same way?**		**Yes**	**No**	**Unclear**	**N/A**
	**Outcome 1:** Antimicrobial use	Antimicrobial use was measured in the same standardized way for both phases, using DDD per 1000 patient-days calculated from pharmacy dispensing data following WHO ATC/DDD methodology.	☒	☐	☐	☐
	**Outcome 2:** Duration of therapy	Duration of therapy was calculated the same way in both phases, using the average number of antibiotic days per patient based on	☒	☐	☐	☐

			Yes	No	Unclear	N/A
		electronic prescribing and administration records.				
	**Outcome 3:** Antimicrobial cost	Antimicrobial cost was measured consistently in both phases using total antibiotic expenditure per 1000 patient-days obtained from the hospital pharmacy's financial records.	☒	☐	☐	☐
	**Outcome 4:** Length of hospital stay (LOS)	Length of stay was measured the same way pre and post, using the average number of hospitalization days per patient from electronic medical records.	☒	☐	☐	☐
	**Outcome 5:** Clinical outcomes	Clinical outcomes were measured identically in both phases, using infection cure rate and 30-day mortality obtained from patient records and discharge summaries.	☒	☐	☐	☐
	**Outcome 6:** Microbiological outcomes		☐	☐	☐	☐
	**Outcome 7**		☐	☐	☐	☐

7	**Were outcomes measured in a reliable way?**		**Yes**	**No**	**Unclear**	**N/A**
	**Outcome 1:** Antimicrobial use	Data collected from reliable hospital records using standardized methods (WHO DDD, clinical and lab criteria).	☒	☐	☐	☐

**Outcome 2:** Duration of therapy	Duration of therapy (DOT) was obtained from electronic prescribing data, ensuring accurate and consistent recording across both periods.	☒	☐	☐	☐
**Outcome 3:** Antimicrobial cost	Cost data were extracted from pharmacy financial records using the same calculation method in both phases, ensuring reliability.	☒	☐	☐	☐
**Outcome 4:** Length of hospital stay (LOS)	Length of stay was derived from hospital admission and discharge records, providing consistent and reliable data.	☒	☐	☐	☐
**Outcome 5:** Clinical outcomes	Cure rate and 30-day mortality were determined from patient charts using consistent clinical criteria.	☒	☐	☐	☐
**Outcome 6:** Microbiological outcomes	Resistance data were obtained from standardized lab reports using the same testing procedures in both phases.	☒	☐	☐	☐
**Outcome 7**		☐	☐	☐	☐

Bias related to participant retention

8	**Was follow-up complete and if not, were differences between groups in terms of their follow-up adequately described and analyzed?**					
	**Outcome 1:** Antimicrobial use		**Yes**	**No**	**Unclear**	**N/A**

			Yes	No	Unclear	N/A
	Result 1: Total antimicrobial use	Complete data from all inpatient prescriptions; no loss to follow-up.	☒	☐	☐	☐
	Result 2: Carbapenems	All carbapenem dispensing data captured; fully analyzed.	☒	☐	☐	☐
	Result 3: Fluoroquinolones	Usage data complete across both periods.	☒	☐	☐	☐
	Results 4: 3rd gen Cephalosporins	All prescriptions included; no missing records.	☒	☐	☐	☐
	Results 5: Antipseudomonal penicillins & other agents	All included in hospital pharmacy database; complete follow-up.	☒	☐	☐	☐
**Outcome 2:** Duration of therapy			**Yes**	**No**	**Unclear**	**N/A**
	Result 1: Total antimicrobial therapy	Complete prescribing data available for all inpatients; no missing cases.	☒	☐	☐	☐
	Result 2: Carbapenem therapy	All carbapenem prescriptions analyzed; full dataset included.	☒	☐	☐	☐
	Result 3: Fluoroquinolone therapy	Data complete from hospital records; no loss to follow-up.	☒	☐	☐	☐
**Outcome 3:** Antimicrobial cost			**Yes**	**No**	**Unclear**	**N/A**
	Result 1: Total antimicrobial cost	Complete pharmacy financial data analyzed for both periods; no missing records.	☒	☐	☐	☐

			Yes	No	Unclear	N/A
	Result 2: Carbapenem cost	All carbapenem expenses captured; data fully available.	☒	☐	☐	☐
	Result 3: Fluoroquinolone cost	Complete cost data included from accounting database.	☒	☐	☐	☐
**Outcome 4:** Length of hospital stay (LOS)			**Yes**	**No**	**Unclear**	**N/A**
	Result 1: Average hospital stay	Admission and discharge data complete for all patients; no missing values.	☒	☐	☐	☐
	Result 2: By infection type (if reported)	All cases included in analysis; follow-up complete in both phases.	☒	☐	☐	☐
	Result 3		☐	☐	☐	☐
**Outcome 5:** Clinical outcomes			**Yes**	**No**	**Unclear**	**N/A**
	Result 1: Infection cure rate	All treated patients were assessed for clinical response; no missing outcome data.	☒	☐	☐	☐
	Result 2: 30-day mortality	Mortality tracked for all included patients using hospital records; full follow-up achieved.	☒	☐	☐	☐
	Result 3		☐	☐	☐	☐
**Outcome 6:** Microbiological outcomes			**Yes**	**No**	**Unclear**	**N/A**
	Result 1: MRSA resistance rate	All MRSA isolates analyzed; complete lab data available.	☒	☐	☐	☐
	Result 2: ESBL-producing organism rate	All ESBL isolates included in resistance trend analysis; no missing results.	☒	☐	☐	☐

			Yes	No	Unclear	N/A
	Result 3		☐	☐	☐	☐
**Outcome 7**			**Yes**	**No**	**Unclear**	**N/A**
	Result 1		☐	☐	☐	☐
	Result 2		☐	☐	☐	☐
	Result 3		☐	☐	☐	☐

Statistical Conclusion Validity

9	**Was appropriate statistical analysis used?**						
	**Outcome 1:** Antimicrobial use			**Yes**	**No**	**Unclear**	**N/A**
		Result 1: Total antimicrobial use	Compared pre vs post using appropriate statistical test (significant decrease, $p < 0.05$)	☒	☐	☐	☐
		Result 2: Carbapenems	Statistical comparison showed significant reduction ($p < 0.01$)	☒	☐	☐	☐
		Result 3: Fluoroquinolones	Appropriate comparative test applied ($p < 0.05$).	☒	☐	☐	☐
		Results 4: 3rd gen Cephalosporins	Analysis confirmed significant decrease ($p < 0.05$).	☒	☐	☐	☐
		Results 5: Antipseudomonal penicillins & other agents	Compared descriptively; appropriate method for nonsignificant change.	☒	☐	☐	☐

**Outcome 2:** Duration of therapy			Yes	No	Unclear	N/A
	Result 1: Total antimicrobial therapy	Mean comparison test (e.g., t-test); significant difference ($p = 0.004$).	☒	☐	☐	☐
	Result 2: Carbapenem therapy	Statistical test confirmed reduction ($p < 0.001$).	☒	☐	☐	☐
	Result 3: Fluoroquinolone therapy	Proper comparative test used ($p = 0.011$).	☒	☐	☐	☐
**Outcome 3:** Antimicrobial cost			**Yes**	**No**	**Unclear**	**N/A**
	Result 1: Total antimicrobial cost	Compared using suitable parametric test; significant reduction ($p < 0.05$).	☒	☐	☐	☐
	Result 2: Carbapenem cost	Appropriate continuous-variable test; significant decrease.	☒	☐	☐	☐
	Result 3: Fluoroquinolone cost	Statistical analysis appropriate; costs significantly lower post-ASP.	☒	☐	☐	☐
**Outcome 4:** Length of hospital stay (LOS)			**Yes**	**No**	**Unclear**	**N/A**
	Result 1: Average hospital stays	Compared with appropriate test (no significant difference reported).	☒	☐	☐	☐
	Result 2: By infection type (if reported)	Statistical comparison applied; no loss of data.	☒	☐	☐	☐
	Result 3		☐	☐	☐	☐

**Outcome 5:** Clinical outcomes			Yes	No	Unclear	N/A
	Result 1: Infection cure rate	Compared using Fisher's exact or χ^2; no significant change.	☒	☐	☐	☐
	Result 2: 30-day mortality	Appropriate categorical test applied; non-significant difference.	☒	☐	☐	☐
	Result 3		☐	☐	☐	☐
**Outcome 6:** Microbiological outcomes			**Yes**	**No**	**Unclear**	**N/A**
	Result 1: MRSA resistance rate	Statistical comparison (χ^2 or Fisher's exact) showed no significant increase.	☒	☐	☐	☐
	Result 2: ESBL-producing organisms	Appropriate test used; resistance rates stable post-intervention.	☒	☐	☐	☐
	Result 3		☐	☐	☐	☐
**Outcome 7**			**Yes**	**No**	**Unclear**	**N/A**
	Result 1		☐	☐	☐	☐
	Result 2		☐	☐	☐	☐
	Result 3		☐	☐	☐	☐

Overall appraisal: **Include:** ☒ **Exclude:** ☐ **Seek Further Info:** ☐

Comments:

The study meets nearly all validity criteria for a quasi-experimental design. The intervention and outcomes were clearly defined, participants were comparable, outcomes were measured consistently and reliably, and follow-up was complete. Appropriate statistical tests were applied across all analyses, supporting the robustness of the findings.

Minor limitations include the lack of a concurrent control group and potential influence of unmeasured confounders, but these are expected in a pre–post design. Overall, internal validity is strong, and the results are reliable for inclusion in evidence synthesis.

© JBI, 2022. All rights reserved. JBI grants use of these tools for research purposes only. All other enquiries should be sent to jbisynthesis@adelaide.edu.au

Internal Validity		Choice - Comments/Justification	Yes	No	Unclear	N/A
**Bias related to temporal precedence**						
1	**Is it clear in the study what is the "cause" and what is the "effect" (i.e. there is no confusion about which variable comes first)?**	Intervention clearly defined (real-time EMP notification + CDTM workflow); primary outcome defined (30-day ED revisit for treatment failure).	☒	☐	☐	☐
**Bias related to selection and allocation**						
2	**Was there a control group?**	Pre-implementation period served as historical comparator (no concurrent randomized control).	☒	☐	☐	☐
**Bias related to confounding factors**						
3	**Were participants included in any comparisons similar?**	characteristics largely similar, but female sex differed (pre 65% vs post 42.9%, p=0.010) and VRE counts differed; authors report groups comparable except noted differences.	☐	☐	☐	☒
**Bias related to administration of intervention/exposure**						
4	**Were the participants included in any comparisons receiving similar treatment/care, other than the exposure or intervention of interest?**	Both groups were treated in the same hospital with similar anti-MRSA agents (vancomycin, teicoplanin, daptomycin, etc.), indicating comparable treatment conditions. The main difference was the addition of pharmacist-led interventions and bundle monitoring.	☒	☐	☐	☐

Bias related to assessment, detection and measurement of the outcome

			Yes	No	Unclear	N/A
5	**Were there multiple measurements of the outcome, both pre and post the intervention/exposure?**					
	**Outcome 1**	30-day ED revisit	☒	☐	☐	☐
	**Outcome 2**	time-to-review medians reported	☒	☐	☐	☐
	**Outcome 7**	Outcomes measured across the pre and post 15-month periods; main outcome is 30-day revisit counts; time-to-review medians reported.	☐	☐	☐	☐

			Yes	No	Unclear	N/A
6	**Were the outcomes of participants included in any comparisons measured in the same way?**					
	**Outcome 1**	All outcomes and list above	☒	☐	☐	☐
		Outcomes such as 30-day mortality, hospital mortality, and bundle adherence were consistently defined and assessed using the same criteria for both groups.				

			Yes	No	Unclear	N/A
7	**Were outcomes measured in a reliable way?**	Outcome defined operationally (ED revisit due to documented treatment failure). Time intervals clearly defined and medians/IQRs reported.				
	**Outcomes**	All outcomes	☒	☐	☐	☐

Bias related to participant retention

			Yes	No	Unclear	N/A
8	**Was follow-up complete and if not, were differences between groups in terms of their follow-up adequately described and analyzed?**					
	**Outcome 1**	The study could not capture revisits to other hospitals — possible outcome under-ascertainment; no explicit follow up completeness data.	**Yes**	**No**	**Unclear**	**N/A**
	Result 1		☐	☐	☒	☐

Statistical Conclusion Validity

9	**Was appropriate statistical analysis used?**					
	**Outcomes**	Fisher's exact for categorical, Mann-Whitney/Wilcoxon for continuous/medians; pre-hoc power calculation reported; p-values presented.	**Yes**	**No**	**Unclear**	**N/A**
	Results		☒	☐	☐	☐

Overall appraisal: **Include:** ☐ **Exclude:** ☐ **Seek Further Info:** ☒

Comments:
This single-center quasi-experimental pre–post study meets several JBI quality items (clearly defined intervention and outcomes, same population/setting, appropriate statistical tests). Main limitations are inherent to non-randomized historical controls: possible confounding (temporal trends, COVID-19 effects), imbalance in sex distribution, potential under-ascertainment of revisits at other hospitals, and lack of multivariable adjustment. On balance the study provides moderate level evidence of an association between the pharmacist-led real-time notification program and reduced ED revisits (plausible process mechanism shown by faster time-to-review). For inclusion

in a meta-analysis, risk-of-bias should be flagged as moderate to serious (quasi-experimental, historical control, potential unmeasured confounding), and sensitivity analyses (exclude / subgroup) are recommended.

Category	Details
Full citation (Vancouver)	Andrade J, Truong J, Ciaramella C. Expansion of a Pharmacist-Led Culture Follow-Up Program to Real-Time Notification of Multidrug-Resistant Microbiology Results in the Emergency Department. Hosp Pharm. 2023.
DOI	https://doi.org/10.1177/00185787231155833
Study design	Single-center quasi-experimental retrospective pre–post study
Setting	The Brooklyn Hospital Center — 42-bed adult Emergency Department (urban community teaching hospital)
Study period	Pre-implementation: Dec 2017 – Mar 2019 (≈15 months); Post-implementation: Apr 2019 – July 2020 (≈15 months)
Population included	Adults (≥18 y) discharged from ED with confirmed positive culture for an MDRO (ESBL, MRSA, VRE) from any site
Initial cohort identified → included in analysis	1,950 patients identified by ED Culture Callback CDTM → 130 patients met inclusion criteria and were analysed (pre n=60; post n=70)
Primary outcome	30-day ED revisit due to antimicrobial treatment failure (lack of resolution or worsening of the same infection)
Key secondary outcomes	EMP interventions (initiate/switch antibiotic, instruct to return to ED, resolution despite inappropriate coverage); time from culture positivity to pharmacist review; antimicrobial susceptibility patterns
Main result (primary)	30-day ED revisit: pre 17/60 (28.3%) vs post 9/70 (12.9%); $p = 0.046$ (statistically significant reduction)
Other key results	Time from culture positive → pharmacist review: median pre 48 (24–72) h vs post 1 (0.2–3) h; $p < 0.001$. EMP interventions overall similar between groups (42.6% vs 35.9%, p=0.484); proportion instructed to return to ED: pre 9/26 (34.6%) vs post 5/28 (17.9%) (not statistically significant).
Main intervention	Policy + workflow change so microbiology notifies EMP (pharmacist) in real time; EMPs (under CDTM) review cultures and can initiate/modify therapy, contact patient, or instruct ED return; 24-hr pharmacy resident on-call supports after hours.
Comparator	Pre-implementation usual care: microbiology notified ED physician (physician responsible for follow up).
Conflicts of interest	One author reported industry presentations; declared in paper.
Authors' main limitations (as reported)	Single center, small sample (n=130), potential for unmeasured confounding, inability to capture revisits to other hospitals, COVID-19 pandemic may have reduced ED volumes during part of post period.

Additional points for reference in appraisal

Were participants included from the same population/setting? | Yes | Both groups sampled from same ED, same inclusion/exclusion criteria, same hospital.

**Was follow up complete and/ or were reasons for loss to follow up reported? | No / Unclear | The study could not capture revisits to other hospitals — possible outcome under-ascertainment; no explicit follow up completeness data.**<br>**Were potential confounding factors identified and accounted for? | Partial / Somewhat | Authors acknowledge several confounders (COVID-19 impact, ability to miss revisits at other hospitals, baseline female sex imbalance); no multivariable adjustment performed — limitation for causal inference.**

© JBI, 2022. All rights reserved. JBI grants use of these tools for research purposes only. All other enquiries should be sent to jbisynthesis@adelaide.edu.au

RoB Assessor: Zainab AlMuhsin	Date of Appraisal: 12/11/2025	Record Number: 10.1016/j.diagmicrobio.2017.10.001
Study Author: Smith JR et al.	**Study Title:** impact of a pharmacist-driven care package on *Staphylococcus aureus* bacteremia management in a large community healthcare network.	Study Year: 2018

	Internal Validity	Choice - Comments/Justification	Yes	No	Unclear	N/A
**Bias related to temporal precedence**						
1	**Is it clear in the study what is the "cause" and what is the "effect" (i.e. there is no confusion about which variable comes first)?**	Cause: pharmacist-led stewardship intervention (SAB care bundle).<br>Effect: improvements in management quality, time to therapy, and clinical outcomes.	☒	☐	☐	☐
**Bias related to selection and allocation**						
2	**Was there a control group?**	Pre-intervention cohort (Oct 2011–Oct 2012) served as control.	☒	☐	☐	☐
**Bias related to confounding factors**						
3	**Were participants included in any comparisons similar?**	Propensity score matching ensured pre- and post-cohorts were similar in baseline demographics, infection type, and MRSA proportion.	☒	☐	☐	☐

Bias related to administration of intervention/exposure

			Yes	No	Unclear	N/A
4	**Were the participants included in any comparisons receiving similar treatment/care, other than the exposure or intervention of interest?**	Standard hospital care protocols unchanged; only addition was pharmacist-driven SAB bundle.	☒	☐	☐	☐

Bias related to assessment, detection and measurement of the outcome

			Yes	No	Unclear	N/A
5	**Were there multiple measurements of the outcome, both pre and post the intervention/exposure?**		**Yes**	**No**	**Unclear**	**N/A**
	**Outcome 1:** All-cause mortality		☒	☐	☐	☐
	**Outcome 2:** Infection resolution / cure		☒	☐	☐	☐
	**Outcome 3:** Time to appropriate therapy		☒	☐	☐	☐
	**Outcome 4:** (ID) consult rate		☒	☐	☐	☐
	**Outcome 5:** Readmission (30 days)		☒	☐	☐	☐
	**Outcome 6**		☐	☐	☐	☐
	**Outcome 7**		☐	☐	☐	☐

6	**Were the outcomes of participants included in any comparisons measured in the same way?**		**Yes**	**No**	**Unclear**	**N/A**

			Yes	No	Unclear	N/A
	**Outcome 1:** All-cause mortality	Mortality defined and recorded using the same hospital EMR criteria in both cohorts.	☒	☐	☐	☐
	**Outcome 2:** Infection resolution / cure	Optimal Care Score applied identically before and after intervention to assess cure and adherence.	☒	☐	☐	☐
	**Outcome 3:** Time to appropriate therapy	Measured as time (hours) from positive blood culture to effective antibiotic in both groups.	☒	☐	☐	☐
	**Outcome 4:** (ID) consult rate	Recorded based on EMR documentation of ID team involvement using same data source.	☒	☐	☐	☐
	**Outcome 5:** Readmission (30 days)	Determined using hospital records for all discharged SAB patients using the same 30-day timeframe.	☒	☐	☐	☐
	**Outcome 6**		☐	☐	☐	☐
	**Outcome 7**		☐	☐	☐	☐

7	**Were outcomes measured in a reliable way?**		**Yes**	**No**	**Unclear**	**N/A**
	**Outcome 1 :** All-cause mortality	Derived from verified hospital electronic medical records; objective outcome with minimal measurement bias.	☒	☐	☐	☐
	**Outcome 2:** Infection resolution / cure	Based on a validated "Optimal Care Score" checklist applied by trained pharmacists and ID specialists.	☒	☐	☐	☐

			Yes	No	Unclear	N/A
	**Outcome 3:** Time to appropriate therapy	Calculated directly from electronic timestamps	☒	☐	☐	☐
	**Outcome 4:** (ID) consult rate	Verified through EMR consult orders and documentation logs.	☒	☐	☐	☐
	**Outcome 5:** Readmission (30 days)	Captured through hospital system tracking all 30-day readmissions.	☒	☐	☐	☐
	**Outcome 6**		☐	☐	☐	☐
	**Outcome 7**		☐	☐	☐	☐

Bias related to participant retention

8	**Was follow-up complete and if not, were differences between groups in terms of their follow-up adequately described and analyzed?**					
	**Outcome 1:** All-cause mortality		**Yes**	**No**	**Unclear**	**N/A**
	Result 1: Mortality rate	All patients in both pre- and post-intervention cohorts were followed until discharge or death; no missing mortality data reported.	☒	☐	☐	☐
	Result 2		☐	☐	☐	☐
	Result 3		☐	☐	☐	☐
	**Outcome 2:** Infection resolution / cure		**Yes**	**No**	**Unclear**	**N/A**
	Result 1: Optimal Care Score		☒	☐	☐	☐

		Yes	No	Unclear	N/A
Result 2		☐	☐	☐	☐
Result 3		☐	☐	☐	☐
**Outcome 3:** Time to appropriate therapy		**Yes**	**No**	**Unclear**	**N/A**
Result 1: Time to appropriate therapy		☒	☐	☐	☐
Result 2		☐	☐	☐	☐
Result 3		☐	☐	☐	☐
**Outcome 4:** (ID) consult rate		**Yes**	**No**	**Unclear**	**N/A**
Result 1: ID consultation status		☒	☐	☐	☐
Result 2		☐	☐	☐	☐
Result 3		☐	☐	☐	☐
**Outcome 5:** Readmission (30 days)		**Yes**	**No**	**Unclear**	**N/A**
Result 1: 30-day readmission		☒	☐	☐	☐
Result 2		☐	☐	☐	☐
Result 3		☐	☐	☐	☐
**Outcome 6**		**Yes**	**No**	**Unclear**	**N/A**
Result 1		☐	☐	☐	☐
Result 2		☐	☐	☐	☐

	Result 3		☐	☐	☐	☐
	**Outcome 7**		**Yes**	**No**	**Unclear**	**N/A**
	Result 1		☐	☐	☐	☐
	Result 2		☐	☐	☐	☐
	Result 3		☐	☐	☐	☐

Statistical Conclusion Validity

**9**	**Was appropriate statistical analysis used?**					
	**Outcome 1:** All-cause mortality		**Yes**	**No**	**Unclear**	**N/A**
	Result 1: Mortality rate	Compared between groups using χ^2 or Fisher’s exact test; *p* values reported.	☒	☐	☐	☐
	Result 2		☐	☐	☐	☐
	Result 3		☐	☐	☐	☐
	**Outcome 2:** Infection resolution / cure		**Yes**	**No**	**Unclear**	**N/A**
	Result 1: Optimal Care Score	Differences in cure rate analyzed with χ^2 test; significant improvement ($p < 0.001$).	☒	☐	☐	☐
	Result 2		☐	☐	☐	☐
	Result 3		☐	☐	☐	☐

		Yes	No	Unclear	N/A
**Outcome 3:** Time to appropriate therapy		Yes	No	Unclear	N/A
Result 1: Time to appropriate therapy	Compared using *t*-test for continuous variable (hours); significant difference ($p < 0.001$).	☒	☐	☐	☐
Result 2		☐	☐	☐	☐
Result 3		☐	☐	☐	☐
**Outcome 4:** (ID) consult rate		Yes	No	Unclear	N/A
Result 1: ID consultation status	Analyzed with χ^2 test; significant improvement from 74% → 100% ($p < 0.001$).	☒	☐	☐	☐
Result 2		☐	☐	☐	☐
Result 3		☐	☐	☐	☐
**Outcome 5:** Readmission (30 days)		Yes	No	Unclear	N/A
Result 1: 30-day readmission	30-day readmission compared using χ^2 test; significant decrease ($p = 0.024$).	☒	☐	☐	☐
Result 2		☐	☐	☐	☐
Result 3		☐	☐	☐	☐
**Outcome 6**		Yes	No	Unclear	N/A
Result 1		☐	☐	☐	☐
Result 2		☐	☐	☐	☐

		Yes	No	Unclear	N/A
Result 3		☐	☐	☐	☐
**Outcome 7**		**Yes**	**No**	**Unclear**	**N/A**
Result 1		☐	☐	☐	☐
Result 2		☐	☐	☐	☐
Result 3		☐	☐	☐	☐

Overall appraisal: **Include:** ☒ **Exclude:** ☐ **Seek Further Info:** ☐

Comments:

Appropriate statistical methods (χ^2, *t*-tests, logistic regression) were applied for all outcomes. Significant improvements were seen in cure rate, time to therapy, ID consults, and readmissions; mortality decreased but was not significant.

Limitations: Single-network design and retrospective data may limit generalizability, though complete follow-up and consistent analysis support strong internal validity.

© JBI, 2022. All rights reserved. JBI grants use of these tools for research purposes only. All other enquiries should be sent to jbisynthesis@adelaide.edu.au

JBI CRITICAL APPRAISAL CHECKLIST FOR COHORT STUDIES

Reviewer:_____Zainab AlMuhsin____________________________________
Date:_________12/11/2025________________________

Author:______Li Z et al____________________________________ Year:__**2017**_______

Record Number:_ **10.1016/j.ajic.2017.02.021**____

	Yes	No	Unclear	Not applicable
1. Were the two groups similar and recruited from the same population?	☐ (highlighted)	☐	☐	☐
2. Were the exposures measured similarly to assign people to both exposed and unexposed groups?	☐ (highlighted)	☐	☐	☐
3. Was the exposure measured in a valid and reliable way?	☐ (highlighted)	☐	☐	☐
4. Were confounding factors identified?	☐ (highlighted)	☐	☐	☐
5. Were strategies to deal with confounding factors stated?	☐ (highlighted)	☐	☐	☐
6. Were the groups/participants free of the outcome at the start of the study (or at the moment of exposure)?	☐ (highlighted)	☐	☐	☐
7. Were the outcomes measured in a valid and reliable way?	☐ (highlighted)	☐	☐	☐
8. Was the follow up time reported and sufficient to be long enough for outcomes to occur?	☐ (highlighted)	☐	☐	☐
9. Was follow up complete, and if not, were the reasons to loss to follow up described and explored?	☐ (highlighted)	☐	☐	☐
10. Were strategies to address incomplete follow up utilized?	☐ (highlighted)	☐	☐	☐
11. Was appropriate statistical analysis used?	☐ (highlighted)	☐	☐	☐

Overall appraisal: Include ☐ (highlighted) Exclude ☐ Seek further info ☐

© JBI, 2020. All rights reserved. JBI grants use of these tools for research purposes only. All other enquiries should be sent to jbisynthesis@adelaide.edu.au.

Comments (Including reason for exclusion)

Strong multicentre cohort showing that pharmacist-driven ASP reduced mortality and MDRO rates. Data collection reliable, analyses appropriate.

Limitations: Short 2-month duration and potential inter-ICU variability may limit generalizability.

© JBI, 2020. All rights reserved. JBI grants use of these tools for research purposes only. All other enquiries should be sent to jbisynthesis@adelaide.edu.au.

EXPLANATION OF COHORT STUDIES CRITICAL APPRAISAL

How to Cite: *Moola S, Munn Z, Tufanaru C, Aromataris E, Sears K, Sfetcu R, Currie M, Qureshi R, Mattis P, Lisy K, Mu P-F. Chapter 7: Systematic reviews of etiology and risk . In: Aromataris E, Munn Z (Editors). JBI Manual for Evidence Synthesis.* JBI, 2020. Available from https://synthesismanual.jbi.global

Cohort Studies Critical Appraisal Tool

Answers: Yes, No, Unclear or Not/Applicable

1. Were the two groups similar and recruited from the same population?

Check the paper carefully for descriptions of participants to determine if patients within and across groups have similar characteristics in relation to exposure (e.g. risk factor under investigation). The two groups selected for comparison should be as similar as possible in all characteristics except for their exposure status, relevant to the study in question. The authors should provide clear inclusion and exclusion criteria that they developed prior to recruitment of the study participants.

2. Were the exposures measured similarly to assign people to both exposed and unexposed groups?

A high quality study at the level of cohort design should mention or describe how the exposures were measured. The exposure measures should be clearly defined and described in detail. This will enable reviewers to assess whether or not the participants received the exposure of interest.

3. Was the exposure measured in a valid and reliable way?

The study should clearly describe the method of measurement of exposure. Assessing validity requires that a 'gold standard' is available to which the measure can be compared. The validity of exposure measurement usually relates to whether a current measure is appropriate or whether a measure of past exposure is needed.

Reliability refers to the processes included in an epidemiological study to check repeatability of measurements of the exposures. These usually include intra-observer reliability and inter-observer reliability.

4. Were confounding factors identified?

Confounding has occurred where the estimated intervention exposure effect is biased by the presence of some difference between the comparison groups (apart from the exposure investigated/of interest). Typical confounders include baseline characteristics, prognostic factors, or concomitant exposures (e.g. smoking). A confounder is a difference between the comparison groups and it influences the direction of the study results. A high quality study at the level of cohort design will identify the potential confounders and measure them (where possible). This is difficult for studies where behavioral, attitudinal or lifestyle factors may impact on the results.

© JBI, 2020. All rights reserved. JBI grants use of these tools for research purposes only. All other enquiries should be sent to jbisynthesis@adelaide.edu.au.

5. Were strategies to deal with confounding factors stated?

Strategies to deal with effects of confounding factors may be dealt within the study design or in data analysis. By matching or stratifying sampling of participants, effects of confounding factors can be adjusted for. When dealing with adjustment in data analysis, assess the statistics used in the study. Most will be some form of multivariate regression analysis to account for the confounding factors measured. Look out for a description of statistical methods as regression methods such as logistic regression are usually employed to deal with confounding factors/variables of interest.

6. Were the groups/participants free of the outcome at the start of the study (or at the moment of exposure)?

The participants should be free of the outcomes of interest at the start of the study. Refer to the 'methods' section in the paper for this information, which is usually found in descriptions of participant/sample recruitment, definitions of variables, and/or inclusion/exclusion criteria.

7. Were the outcomes measured in a valid and reliable way?

Read the methods section of the paper. If for e.g. lung cancer is assessed based on existing definitions or diagnostic criteria, then the answer to this question is likely to be yes. If lung cancer is assessed using observer reported, or self-reported scales, the risk of over- or under-reporting is increased, and objectivity is compromised. Importantly, determine if the measurement tools used were validated instruments as this has a significant impact on outcome assessment validity.

Having established the objectivity of the outcome measurement (e.g. lung cancer) instrument, it's important to establish how the measurement was conducted. Were those involved in collecting data trained or educated in the use of the instrument/s? (e.g. radiographers). If there was more than one data collector, were they similar in terms of level of education, clinical or research experience, or level of responsibility in the piece of research being appraised?

8. Was the follow up time reported and sufficient to be long enough for outcomes to occur?

The appropriate length of time for follow up will vary with the nature and characteristics of the population of interest and/or the intervention, disease or exposure. To estimate an appropriate duration of follow up, read across multiple papers and take note of the range for duration of follow up. The opinions of experts in clinical practice or clinical research may also assist in determining an appropriate duration of follow up. For example, a longer timeframe may be needed to examine the association between occupational exposure to asbestos and the risk of lung cancer. It is important, particularly in cohort studies that follow up is long enough to enable the outcomes. However, it should be remembered that the research question and outcomes being examined would probably dictate the follow up time.

© JBI, 2020. All rights reserved. JBI grants use of these tools for research purposes only. All other enquiries should be sent to jbisynthesis@adelaide.edu.au.

9. Was follow up complete, and if not, were the reasons to loss to follow up described and explored?

It is important in a cohort study that a greater percentage of people are followed up. As a general guideline, at least 80% of patients should be followed up. Generally a dropout rate of 5% or less is considered insignificant. A rate of 20% or greater is considered to significantly impact on the validity of the study. However, in observational studies conducted over a lengthy period of time a higher dropout rate is to be expected. A decision on whether to include or exclude a study because of a high dropout rate is a matter of judgement based on the reasons why people dropped out, and whether dropout rates were comparable in the exposed and unexposed groups.

Reporting of efforts to follow up participants that dropped out may be regarded as an indicator of a well conducted study. Look for clear and justifiable description of why people were left out, excluded, dropped out etc. If there is no clear description or a statement in this regards, this will be a 'No'.

10. Were strategies to address incomplete follow up utilized?

Some people may withdraw due to change in employment or some may die; however, it is important that their outcomes are assessed. Selection bias may occur as a result of incomplete follow up. Therefore, participants with unequal follow up periods must be taken into account in the analysis, which should be adjusted to allow for differences in length of follow up periods. This is usually done by calculating rates which use person-years at risk, i.e. considering time in the denominator.

11. Was appropriate statistical analysis used?

As with any consideration of statistical analysis, consideration should be given to whether there was a more appropriate alternate statistical method that could have been used. The methods section of cohort studies should be detailed enough for reviewers to identify which analytical techniques were used (in particular, regression or stratification) and how specific confounders were measured.

For studies utilizing regression analysis, it is useful to identify if the study identified which variables were included and how they related to the outcome. If stratification was the analytical approach used, were the strata of analysis defined by the specified variables? Additionally, it is also important to assess the appropriateness of the analytical strategy in terms of the assumptions associated with the approach as differing methods of analysis are based on differing assumptions about the data and how it will respond.

© JBI, 2020. All rights reserved. JBI grants use of these tools for research purposes only. All other enquiries should be sent to jbisynthesis@adelaide.edu.au.

RoB Assessor: Mouda Hamdan	Date of Appraisal: 17/11/2025	Record Number: 28
Study Author: Mahrous et al	Study Title: Clinical impact of pharmacist-directed antimicrobial stewardship guidance following bloc culture rapid diagnostic testing	Study Year: 2020

Internal Validity		Choice - Comments/Justification	Yes	No	Unclear	N/A
**Bias related to temporal precedence**						
1	Is it clear in the study what is the “cause” and what is the “effect” (i.e. there is no confusion about which variable comes first)?		☒	☐	☐	☐
**Bias related to selection and allocation**						
2	Was there a control group?		☒	☐	☐	☐
**Bias related to confounding factors**						
3	Were participants included in any comparisons similar?		☒	☐	☐	☐
**Bias related to administration of intervention/exposure**						
4	Were the participants included in any comparisons receiving similar treatment/care, other than the exposure or intervention of interest?		☒	☐	☐	☐

Bias related to assessment, detection and measurement of the outcome

5	Were there multiple measurements of the outcome, both pre and post the intervention/exposure?		Yes	No	Unclear	N/A
	**Outcome 1**	Mortality	☒	☐	☐	☐
	**Outcome 2**	Hospital length of stay	☒	☐	☐	☐
	**Outcome 3**	ICU length of stay	☒	☐	☐	☐
	**Outcome 4**	Time to appropriate therapy	☒	☐	☐	☐
	**Outcome 5**	De-escalation	☒	☐	☐	☐
	**Outcome 6**	Escalation	☒	☐	☐	☐
	**Outcome 7**	readmission	☒	☐	☐	☐

6	Were the outcomes of participants included in any comparisons measured in the same way?		Yes	No	Unclear	N/A
	**Outcome 1**	Mortality	☒	☐	☐	☐
	**Outcome 2**	Hospital length of stay	☒	☐	☐	☐
	**Outcome 3**	ICU length of stay	☒	☐	☐	☐
	**Outcome 4**	Time to appropriate therapy	☒	☐	☐	☐
	**Outcome 5**	De-escalation	☒	☐	☐	☐
	**Outcome 6**	Escalation	☒	☐	☐	☐

			Yes	No	Unclear	N/A
	**Outcome 7**	readmission	☒	☐	☐	☐

7	**Were outcomes measured in a reliable way?**		**Yes**	**No**	**Unclear**	**N/A**
	**Outcome 1**	Mortality	☒	☐	☐	☐
	**Outcome 2**	Hospital length of stay	☒	☐	☐	☐
	**Outcome 3**	ICU length of stay	☒	☐	☐	☐
	**Outcome 4**	Time to appropriate therapy	☒	☐	☐	☐
	**Outcome 5**	De-escalation	☒	☐	☐	☐
	**Outcome 6**	Escalation	☒	☐	☐	☐
	**Outcome 7**	readmission	☒	☐	☐	☐

Bias related to participant retention

8	**Was follow-up complete and if not, were differences between groups in terms of their follow-up adequately described and analyzed?**					
	**Outcome 1**	Mortality	**Yes**	**No**	**Unclear**	**N/A**
	Result 1	N (%): Pre: 30 (18%), Post: 14 (10%)	☒	☐	☐	☐
	Result 2		☐	☐	☐	☐

		Yes	No	Unclear	N/A
Result 3		☐	☐	☐	☐
**Outcome 2**	Hospital LOS	**Yes**	**No**	**Unclear**	**N/A**
Result 1	Median (IQR): Pre: 8 (5–15), Post: 6.5 (4–12)	☒	☐	☐	☐
Result 2	MRSA: pre: 13.5 (7.25-19), post 9.5 (8.25-12.75)	☒	☐	☐	☐
Result 3		☐	☐	☐	☐
**Outcome 3**	ICU LOS	**Yes**	**No**	**Unclear**	**N/A**
Result 1	Median (IQR): Pre: 6 (4–12), Post: 4 (2–8)	☒	☐	☐	☐
Result 2		☐	☐	☐	☐
Result 3		☐	☐	☐	☐
**Outcome 4**	Time to appropriate therapy	**Yes**	**No**	**Unclear**	**N/A**
Result 1	Median (IQR): Pre: 41 (27.7–64), Post: 18.1 (8.7–27)	☒	☐	☐	☐
Result 2	MRSA: pre: 39 (28.75-52.5), post 9 (0.4-18)	☒	☐	☐	☐
Result 3		☐	☐	☐	☐
**Outcome 5**	De-escalation	**Yes**	**No**	**Unclear**	**N/A**
Result 1	N (%): Pre: 41 (25%), Post: 31 (21%)	☒	☐	☐	☐
Result 2		☐	☐	☐	☐
Result 3		☐	☐	☐	☐

Outcome 6	escalation	Yes	No	Unclear	N/A
Result 1	N (%): Pre: 23 (14%), Post: 13 (9%)	☒	☐	☐	☐
Result 2		☐	☐	☐	☐
Result 3		☐	☐	☐	☐
**Outcome 7**	readmission	**Yes**	**No**	**Unclear**	**N/A**
Result 1	N (%): Pre: 40 (24%), Post: 17 (11%)	☒	☐	☐	☐
Result 2		☐	☐	☐	☐
Result 3		☐	☐	☐	☐

Statistical Conclusion Validity

9	Was appropriate statistical analysis used?					
	**Outcome 1**	Mortality	**Yes**	**No**	**Unclear**	**N/A**
	Result 1	N (%): Pre: 30 (18%), Post: 14 (10%)	☒	☐	☐	☐
	Result 2		☐	☐	☐	☐
	Result 3		☐	☐	☐	☐
	**Outcome 2**	Hospital LOS	**Yes**	**No**	**Unclear**	**N/A**
	Result 1	Median (IQR): Pre: 8 (5–15), Post: 6.5 (4–12)	☒	☐	☐	☐

		Yes	No	Unclear	N/A
Result 2	MRSA: pre: 13.5 (7.25-19), post 9.5 (8.25-12.75)	☒	☐	☐	☐
Result 3		☐	☐	☐	☐
**Outcome 3**	ICU LOS	**Yes**	**No**	**Unclear**	**N/A**
Result 1	Median (IQR): Pre: 6 (4–12), Post: 4 (2–8)	☒	☐	☐	☐
Result 2		☐	☐	☐	☐
Result 3		☐	☐	☐	☐
**Outcome 4**	Time to appropriate therapy	**Yes**	**No**	**Unclear**	**N/A**
Result 1	Median (IQR): Pre: 41 (27.7–64), Post: 18.1 (8.7–27)	☒	☐	☐	☐
Result 2	MRSA: pre: 39 (28.75-52.5), post 9 (0.4-18)	☒	☐	☐	☐
Result 3		☐	☐	☐	☐
**Outcome 5**	De-escalation	**Yes**	**No**	**Unclear**	**N/A**
Result 1	N (%): Pre: 41 (25%), Post: 31 (21%)	☒	☐	☐	☐
Result 2		☐	☐	☐	☐
Result 3		☐	☐	☐	☐
**Outcome 6**	escalation	**Yes**	**No**	**Unclear**	**N/A**
Result 1	N (%): Pre: 23 (14%), Post: 13 (9%)	☒	☐	☐	☐
Result 2		☐	☐	☐	☐

		Yes	No	Unclear	N/A
Result 3		☐	☐	☐	☐
**Outcome 7**	Readmission	**Yes**	**No**	**Unclear**	**N/A**
Result 1	N (%): Pre: 40 (24%), Post: 17 (11%)	☒	☐	☐	☐
Result 2		☐	☐	☐	☐
Result 3		☐	☐	☐	☐

Overall appraisal: **Include:** ☒ **Exclude:** ☐ **Seek Further Info:** ☐

Comments:

© JBI, 2022. All rights reserved. JBI grants use of these tools for research purposes only. All other enquiries should be sent to jbisynthesis@adelaide.edu.au

RoB Assessor: Mouda Hamdan	Date of Appraisal: 17/11/2025	Record Number: 29
Study Author: Sadyrbaeva-Dolgova et al	Study Title: Pharmacist recommendations for carbapenem de-escalation in urinary tract infectio within an antimicrobial stewardship program	Study Year: 2019

	Internal Validity	Choice - Comments/Justification	Yes	No	Unclear	N/A
**Bias related to temporal precedence**						
1	Is it clear in the study what is the "cause" and what is the "effect" (i.e. there is no confusion about which variable comes first)?		☒	☐	☐	☐
**Bias related to selection and allocation**						
2	Was there a control group?		☒	☐	☐	☐
**Bias related to confounding factors**						
3	Were participants included in any comparisons similar?		☐	☒	☐	☐
**Bias related to administration of intervention/exposure**						
4	Were the participants included in any comparisons receiving similar treatment/care, other than the exposure or intervention of interest?		☒	☐	☐	☐

Bias related to assessment, detection and measurement of the outcome

5	Were there multiple measurements of the outcome, both pre and post the intervention/exposure?		Yes	No	Unclear	N/A
	Outcome 1	In-hospital mortality	☒	☐	☐	☐
	Outcome 2	Hospital length of stay	☒	☐	☐	☐
	Outcome 3	Overall duration of antibiotic therapy	☒	☐	☐	☐
	Outcome 4	Overall duration of Carbapenem therapy	☒	☐	☐	☐
	Outcome 5	De-escalation rate	☒	☐	☐	☐
	Outcome 6	Readmission rate	☒	☐	☐	☐
	Outcome 7		☐	☐	☐	☐

6	Were the outcomes of participants included in any comparisons measured in the same way?		Yes	No	Unclear	N/A
	Outcome 1	In-hospital mortality	☒	☐	☐	☐
	Outcome 2	Hospital length of stay	☒	☐	☐	☐
	Outcome 3	Overall duration of antibiotic therapy	☒	☐	☐	☐
	Outcome 4	Overall duration of Carbapenem therapy	☒	☐	☐	☐
	Outcome 5	De-escalation rate	☒	☐	☐	☐
	Outcome 6	Readmission rate	☒	☐	☐	☐

			Yes	No	Unclear	N/A
	**Outcome 7**		☐	☐	☐	☐

7	**Were outcomes measured in a reliable way?**		**Yes**	**No**	**Unclear**	**N/A**
	**Outcome 1**	In-hospital mortality	☒	☐	☐	☐
	**Outcome 2**	Hospital length of stay	☒	☐	☐	☐
	**Outcome 3**	Overall duration of antibiotic therapy	☒	☐	☐	☐
	**Outcome 4**	Overall duration of Carbapenem therapy	☒	☐	☐	☐
	**Outcome 5**	de-escalation rate	☒	☐	☐	☐
	**Outcome 6**	Readmission rate	☒	☐	☐	☐
	**Outcome 7**		☐	☐	☐	☐

Bias related to participant retention

8	**Was follow-up complete and if not, were differences between groups in terms of their follow-up adequately described and analyzed?**					
	**Outcome 1**	In-hospital mortality	**Yes**	**No**	**Unclear**	**N/A**
	Result 1	n (%): de-escalation 6 (7.4), escalation 24 (29.3).	☒	☐	☐	☐

Result 2	MDROs, OR (95% CI): 0.85 (0.34–2.16)	☒	☐	☐	☐
Result 3		☐	☐	☐	☐
**Outcome 2**	Hospital length of stay	**Yes**	**No**	**Unclear**	**N/A**
Result 1	median (IQR): de-escalation 9 (7–23), no de-escalation 14 (8–30)	☒	☐	☐	☐
Result 2		☐	☐	☐	☐
Result 3		☐	☐	☐	☐
**Outcome 3**	Overall duration of antibiotic therapy	**Yes**	**No**	**Unclear**	**N/A**
Result 1	median (days) de-escalation 13 (10–17), no de-escalation 9 (6–13)	☒	☐	☐	☐
Result 2		☐	☐	☐	☐
Result 3		☐	☐	☐	☐
**Outcome 4**	Overall duration of Carbapenem therapy	**Yes**	**No**	**Unclear**	**N/A**
Result 1	de-escalation 4 (2-6), no de-escalation 6 (4–10)	☒	☐	☐	☐
Result 2		☐	☐	☐	☐
Result 3		☐	☐	☐	☐
**Outcome 5**	De-escalation rate	**Yes**	**No**	**Unclear**	**N/A**
Result 1	Effect of MDROs on de-escalation, OR (95%CI): 0.40 (0.19–0.84). adjusted OR (95%CI): 0.33 (0.15–0.74)	☒	☐	☐	☐

			Yes	No	Unclear	N/A
	Result 2		☐	☐	☐	☐
	Result 3		☐	☐	☐	☐
	**Outcome 6**	30-day readmission	**Yes**	**No**	**Unclear**	**N/A**
	Result 1	n (%): de-escalation 16 (19.8), escalation 12 (14.6)	☒	☐	☐	☐
	Result 2		☐	☐	☐	☐
	Result 3		☐	☐	☐	☐
	**Outcome 7**		**Yes**	**No**	**Unclear**	**N/A**
	Result 1		☐	☐	☐	☐
	Result 2		☐	☐	☐	☐
	Result 3		☐	☐	☐	☐

Statistical Conclusion Validity

9	**Was appropriate statistical analysis used?**					
	**Outcome 1**	In-hospital mortality	**Yes**	**No**	**Unclear**	**N/A**
	Result 1	n (%): de-escalation 6 (7.4), escalation 24 (29.3).	☒	☐	☐	☐
	Result 2	MDROs, OR (95% CI): 0.85 (0.34–2.16)	☒	☐	☐	☐
	Result 3		☐	☐	☐	☐

Outcome 2	Hospital length of stay	Yes	No	Unclear	N/A
Result 1	median (IQR): de-escalation 9 (7–23), no de-escalation 14 (8–30)	☒	☐	☐	☐
Result 2		☐	☐	☐	☐
Result 3		☐	☐	☐	☐
**Outcome 3**	Overall duration of antibiotic therapy	**Yes**	**No**	**Unclear**	**N/A**
Result 1	median (days) de-escalation 13 (10–17), no de-escalation 9 (6–13)	☒	☐	☐	☐
Result 2		☐	☐	☐	☐
Result 3		☐	☐	☐	☐
**Outcome 4**	Overall duration of Carbapenem therapy	**Yes**	**No**	**Unclear**	**N/A**
Result 1	de-escalation 4 (2-6), no de-escalation 6 (4–10)	☒	☐	☐	☐
Result 2		☐	☐	☐	☐
Result 3		☐	☐	☐	☐
**Outcome 5**	De-escalation rate	**Yes**	**No**	**Unclear**	**N/A**
Result 1	Effect of MDROs on de-escalation, OR (95%CI): 0.40 (0.19–0.84). adjusted OR(95%CI): 0.33 (0.15–0.74)	☒	☐	☐	☐
Result 2		☐	☐	☐	☐

		Yes	No	Unclear	N/A
Result 3		☐	☐	☐	☐
**Outcome 6**	30-day readmission	**Yes**	**No**	**Unclear**	**N/A**
Result 1	n (%): de-escalation 16 (19.8), escalation 12 (14.6)	☒	☐	☐	☐
Result 2		☐	☐	☐	☐
Result 3		☐	☐	☐	☐
**Outcome 7**		**Yes**	**No**	**Unclear**	**N/A**
Result 1		☐	☐	☐	☐
Result 2		☐	☐	☐	☐
Result 3		☐	☐	☐	☐

Overall appraisal: **Include:** ☒ **Exclude:** ☐ **Seek Further Info:** ☐

Comments:

RoB Assessor: Mouda Hamdan	Date of Appraisal: 15/11/2025	Record Number: 2
Study Author: Knudsen & Andersen	Study Title: A Multidisciplinary Intervention to Reduce Infections of ESBL- and AmpC-Producing, Gram-Negative Bacteria at a University Hospital	Study Year: 2014

Internal Validity		Choice - Comments/Justification	Yes	No	Unclear	N/A
**Bias related to temporal precedence**						
1	Is it clear in the study what is the "cause" and what is the "effect" (i.e. there is no confusion about which variable comes first)?		☒	☐	☐	☐
**Bias related to selection and allocation**						
2	Was there a control group?		☒	☐	☐	☐
**Bias related to confounding factors**						
3	Were participants included in any comparisons similar?		☒	☐	☐	☐
**Bias related to administration of intervention/exposure**						
4	Were the participants included in any comparisons receiving similar treatment/care, other than the exposure or intervention of interest?		☒	☐	☐	☐

Bias related to assessment, detection and measurement of the outcome

5	Were there multiple measurements of the outcome, both pre and post the intervention/exposure?		Yes	No	Unclear	N/A
	Outcome 1	Antibiotic consumption	☒	☐	☐	☐
	Outcome 2	30-day bacteraemia mortality	☒	☐	☐	☐
	Outcome 3		☐	☐	☐	☐
	Outcome 4		☐	☐	☐	☐
	Outcome 5		☐	☐	☐	☐
	Outcome 6		☐	☐	☐	☐
	Outcome 7		☐	☐	☐	☐

6	Were the outcomes of participants included in any comparisons measured in the same way?		Yes	No	Unclear	N/A
	Outcome 1	Antibiotic consumption	☒	☐	☐	☐
	Outcome 2	30-day bacteraemia mortality	☒	☐	☐	☐
	Outcome 3		☐	☐	☐	☐
	Outcome 4		☐	☐	☐	☐
	Outcome 5		☐	☐	☐	☐

			Yes	No	Unclear	N/A
	**Outcome 6**		☐	☐	☐	☐
	**Outcome 7**		☐	☐	☐	☐

7	**Were outcomes measured in a reliable way?**		**Yes**	**No**	**Unclear**	**N/A**
	**Outcome 1**	Antibiotic consumption	☒	☐	☐	☐
	**Outcome 2**	30-day bacteraemia mortality	☒	☐	☐	☐
	**Outcome 3**		☐	☐	☐	☐
	**Outcome 4**		☐	☐	☐	☐
	**Outcome 5**		☐	☐	☐	☐
	**Outcome 6**		☐	☐	☐	☐
	**Outcome 7**		☐	☐	☐	☐

Bias related to participant retention

8	**Was follow-up complete and if not, were differences between groups in terms of their follow-up adequately described and analyzed?**					
	**Outcome 1**	Antibiotic consumption	**Yes**	**No**	**Unclear**	**N/A**

Result 1	cefuroxime (2143.8 DDD per 1000 OBD per month, p,0.0001), ertapenem (+15.1 DDD per 1000 OBD per month, p,0.0001), beta-lactamase sensitive penicillins (phenoxy- and benzyl-penicillins) (+35.8 DDD per 1000 OBD per month, p = 0.005), and piperacillin-tazobactam (+91.1 DDD per 1000 OBD per month, p,0.0001)	☒	☐	☐	☐
Result 2		☐	☐	☐	☐
Result 3		☐	☐	☐	☐
**Outcome 2**	30-day bacteraemia mortality	**Yes**	**No**	**Unclear**	**N/A**
Result 1	Pre-AMS 5% (11 of 34 patients died)vs post-AMS 17% (5 of 29) in 2010–2011 (p = 0.25)	☒	☐	☐	☐
Result 2		☐	☐	☐	☐
Result 3		☐	☐	☐	☐
**Outcome 3**		**Yes**	**No**	**Unclear**	**N/A**
Result 1		☐	☐	☐	☐
Result 2		☐	☐	☐	☐
Result 3		☐	☐	☐	☐
**Outcome 4**		**Yes**	**No**	**Unclear**	**N/A**
Result 1		☐	☐	☐	☐
Result 2		☐	☐	☐	☐

Result 3		☐	☐	☐	☐
**Outcome 5**		**Yes**	**No**	**Unclear**	**N/A**
Result 1		☐	☐	☐	☐
Result 2		☐	☐	☐	☐
Result 3		☐	☐	☐	☐
**Outcome 6**		**Yes**	**No**	**Unclear**	**N/A**
Result 1		☐	☐	☐	☐
Result 2		☐	☐	☐	☐
Result 3		☐	☐	☐	☐
**Outcome 7**		**Yes**	**No**	**Unclear**	**N/A**
Result 1		☐	☐	☐	☐
Result 2		☐	☐	☐	☐
Result 3		☐	☐	☐	☐

Statistical Conclusion Validity

9	**Was appropriate statistical analysis used?**					
	**Outcome 1**	Antibiotic consumption	**Yes**	**No**	**Unclear**	**N/A**

Result 1	cefuroxime (2143.8 DDD per 1000 OBD per month, p,0.0001), ertapenem (+15.1 DDD per 1000 OBD per month, p,0.0001), beta-lactamase sensitive penicillins (phenoxy- and benzyl-penicillins) (+35.8 DDD per 1000 OBD per month, p = 0.005), and piperacillin-tazobactam (+91.1 DDD per 1000 OBD per month, p,0.0001)	☒	☐	☐	☐
Result 2		☐	☐	☐	☐
Result 3		☐	☐	☐	☐
**Outcome 2**	30-day bacteraemia mortality	**Yes**	**No**	**Unclear**	**N/A**
Result 1	Pre-AMS 5% (11 of 34 patients died)vs post-AMS 17% (5 of 29) in 2010–2011 (p = 0.25)	☒	☐	☐	☐
Result 2		☐	☐	☐	☐
Result 3		☐	☐	☐	☐
**Outcome 3**		**Yes**	**No**	**Unclear**	**N/A**
Result 1		☐	☐	☐	☐
Result 2		☐	☐	☐	☐
Result 3		☐	☐	☐	☐
**Outcome 4**		**Yes**	**No**	**Unclear**	**N/A**
Result 1		☐	☐	☐	☐
Result 2		☐	☐	☐	☐

		Yes	No	Unclear	N/A
Result 3		☐	☐	☐	☐
**Outcome 5**		**Yes**	**No**	**Unclear**	**N/A**
Result 1		☐	☐	☐	☐
Result 2		☐	☐	☐	☐
Result 3		☐	☐	☐	☐
**Outcome 6**		**Yes**	**No**	**Unclear**	**N/A**
Result 1		☐	☐	☐	☐
Result 2		☐	☐	☐	☐
Result 3		☐	☐	☐	☐
**Outcome 7**		**Yes**	**No**	**Unclear**	**N/A**
Result 1		☐	☐	☐	☐
Result 2		☐	☐	☐	☐
Result 3		☐	☐	☐	☐

Overall appraisal: **Include:** ☒ **Exclude:** ☐ **Seek Further Info:** ☐

Comments:

© JBI, 2022. All rights reserved. JBI grants use of these tools for research purposes only. All other enquiries should be sent to jbisynthesis@adelaide.edu.au

RoB Assessor: Zainab AlMuhsin	Date of Appraisal: 15/11/2025	Record Number: 10.1186/s13613-024-01271-9
**Study Author:** Rinaldi et al.	**Study Title:** Impact of a multidisciplinary management team on clinical outcome in ICU patients affected by Gram-negative bloodstream infections.	**Study Year: 2024**

	Internal Validity	Choice - Comments/Justification	Yes	No	Unclear	N/A
**Bias related to temporal precedence**						
1	**Is it clear in the study what is the "cause" and what is the "effect" (i.e. there is no confusion about which variable comes first)?**	Cause: implementing a multidisciplinary ICU management team.<br>Effect: changes in empirical appropriateness, cure rates, microbiological failure, MDRO colonization, and antibiotic metrics.	☒	☐	☐	☐
**Bias related to selection and allocation**						
2	**Was there a control group?**	Pre-intervention patients from 2018 served as the comparison group. (Historical control)	☒	☐	☐	☐
**Bias related to confounding factors**						
3	**Were participants included in any comparisons similar?**	ICU Gram-negative BSI patients were similar in demographics, severity, infection source, and MDRO colonization between pre and post groups.	☒	☐	☐	☐

Bias related to administration of intervention/exposure

			Yes	No	Unclear	N/A
4	**Were the participants included in any comparisons receiving similar treatment/care, other than the exposure or intervention of interest?**	Both periods used the same hospital, ICU protocols, and diagnostic methods; only the multidisciplinary stewardship model changed.	☒	☐	☐	☐

Bias related to assessment, detection and measurement of the outcome

			Yes	No	Unclear	N/A
5	**Were there multiple measurements of the outcome, both pre and post the intervention/exposure?**					
	**Outcome 1:** Appropriate empirical therapy	Appropriateness of empiric therapy was assessed in both pre and post groups using the same clinical criteria.	☒	☐	☐	☐
	**Outcome 2:** Clinical cure at 7 days	Day-7 clinical cure was evaluated in all patients in both phases.	☒	☐	☐	☐
	**Outcome 3:** 30-day mortality	30-day mortality was recorded for all patients in both pre and post intervention periods.	☒	☐	☐	☐
	**Outcome 4:** Meropenem consumption (DDD)	Meropenem DDD was measured throughout both the pre and post intervention years.	☒	☐	☐	☐
	**Outcome 5:** Microbiological failure	Persistent, breakthrough, or recurrent bacteremia was assessed in both groups.	☒	☐	☐	☐
	**Outcome 6:** New 30-day MDRO colonization	New colonization within 30 days was tracked in both pre and post periods using the same surveillance.	☒	☐	☐	☐

			Yes	No	Unclear	N/A
	**Outcome 7**		☐	☐	☐	☐

6	**Were the outcomes of participants included in any comparisons measured in the same way?**		**Yes**	**No**	**Unclear**	**N/A**
	**Outcome 1:** Appropriate empirical therapy	Appropriateness was defined using the same criteria and guideline-based standards in both pre and post groups.	☒	☐	☐	☐
	**Outcome 2:** Clinical cure at 7 days	Day-7 clinical cure was assessed using the same clinical definitions and documentation procedures in both periods.	☒	☐	☐	☐
	**Outcome 3:** 30-day mortality	Mortality data were obtained from the same hospital records with identical coding across both phases.	☒	☐	☐	☐
	**Outcome 4:** Meropenem consumption (DDD)	DDD was calculated using the same WHO ATC/DDD methodology and pharmacy database in pre and post periods.	☒	☐	☐	☐
	**Outcome 5:** Microbiological failure	Definitions for persistent, breakthrough, and recurrent bacteremia were consistent across both groups and based on the same microbiology processes.	☒	☐	☐	☐
	**Outcome 6:** New 30-day MDRO colonization	Colonization surveillance followed the same screening protocol, lab methods, and definitions before and after the intervention.	☒	☐	☐	☐

			Yes	No	Unclear	N/A
	**Outcome 7**		☐	☐	☐	☐

7	**Were outcomes measured in a reliable way?**		**Yes**	**No**	**Unclear**	**N/A**
	**Outcome 1:** Appropriate empirical therapy	Appropriateness was assessed using guideline-based criteria and verified through EMR documentation, ensuring high reliability.	☒	☐	☐	☐
	**Outcome 2:** Clinical cure at 7 days	Cure status was determined from objective clinical documentation (vital signs, labs, symptoms) recorded consistently in the EMR.	☒	☐	☐	☐
	**Outcome 3:** 30-day mortality	Mortality data came from hospital administrative records, which are standardized and routinely validated.	☒	☐	☐	☐
	**Outcome 4:** Meropenem consumption (DDD)	DDD was calculated using validated WHO ATC/DDD methodology from pharmacy dispensing logs.	☒	☐	☐	☐
	**Outcome 5:** Microbiological failure	Failure (persistent, breakthrough, recurrent bacteremia) was measured using standardized microbiology culture methods and timed blood culture results.	☒	☐	☐	☐
	**Outcome 6:** New 30-day MDRO colonization	Colonization was identified via routine surveillance swabs and accredited laboratory procedures, ensuring reliable detection.	☒	☐	☐	☐
	**Outcome 7**		☐	☐	☐	☐

Bias related to participant retention

			Yes	No	Unclear	N/A
8	**Was follow-up complete and if not, were differences between groups in terms of their follow-up adequately described and analyzed?**					
	**Outcome 1:** Appropriate empirical therapy		Yes	No	Unclear	N/A
	Result 1: 55.2% (pre) → 86.9% (post), *p < 0.001*	All patients in both phases had empiric therapy assessed; no missing data.	☒	☐	☐	☐
	Result 2		☐	☐	☐	☐
	Result 3		☐	☐	☐	☐
	**Outcome 2:** Clinical cure at 7 days		Yes	No	Unclear	N/A
	Result 1: 47.7% (pre) → 75% (post), *p < 0.001*	All patients were evaluated for day-7 cure; full follow-up reported.	☒	☐	☐	☐
	Result 2		☐	☐	☐	☐
	Result 3		☐	☐	☐	☐
	**Outcome 3:** 30-day mortality		Yes	No	Unclear	N/A
	Result 1: 35.8% (pre) → 35.3% (post), NS	30-day mortality available for all patients; no losses.	☒	☐	☐	☐
	Result 2		☐	☐	☐	☐

		Yes	No	Unclear	N/A
Result 3		☐	☐	☐	☐
**Outcome 4:** Meropenem consumption (DDD)		**Yes**	**No**	**Unclear**	**N/A**
Result 1: 585 DDD → 232 DDD, *p* < *0.001*	Pharmacy DDD data were complete for the entire pre and post periods.	☒	☐	☐	☐
Result 2		☐	☐	☐	☐
Result 3		☐	☐	☐	☐
**Outcome 5:** Microbiological failure		**Yes**	**No**	**Unclear**	**N/A**
Result 1: 29.9% (pre) → 10.3% (post), *p* = *0.005*	All patients had follow-up cultures; no cases lost.	☒	☐	☐	☐
Result 2		☐	☐	☐	☐
Result 3		☐	☐	☐	☐
**Outcome 6:** New 30-day MDRO colonization		**Yes**	**No**	**Unclear**	**N/A**
Result 1: 36.6% (pre) → 8.3% (post), *p* < *0.001*	All ICU patients underwent colonization screening; complete dataset.	☒	☐	☐	☐
Result 2		☐	☐	☐	☐
Result 3		☐	☐	☐	☐
**Outcome 7**		**Yes**	**No**	**Unclear**	**N/A**
Result 1		☐	☐	☐	☐

			Yes	No	Unclear	N/A
	Result 2		☐	☐	☐	☐
	Result 3		☐	☐	☐	☐

Statistical Conclusion Validity

9	**Was appropriate statistical analysis used?**					
	**Outcome 1:** Appropriate empirical therapy		**Yes**	**No**	**Unclear**	**N/A**
	Result 1: 55.2% (pre) → 86.9% (post), $p < 0.001$	A χ^2 test was correctly used for comparing proportions between groups.	☒	☐	☐	☐
	Result 2		☐	☐	☐	☐
	Result 3		☐	☐	☐	☐
	**Outcome 2:** Clinical cure at 7 days		**Yes**	**No**	**Unclear**	**N/A**
	Result 1: 47.7% (pre) → 75% (post), $p < 0.001$	A χ^2 test for categorical outcomes was appropriate and correctly applied.	☒	☐	☐	☐
	Result 2		☐	☐	☐	☐
	Result 3		☐	☐	☐	☐
	**Outcome 3:** 30-day mortality		**Yes**	**No**	**Unclear**	**N/A**
	Result 1: 35.8% (pre) → 35.3% (post), NS	Mortality proportions were compared using appropriate categorical statistical tests (χ^2/Fisher's).	☒	☐	☐	☐

		Yes	No	Unclear	N/A
Result 2		☐	☐	☐	☐
Result 3		☐	☐	☐	☐
**Outcome 4:** Meropenem consumption (DDD)		**Yes**	**No**	**Unclear**	**N/A**
Result 1: 585 DDD → 232 DDD, *p < 0.001*	Continuous DDD values were compared using proper trend and comparative tests.	☒	☐	☐	☐
Result 2		☐	☐	☐	☐
Result 3		☐	☐	☐	☐
**Outcome 5:** Microbiological failure		**Yes**	**No**	**Unclear**	**N/A**
Result 1: 9.9% (pre) → 10.3% (post), *p = 0.005*	Proportions were analyzed with χ^2/Fisher's exact test; fully appropriate.	☒	☐	☐	☐
Result 2		☐	☐	☐	☐
Result 3		☐	☐	☐	☐
**Outcome 6:** New 30-day MDRO colonization		**Yes**	**No**	**Unclear**	**N/A**
Result 1: 36.6% (pre) → 8.3% (post), *p < 0.001*	Correct categorical statistics were used to compare colonization rates between phases.	☒	☐	☐	☐
Result 2		☐	☐	☐	☐
Result 3		☐	☐	☐	☐
**Outcome 7**		**Yes**	**No**	**Unclear**	**N/A**

Result 1		☐	☐	☐	☐
Result 2		☐	☐	☐	☐
Result 3		☐	☐	☐	☐

Overall appraisal: **Include:** ☒ **Exclude:** ☐ **Seek Further Info:** ☐

Comments:

The study demonstrated strong, consistent improvements in key antimicrobial stewardship and clinical outcomes, including appropriate empirical therapy, 7-day clinical cure, microbiological failure, MDRO colonization, and meropenem consumption. Outcomes were measured reliably with complete follow-up, and statistical analyses were appropriate for all comparisons. The intervention was clearly defined, consistently implemented, and supported by robust pre–post data.

Limitations:

Non-concurrent historical control (2018 vs 2022), potential changes in ICU case-mix or microbiology epidemiology over time, and single-center design may limit generalizability.

© JBI, 2022. All rights reserved. JBI grants use of these tools for research purposes only. All other enquiries should be sent to jbisynthesis@adelaide.edu.au
