# Supplementary material for: Impact of Pharmacist-Involved Antimicrobial Stewardship Interventions on Clinical and Process Outcomes in Multidrug-Resistant Organism Infections: A Systematic Review and Meta-Analysis
Source: Antibiotics (Basel). 2026 Sep 25;15(10):955. doi: 10.3390/antibiotics15100955 (PMC13700879; doi:10.3390/antibiotics15100955)
Supplement: Supplementary file 1 [file antibiotics-15-00955-s001.zip › Supplementary File S3.pdf]

Supplementary File S3. Standardized Summary of Process Outcomes Across Tier A/B Studies

No.	Study (Tier)	Time to Appropriate/Active Therapy	De-escalation / Escalation	Antimicrobial Utilization (DOT/DDD/AUD)	Culture Follow-up / TDM	Direction of Effect
1	Ohashi et al. [26] (A)	Early anti-MRSA therapy: 62.3%→82.4%, p=.038<br>Appropriate treatment duration: 44.8%→72.1%, p=.027	NR	NR	Negative follow-up cultures: 40%→80%, p<.001	Positive
2	McCrink et al. [31] (A)	103h [76–156]→75h [56–100], p<.001	NR	NR	Time to bacteremia clearance: 74h→67h, p=.841 (NS)	Positive (therapy timing)
3	Swingler et al. [32] (A)	22.8h [3.7–63.8]→16.4h [2.9–19.3], p=.001	NR	NR	NR	Positive
4	Wong et al. [29] (A)	NR	NR	Ertapenem DOT/1000 APD: 0.0145→0.0078, p<.01 (−19%)	NR	Positive (utilization)
5	Yamada et al. [27] (A)	Improved (not further quantified)	Acceptance rate: 133/145 (91.7%)	Carbapenem DOT/1000: 33.2±5.7→25.5±4.6 (↓); TAZ/PIPC: 18±4.8→23.3±2.7 (↑, significant)	Anti-MRSA TDM monitored; no outcome-impact data reported	Positive
6	Nakamura et al. [28] (A)	NR	Rate: 0/288 (pre)→7/247 (post) (↑)	AUD/DOT — Carbapenem: 0.37±0.07→0.60±0.12, p<.01; TAZ/PIPC: 0.73±0.08→0.67±0.07, p=.15 (NS)	TDM: 3/288 (pre)→5/247 (post) (↑)	Positive
7	Andrade et al. [30] (A)	Time from positive culture to review: significant reduction, p<.001 (not further quantified)	NR	NR	NR	Positive
8	Smith et al. [35] (B)	≤24h: 65/86 (76%)→169/172 (98%), p<.001; median 14.19h→1.48h	NR	NR	NR	Positive
9	Li et al. [34] (B)	NR	NR	DOT: median 5 [3–9.5]→4 [2–7], p=.030 (↓)	NR	Positive (utilization)
10	Mahrous et al. [37] (B)	Median 41 (27.7–64)→18.1 (8.7–27) h; MRSA subgroup 39→9h	De-escalation: 41/164 (25%) vs 31/148 (21%), RR† 0.84; Escalation: 23/164 (14%) vs 13/148 (9%), RR† 0.63	NR	NR	Positive
11	Sadyrbaeva-Dolgova et al. [36] (B)	NR	De-escalation appropriateness: unadjusted OR 0.40 (0.19–0.84); adjusted OR‡ 0.33 (0.15–0.74)	Overall DOT: de-escalation 13 (10–17) vs no de-escalation 9 (6–13) days; carbapenem-specific 4 (2–6) vs 6 (4–10) days	NR	Positive
12	Knudsen & Andersen [33] (B)	NR	NR	NR	ESBL-KP incidence: significant reduction, p<.001 (rate not further quantified)	Positive

13	Rinaldi et al. [38] (B)	Appropriate DOT: 12 [20]→9 [15]; 16→12 days overall, p<.001	Targeted de-escalation: 8/67 (11.9%)→14/68 (20.6%), NS; Escalation: 3/67 (4.5%)→2/68 (2.9%)	Meropenem: 585→232 total DDD, p<.001; piperacillin-tazobactam: 105→168 DDD, p<.002	TDM-guided dose reduction: 43/68 (63.2%) post-phase	Mixed (utilization ↑, de-escalation NS)

Abbreviations: AUD, antimicrobial use density; DDD, defined daily dose; DOT, days of therapy; NR, not reported; NS, not significant; TAZ/PIPC, piperacillin-tazobactam; TDM, therapeutic drug monitoring. † calculated by the review team from raw counts reported in the original study; ‡ adjusted estimate as reported by the original study.
