# Supplementary material for: Impact of Pharmacist-Involved Antimicrobial Stewardship Interventions on Clinical and Process Outcomes in Multidrug-Resistant Organism Infections: A Systematic Review and Meta-Analysis
Source: Antibiotics (Basel). 2026 Sep 25;15(10):955. doi: 10.3390/antibiotics15100955 (PMC13700879; doi:10.3390/antibiotics15100955)
Supplement: Supplementary file 1 [file antibiotics-15-00955-s001.zip › Supplementary File S4.pdf]

Supplementary File S4. Search Strategies of Individual Databases

Database: Pubmed

Results: 2348

Search Strategy:

((Antimicrobial stewardship[MeSH Terms]) OR (Antimicrobial stewardship program[Title/Abstract]) OR (Antimicrobial stewardship[Title/Abstract]) OR (AMS[Title/Abstract]) OR (Antibacterial stewardship[Title/Abstract]) OR (Antibiotic stewardship[Title/Abstract]) OR (ASP[Title/Abstract])) AND ((Pharmacists[MeSH Terms]) OR (Pharmacist*[Title/Abstract]) OR (Pharmac*[Title/Abstract]) OR (Pharmacist-led[Title/Abstract]) OR (Pharmacist-driven[Title/Abstract]) OR (Pharmacist-involved[Title/Abstract]) OR (Pharmacy service, hospital[MeSH Terms]) OR (Pharmacy[MeSH Terms]) OR (Pharmacy[Title/Abstract]) OR (Faculty, Pharmacy[MeSH Terms]) OR (Pharmacy faculty[Title/Abstract]) OR (Students, Pharmacy[MeSH Terms]) OR (Pharmacy Student[Title/Abstract]) OR (Patient Care Team[MeSH Terms]) OR (Interdisciplinary[Title/Abstract]) OR (Multidisciplinary[Title/Abstract])) AND ((Multidrug resistant organism*[Title/Abstract]) OR (Multi drug resistant organism*[Title/Abstract]) OR (Resistan*[Title/Abstract]) OR (Drug Resistance, Multiple[MeSH Terms]) OR (Drug Resistance, Multiple, Bacterial[MeSH Terms]) OR (Drug Resistance, Bacterial[MeSH Terms]) OR (Drug Resistance, Microbial[MeSH Terms]) OR (MDRO*[Title/Abstract]) OR (MDR[Title/Abstract]) OR (Infections[MeSH Terms]) OR (infection*[Title/Abstract]) OR (Methicillin resistant staphylococcus aureus[MeSH Terms]) OR (Methicillin resistant staphylococcus aureus[Title/Abstract]) OR (MRSA[Title/Abstract]) OR (Meticillin resistant staphylococcus aureus[Title/Abstract]) OR (beta-Lactamases[MeSH Terms]) OR (Extended spectrum beta lactamase*[Title/Abstract]) OR (ESBL*[Title/Abstract]) OR (Enterobacteriaceae[MeSH Terms]) OR (Enterobacterales[Title/Abstract]) OR (Carbapenem resistant Enterobacterales[Title/Abstract]) OR (Carbapenem resistant enterobacteriaceae[Title/Abstract]) OR (CRE[Title/Abstract]) OR (Pseudomonas aeruginosa[MeSH Terms]) OR (Difficult-to-Treat Pseudomonas[Title/Abstract]) OR (DTR Pseudomonas[Title/Abstract]) OR (Acinetobacter baumannii[MeSH Terms]) OR (Carbapenem-resistant Acinetobacter[Title/Abstract]) OR (CRAB[Title/Abstract]) OR (DTR-PA[Title/Abstract]) OR (Carbapenem resistant enterobacteriaceae[MeSH Terms]))

Database: Web of Science

Results: 2445

Search Strategy:

((TS=("Antimicrobial stewardship") OR TS=("Antimicrobial stewardship program") OR TS=("AMS") OR TS=("Antibacterial stewardship") OR TS=("Antibiotic stewardship") OR TS=("ASP"))) AND ((TS=("Pharmacist*") OR TS=("Pharmac*") OR TS=("Pharmacist-led") OR TS=("Pharmacist led") OR TS=("Pharmacist-driven") OR TS=("Pharmacist driven") OR TS=("Pharmacist-involved") OR TS=("Pharmacist involved") OR TS=("Pharmacy") OR TS=("Pharmacy Student") OR TS=("Interdisciplinary") OR TS=("Multidisciplinary"))) AND ((TS=("Multidrug resistant organism*") OR TS=("Multi drug resistant organism*") OR TS=("Multi-drug resistant organism*") OR TS=("Resistan*") OR TS=("MDRO*") OR TS=("MDR") OR TS=("infection*") OR TS=("Methicillin resistant staphylococcus aureus") OR TS=("Methicillin-resistant staphylococcus aureus") OR TS=("MRSA") OR TS=("Meticillin resistant staphylococcus aureus") OR TS=("Meticillin-resistant staphylococcus aureus") OR TS=("Extended spectrum beta lactamase*") OR TS=("ESBL*") OR TS=("Enterobacterales") OR TS=("Carbapenem resistant Enterobacterales") OR TS=("Carbapenem-resistant Enterobacterales") OR TS=("Carbapenem resistant enterobacteriaceae") OR TS=("Carbapenem-resistant enterobacteriaceae") OR TS=("CRE") OR TS=("Difficult-to-Treat Pseudomonas") OR TS=("DTR Pseudomonas") OR TS=("DTR-Pseudomonas") OR TS=("DTR-PA") OR TS=("Carbapenem-resistant Acinetobacter") OR TS=("Carbapenem resistant Acinetobacter") OR TS=("CRAB")))

Terms:

Concept 1:

1- (Antimicrobial stewardship) OR
2- (Antimicrobial stewardship program) OR
3- (AMS) OR
4- (Antibacterial stewardship) OR
5- (Antibiotic stewardship) OR
6- (ASP)

AND

Concept 2:

1- (Pharmacist*) OR

2- (Pharmac*) OR
3- (Pharmacist-led) OR
4- (Pharmacist led) OR
5- (Pharmacist-driven) OR
6- (Pharmacist driven) OR
7- (Pharmacist-involved) OR
8- (Pharmacist involved) OR
9- (Pharmacy) OR
10- (Pharmacy Student) OR
11- (Interdisciplinary) OR
12- (Multidisciplinary)

AND

Concept 3:

1- (Multidrug resistant organism*) OR
2- (Multi drug resistant organism*) OR
3- (Multi-drug resistant organism*) OR
4- (Resistan*) OR
5- (MDRO*) OR
6- (MDR) OR
7- (infection*) OR
8- (Methicillin resistant staphylococcus aureus) OR
9- (Methicillin-resistant staphylococcus aureus) OR
10- (MRSA) OR
11- (Meticillin resistant staphylococcus aureus) OR
12- (Meticillin-resistant staphylococcus aureus) OR
13- (Extended spectrum beta lactamase*) OR
14- (ESBL*) OR
15- (Enterobacterales) OR
16- (Carbapenem resistant Enterobacterales) OR
17- (Carbapenem-resistant Enterobacterales) OR
18- (Carbapenem resistant enterobacteriaceae) OR
19- (Carbapenem-resistant enterobacteriaceae) OR
20- (CRE) OR
21- (Difficult-to-Treat Pseudomonas) OR
22- (DTR Pseudomonas) OR
23- (DTR-Pseudomonas) OR

24- (DTR-PA) OR
25- (Carbapenem-resistant Acinetobacter) OR
26- (Carbapenem resistant Acinetobacter) OR
27- (CRAB)

Database: ERIC

Results = 1

Search Strategy:

(("antimicrobial stewardship" OR "antibiotic stewardship" OR "antibacterial stewardship" OR "AMS" OR "ASP") AND ("drug resistance" OR "antimicrobial resistance" OR "antibiotic resistance" OR "multidrug-resistant" OR "MDRO" OR "MDR bacteria" OR "resistant bacteria" OR "ESBL" OR "carbapenem-resistant" OR "KPC" OR "NDM" OR "OXA-48" OR "VIM" OR "IMP" OR "MRSA" OR "VRE" OR "CRAB" OR "DTR Pseudomonas" OR "Clostridioides difficile" OR "C. difficile" OR "CDI") AND (Pharmacist* OR Pharmac* OR "Pharmacist-led" OR "Pharmacist-driven" OR "Pharmacist-involved" OR "Pharmacy" OR "Pharmacy service" OR "Pharmacy student" OR "Patient care" OR Interdisciplinary OR Multidisciplinary))

(("antimicrobial stewardship" OR "antibiotic stewardship" OR "antibacterial stewardship" OR "AMS" OR "ASP") AND ("drug resistance" OR "antimicrobial resistance" OR "antibiotic resistance" OR "multidrug-resistant" OR "MDRO" OR "MDR bacteria" OR "resistant bacteria" OR "ESBL" OR "carbapenem-resistant" OR "MRSA" OR "CRAB" OR "DTR Pseudomonas") AND (Pharmacist* OR Pharmac* OR "Pharmacist-led" OR "Pharmacist-driven" OR "Pharmacist-involved" OR "Pharmacy" OR "Pharmacy service" OR "Pharmacy student" OR "Patient care" OR Interdisciplinary OR Multidisciplinary))

Database: Scopus

Results: 4000

Search Strategy:

((TITLE-ABS-KEY (Antimicrobial stewardship)) OR (TITLE-ABS-KEY (Antimicrobial stewardship program)) OR (TITLE-ABS-KEY (AMS)) OR (TITLE-ABS-KEY (Antibacterial stewardship)) OR (TITLE-ABS-KEY (Antibiotic stewardship)) OR (TITLE-ABS-KEY (ASP))) AND ((TITLE-ABS-KEY (Pharmacist*)) OR (TITLE-ABS-KEY (Pharmac*)) OR (TITLE-ABS-KEY (Pharmacist-led)) OR (TITLE-ABS-KEY (Pharmacist led)) OR (TITLE-ABS-KEY (Pharmacist-driven)) OR (TITLE-ABS-KEY (Pharmacist driven)) OR (TITLE-ABS-KEY (Pharmacist-involved)) OR (TITLE-ABS-KEY (Pharmacist involved)) OR (TITLE-ABS-KEY (Pharmacy)) OR (TITLE-ABS-

KEY (Pharmacy student)) OR (TITLE-ABS-KEY (Interdisciplinary)) OR (TITLE-ABS-KEY (Multidisciplinary))) AND ((TITLE-ABS-KEY (Multidrug resistant organism*)) OR (TITLE-ABS-KEY (Multi drug resistant organism*)) OR (TITLE-ABS-KEY (Multi-drug resistant organism*)) OR (TITLE-ABS-KEY (Resistan*)) OR (TITLE-ABS-KEY (MDRO*)) OR (TITLE-ABS-KEY (MDR)) OR (TITLE-ABS-KEY (infection*)) OR (TITLE-ABS-KEY (Methicillin resistant staphylococcus aureus)) OR (TITLE-ABS-KEY (Methicillin-resistant staphylococcus aureus)) OR (TITLE-ABS-KEY (MRSA)) OR (TITLE-ABS-KEY (Meticillin resistant staphylococcus aureus)) OR (TITLE-ABS-KEY (Meticillin-resistant staphylococcus aureus)) OR (TITLE-ABS-KEY (Extended spectrum beta lactamase*)) OR (TITLE-ABS-KEY (ESBL*)) OR (TITLE-ABS-KEY (Enterobacterales)) OR (TITLE-ABS-KEY (Carbapenem resistant Enterobacterales)) OR (TITLE-ABS-KEY (Carbapenem resistant enterobacteriaceae)) OR (TITLE-ABS-KEY (Carbapenem-resistant enterobacteriaceae)) OR (TITLE-ABS-KEY (Carbapenem-resistant enterobacterales)) OR (TITLE-ABS-KEY (CRE)) OR (TITLE-ABS-KEY (Difficult-to-Treat Pseudomonas)) OR (TITLE-ABS-KEY (DTR Pseudomonas)) OR (TITLE-ABS-KEY (DTR-Pseudomonas)) OR (TITLE-ABS-KEY (DTR-PA)) OR (TITLE-ABS-KEY (Carbapenem-resistant Acinetobacter)) OR (TITLE-ABS-KEY (Carbapenem resistant Acinetobacter)) OR (TITLE-ABS-KEY (CRAB)))

Concept 1:

7- (Antimicrobial stewardship) OR
8- (Antimicrobial stewardship program) OR
9- (AMS) OR
10- (Antibacterial stewardship) OR
11- (Antibiotic stewardship) OR
12- (ASP)

AND

Concept 2:

13- (Pharmacist*) OR
14- (Pharmac*) OR
15- (Pharmacist-led) OR
16- (Pharmacist led) OR
17- (Pharmacist-driven) OR
18- (Pharmacist driven) OR
19- (Pharmacist-involved) OR

20- (Pharmacist involved) OR
21- (Pharmacy) OR
22- (Pharmacy Student) OR
23- (Interdisciplinary) OR
24- (Multidisciplinary))

AND

Concept 3:

28- (Multidrug resistant organism*) OR
29- (Multi drug resistant organism*) OR
30- (Multi-drug resistant organism*) OR
31- (Resistan*) OR
32- (MDRO*) OR
33- (MDR) OR
34- (infection*) OR
35- (Methicillin resistant staphylococcus aureus) OR
36- (Methicillin-resistant staphylococcus aureus) OR
37- (MRSA) OR
38- (Meticillin resistant staphylococcus aureus) OR
39- (Meticillin-resistant staphylococcus aureus) OR
40- (Extended spectrum beta lactamase*) OR
41- (ESBL*) OR
42- (Enterobacterales) OR
43- (Carbapenem resistant Enterobacterales) OR
44- (Carbapenem-resistant Enterobacterales) OR
45- (Carbapenem resistant enterobacteriaceae) OR
46- (Carbapenem-resistant enterobacteriaceae) OR
47- (CRE) OR
48- (Difficult-to-Treat Pseudomonas) OR
49- (DTR Pseudomonas) OR
50- (DTR-Pseudomonas) OR
51- (DTR-PA) OR
52- (Carbapenem-resistant Acinetobacter) OR
53- (Carbapenem resistant Acinetobacter) OR
54- (CRAB)

Database: Cinahl

Results: 670

Search Strategy:

(MH (antimicrobial stewardship) OR XB (antimicrobial stewardship program) OR XB (antimicrobial stewardship) OR XB (AMS) OR XB (antibacterial stewardship) OR XB (antibiotic stewardship) OR XB (ASP)) AND (MH (pharmacists or pharmacist) OR XB (pharmacist-led) OR XB (pharmac*) OR XB (pharmacist-driven) OR XB (pharmacist-involved) OR MH (pharmacy service, hospital) OR MH (pharmacy) OR XB (pharmacy) OR MH (faculty, pharmacy) OR XB (pharmacy faculty) OR MH (patient care team) OR XB (interdisciplinary*) OR XB (multidisciplinary*)) AND (MH (multidrug resistant organisms) OR XB (multidrug resistant organism*) OR XB (resistan*) OR MH (drug resistance, multiple) OR MH (drug resistance, multiple, bacterial) OR MH (drug resistance, bacterial) OR MH (drug resistance, microbial) OR XB (MDRO*) OR XB (MDR) OR MH (infections or infection) OR XB (infection*) OR MH (methicillin-resistant staphylococcus aureus) OR XB (meticillin-resistant staphylococcus aureus) OR XB (MRSA) OR XB (methicillin-resistant staphylococcus aureus) OR XB (beta-lactamases) OR XB (extended spectrum beta-lactamase) OR XB (ESBL*) OR MH (enterobacteriaceae) OR XB (enterobacteriaceae) OR XB (enterobacterales) OR XB (carbapenem-resistant enterobacteriaceae) OR XB (carbapenem-resistant enterobacterales) OR XB (CRE) OR MH (pseudomonas aeruginosa) OR XB (difficult to treat pseudomonas) OR XB (DTR pseudomonas) OR MH (acinetobacter baumannii) OR XB (carbapenem resistant acinetobacter) OR XB (CRAB) OR XB (DTR-PA) OR MH (carbapenem-resistant enterobacteriaceae))

Database: Embase

Results: 4924

Search Strategy:

(('antimicrobial stewardship'/exp) OR ('antimicrobial stewardship program':ti,ab,kw) OR ('antimicrobial stewardship':ti,ab,kw) OR ('ams':ti,ab,kw) OR ('antibacterial stewardship':ti,ab,kw) OR ('antibiotic stewardship':ti,ab,kw) OR ('asp':ti,ab,kw)) AND (('pharmacist'/exp) OR ('pharmacist*':ti,ab,kw) OR ('pharmac*':ti,ab,kw) OR ('pharmacist-led':ti,ab,kw) OR ('pharmacist-driven':ti,ab,kw) OR ('pharmacist-involved':ti,ab,kw) OR ('hospital pharmacy'/exp) OR ('patient care team'/exp) OR ('interdisciplinary':ti,ab,kw) OR ('multidisciplinary':ti,ab,kw)) AND (('multidrug resistant organism*':ti,ab,kw) OR ('multi drug resistant organism*':ti,ab,kw) OR ('resistan*':ti,ab,kw) OR ('multidrug resistance'/exp) OR ('multidrug resistance'/exp) OR ('antibiotic resistance'/exp) OR ('antibiotic resistance'/exp) OR ('mdro*':ti,ab,kw) OR ('mdr':ti,ab,kw) OR ('infection'/exp) OR ('infection*':ti,ab,kw) OR ('methicillin resistant Staphylococcus aureus'/exp) OR ('methicillin resistant staphylococcus aureus':ti,ab,kw) OR ('mrsa':ti,ab,kw) OR ('meticillin resistant staphylococcus aureus':ti,ab,kw)

OR ('beta lactamase'/exp) OR ('extended spectrum beta lactamase*':ti,ab,kw) OR ('esbl*':ti,ab,kw) OR ('Enterobacteriaceae'/exp) OR ('enterobacterales':ti,ab,kw) OR ('carbapenem resistant enterobacterales':ti,ab,kw) OR ('carbapenem resistant enterobacteriaceae':ti,ab,kw) OR ('cre':ti,ab,kw) OR ('Pseudomonas aeruginosa'/exp) OR ('difficult-to-treat pseudomonas':ti,ab,kw) OR ('dtr pseudomonas':ti,ab,kw) OR ('Acinetobacter baumannii'/exp) OR ('carbapenem-resistant acinetobacter':ti,ab,kw) OR ('crab':ti,ab,kw) OR ('dtr-pa':ti,ab,kw) OR ('carbapenem-resistant Enterobacteriaceae'/exp))

Database: Proquest

10/10/2025

Results: 2321

Search Strategy:

(antimicrobial stewardship OR antimicrobial stewardship program OR antibacterial stewardship OR antibiotic stewardship OR AMS OR ASP) AND subject(pharmacist* OR pharmac* OR pharmacist-led OR pharmacist-driven OR pharmacist-involved OR pharmacy OR pharmacy faculty OR pharmacy student OR interdisciplinary OR multidisciplinary OR patient care team OR hospital pharmacy service) AND subject(multidrug resistant organism* OR resistan* OR MDRO* OR MDR OR infection* OR methicillin resistant staphylococcus aureus OR MRSA OR meticillin resistant staphylococcus aureus OR beta-lactamase* OR extended spectrum beta lactamase* OR ESBL* OR Enterobacteriaceae OR Enterobacterales OR carbapenem resistant enterobacterales OR CRE OR pseudomonas aeruginosa OR difficult-to-treat pseudomonas OR DTR pseudomonas OR DTR-PA OR Acinetobacter baumannii OR carbapenem resistant acinetobacter OR CRAB)
